# Supplementary material for: Development of Coumarin-Based Hydroxamates as Histone Deacetylase Inhibitors with Antitumor Activities
Source: Molecules. 2020 Feb 7;25(3):717. doi: 10.3390/molecules25030717 (PMC7036849; doi:10.3390/molecules25030717)
Supplement: Supplementary file 1 [file molecules-25-00717-s001.pdf]

Supplementary Information for

Development of Coumarin-based Hydroxamates as Histone Deacetylase Inhibitors with Antitumor Activities

Na Zhao [1,2], Feifei Yang [2,*], Lina Han [2], Yuhua Qu [2], Di Ge [2] and Hua Zhang [2,*]

[1] School of Chemistry and Chemical Engineering, University of Jinan, Jinan 250022, China; zhaona3702@163.com

[2] School of Biological Science and Technology, University of Jinan, Jinan 250022, China; hanln95@163.com (L.H.); 17854175513@163.com (Y.Q.); gedi_blue@126.com (D.G.)

* Correspondence: bio_yangff@ujn.edu.cn (F.Y.) ; bio_zhangh@ujn.edu.cn (H.Z.); Tel.: 86-0531-89736199 (H.Z.).

Table of contents

Spectra of ^{1}H and ^{13}C NMR and HR MS and HPLC of target compounds.

Spectra of ^{1}H and ^{13}C NMR and HR MS and HPLC of target compound **13a**…....S3-4

Spectra of ^{1}H and ^{13}C NMR and HR MS and HPLC of target compound **13b**…....S5-6

Spectra of ^{1}H and ^{13}C NMR and HR MS and HPLC of target compound **13c**…....S7-8

Spectra of ^{1}H and ^{13}C NMR and HR MS and HPLC of target compound **13d**…..S9-10

Spectra of ^{1}H and ^{13}C NMR and HR MS and HPLC of target compound **13e**….S11-12

Spectra of ^{1}H and ^{13}C NMR and HR MS and HPLC of target compound **13f**….S13-14

Spectra of ^{1}H and ^{13}C NMR and HR MS and HPLC of target compound **13g**....S15-16

Spectra of ^{1}H and ^{13}C NMR and HR MS and HPLC of target compound **14a**....S17-18

Spectra of ^{1}H and ^{13}C NMR and HR MS and HPLC of target compound **14b**....S19-20

Spectra of ^{1}H and ^{13}C NMR and HR MS and HPLC of target compound **14c**....S21-22

Spectra of ^{1}H and ^{13}C NMR and HR MS and HPLC of target compound **14d**....S23-24

Spectra of ^{1}H and ^{13}C NMR and HR MS and HPLC of target compound **14e**....S25-26

Spectra of ^{1}H and ^{13}C NMR and HR MS and HPLC of target compound **14f** ...S27-28

Spectra of ^{1}H and ^{13}C NMR and HR MS and HPLC of target compound **14g**....S29-30

Spectra of ^{1}H and ^{13}C NMR and HR MS and HPLC of target compound **14h**....S31-32

Spectra of ^{1}H and ^{13}C NMR and HR MS and HPLC of target compound **14i**....S33-34

Spectra of ^{1}H and ^{13}C NMR and HR MS and HPLC of target compound **14j**....S35-36

Spectra of ^{1}H and ^{13}C NMR and HR MS and HPLC of target compound **14k**....S37-38

Spectra of ^{1}H and ^{13}C NMR and HR MS and HPLC of target compound **14l**....S39-40

Spectra of ^{1}H and ^{13}C NMR and HR MS and HPLC of target compound **14m**...S41-42

Spectra of ^{1}H and ^{13}C NMR and HR MS and HPLC of target compound **14n**....S43-44

Spectra of ^{1}H and ^{13}C NMR and HR MS and HPLC of target compound **14o**....S45-46

Spectra of ^{1}H and ^{13}C NMR and HR MS and HPLC of target compound **14p**....S47-48

Spectra of ^{1}H and ^{13}C NMR and HR MS and HPLC of target compound **14q**....S49-50

Spectra of ^{1}H and ^{13}C NMR and HR MS and HPLC of target compound **14r**....S51-52

Spectra of ^{1}H and ^{13}C NMR and HR MS and HPLC of target compound **14s**....S53-54

Compound 13a

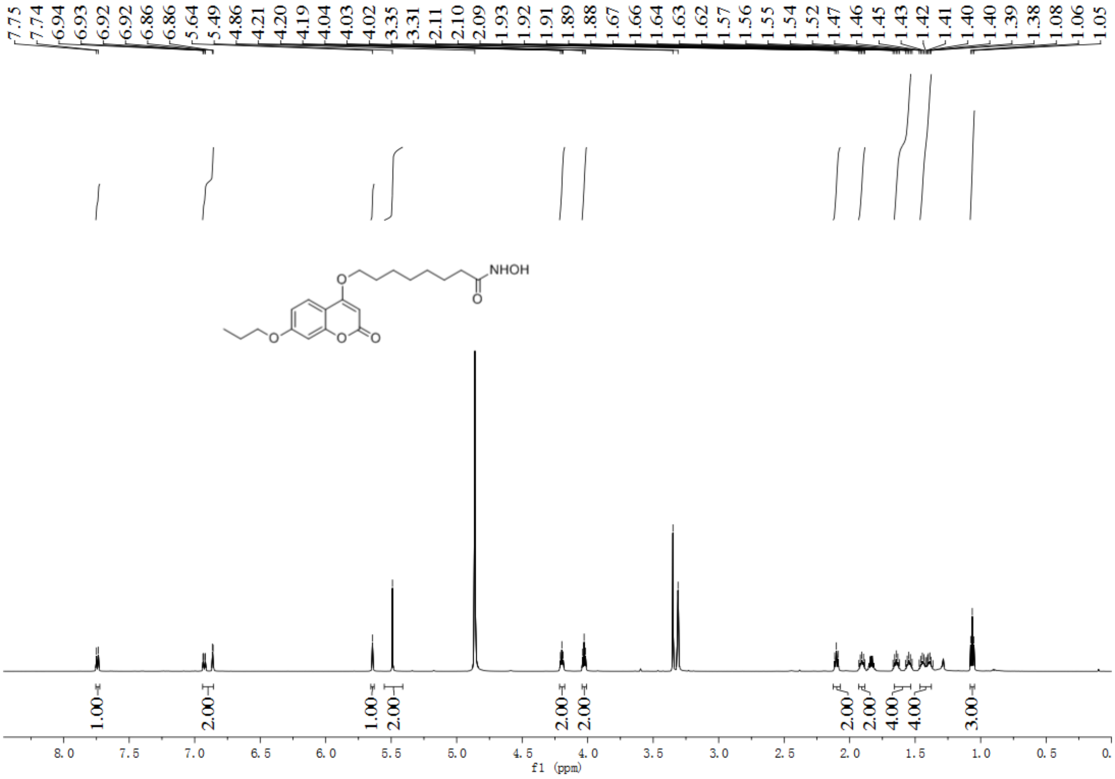

Fig.1. The ^{1}H NMR spectrum for **13a**

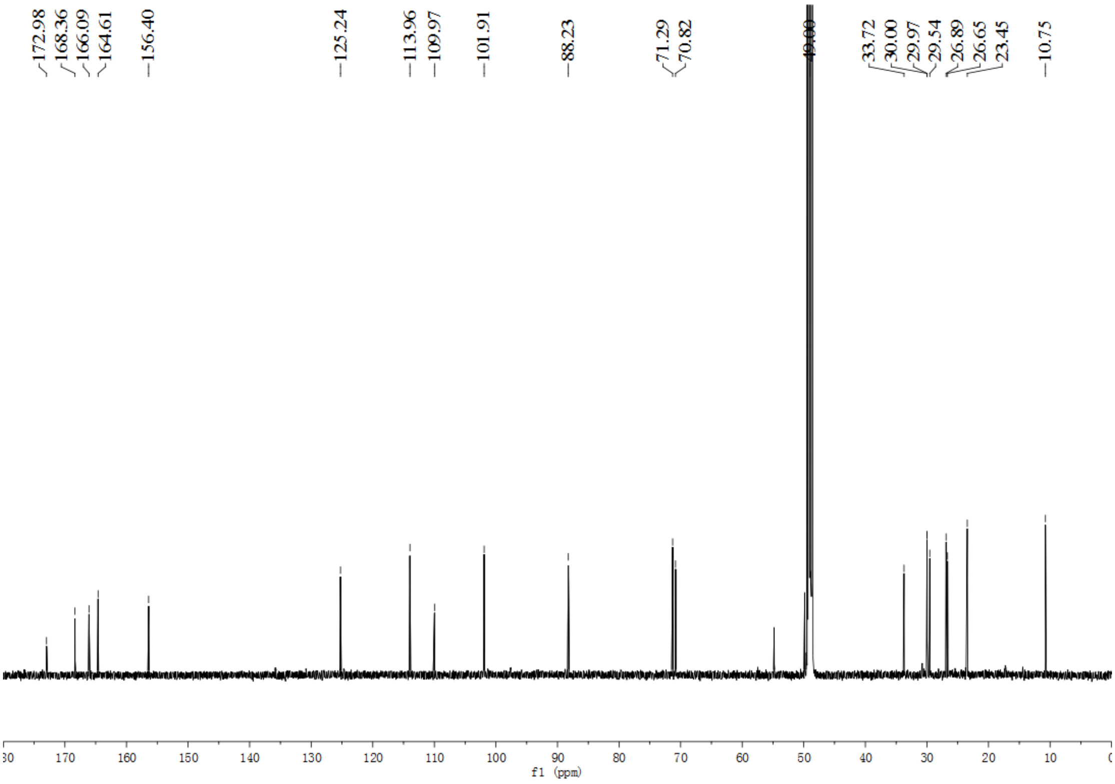

Fig.2. The ^{13}C NMR spectrum for **13a**

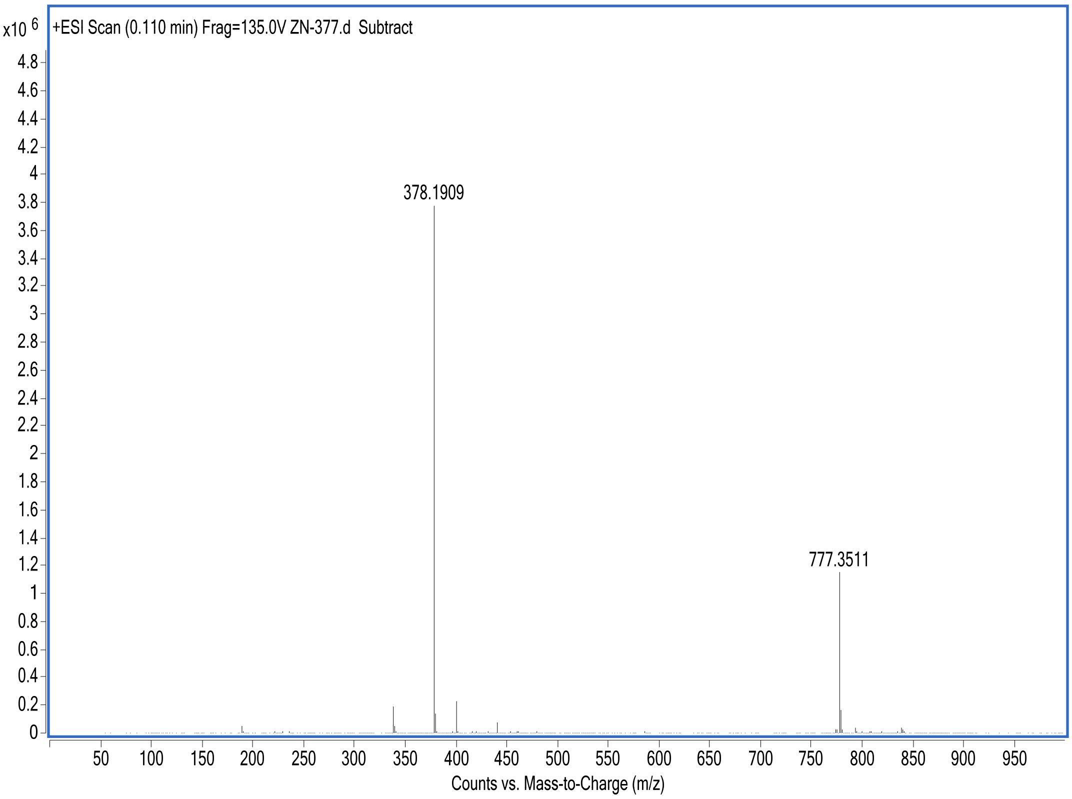

Fig.3. The HR MS spectrum for **13a**

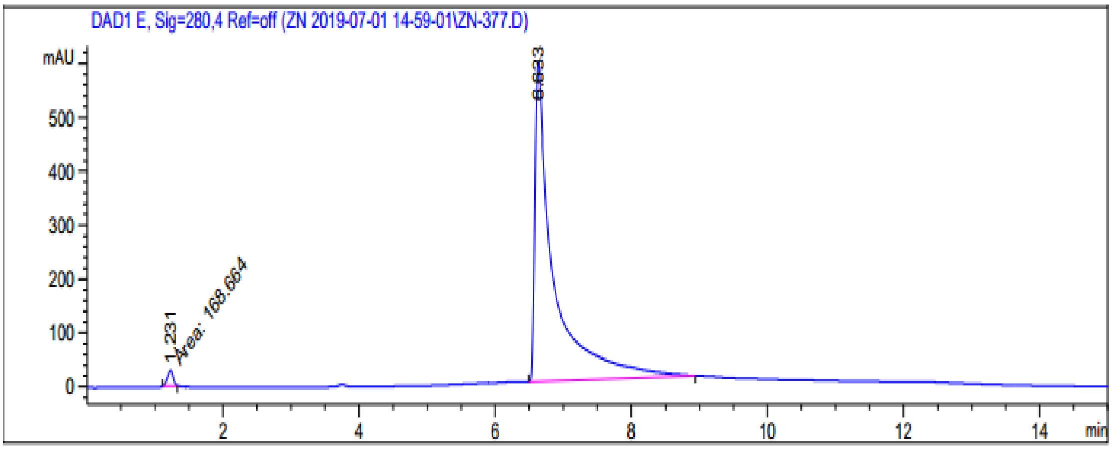

Signal 2: DAD1 E, Sig=280,4 Ref=off

Peak #	RetTime [min]	Type	Width [min]	Area [mAU*s]	Height [mAU]	Area %
1	1.231	MM	0.0961	168.66426	29.25032	1.4547
2	6.633	BB	0.2563	1.14258e4	594.21375	98.5453
Totals :				1.15945e4	623.46407	

Fig.4. The HPLC for **13a**

Compound 13b

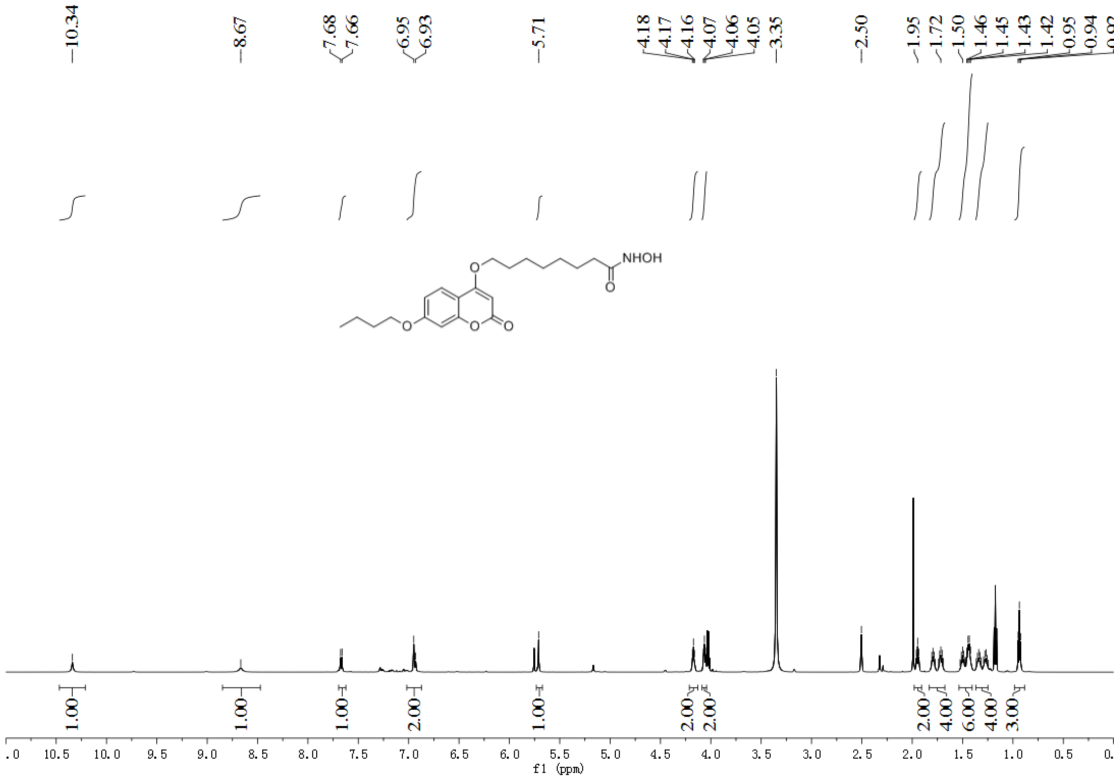

Fig.5. The ^{1}H NMR spectrum for **13b**

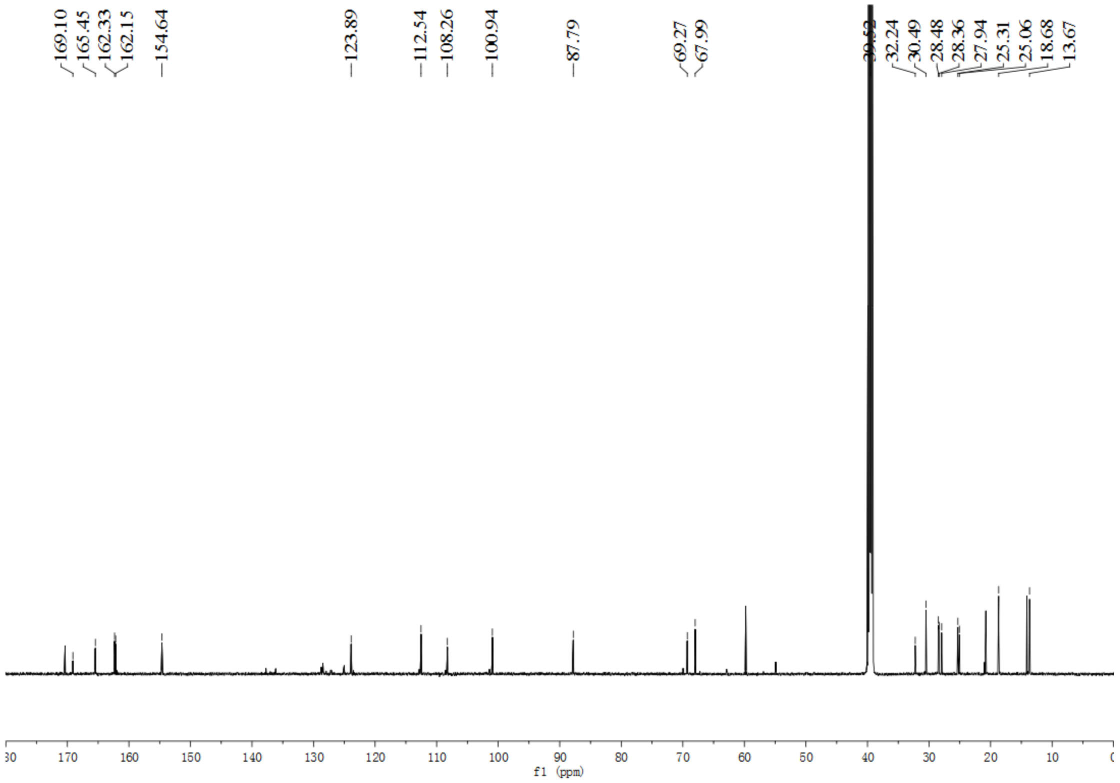

Fig.6. The ^{13}C NMR spectrum for **13b**

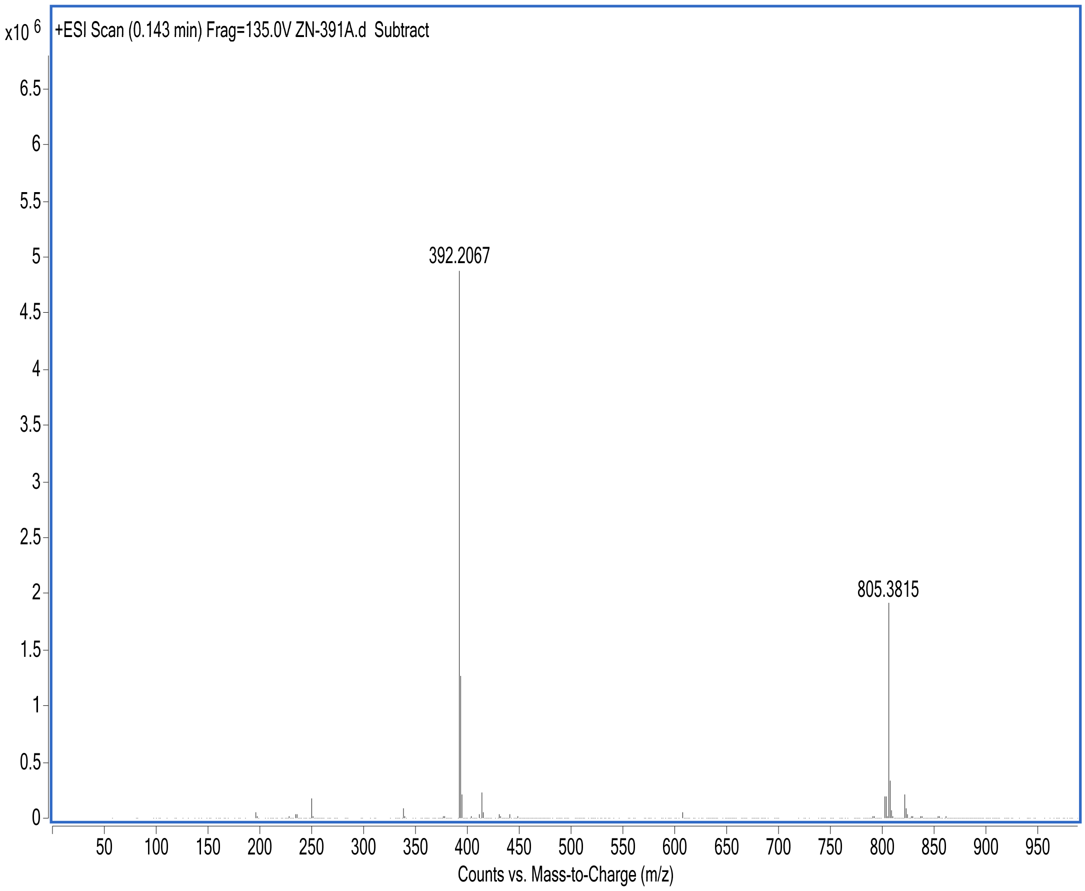

Fig.7. The HR MS spectrum for **13b**

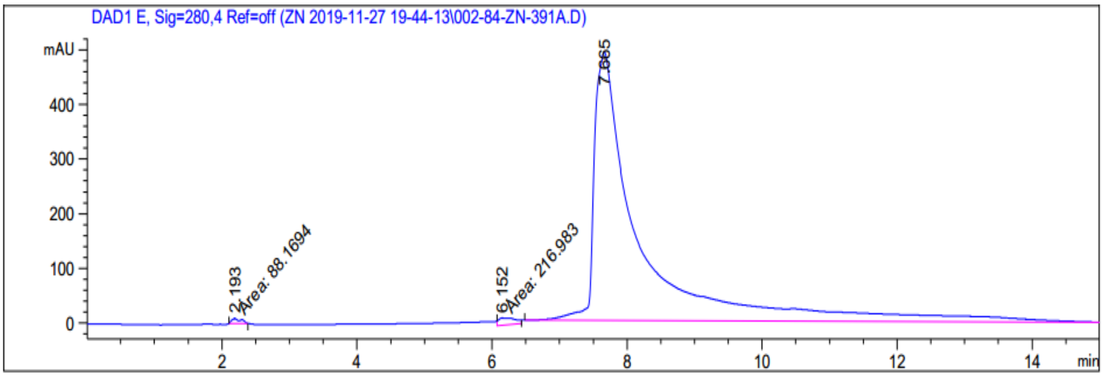

Peak #	RetTime [min]	Type	Width [min]	Area [mAU*s]	Height [mAU]	Area %
1	2.193	PP	0.1643	88.16940	8.94623	0.3733
2	6.152	MM	0.2746	216.98256	13.16806	0.9188
3	7.665	BV R	0.5935	2.33110e4	492.40826	98.7079

Fig.8. The HPLC for **13b**

Compound 13c

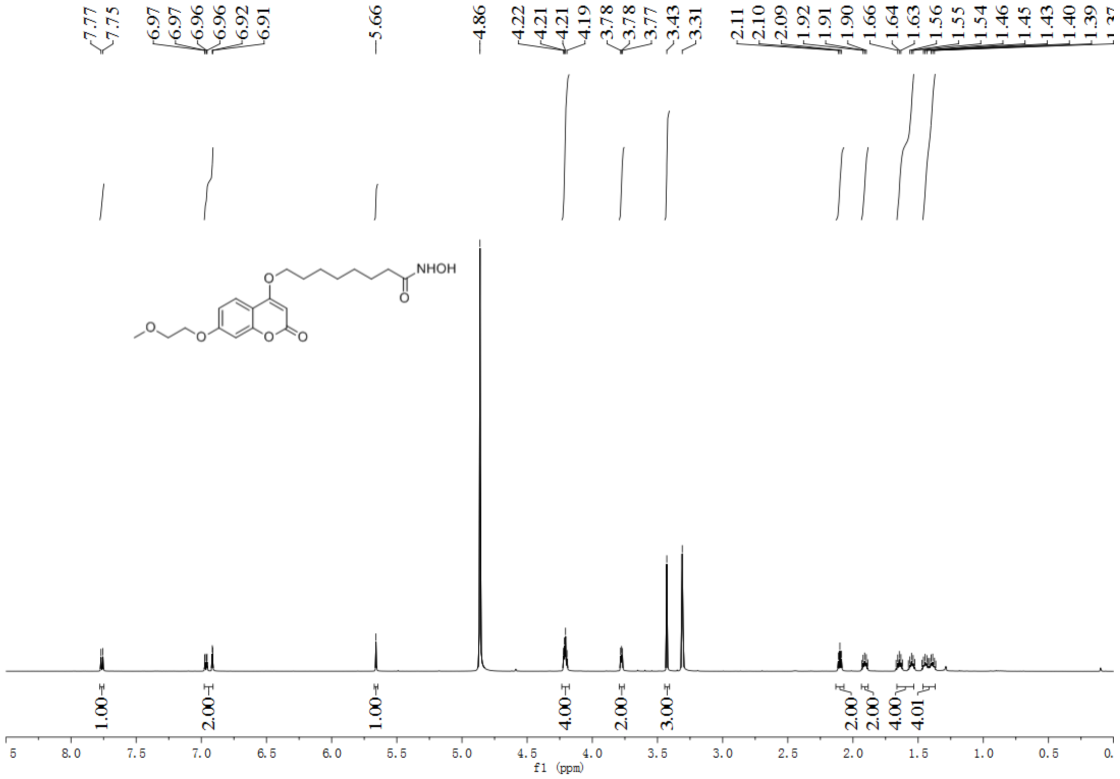

Fig.9. The ^{1}H NMR spectrum for **13c**

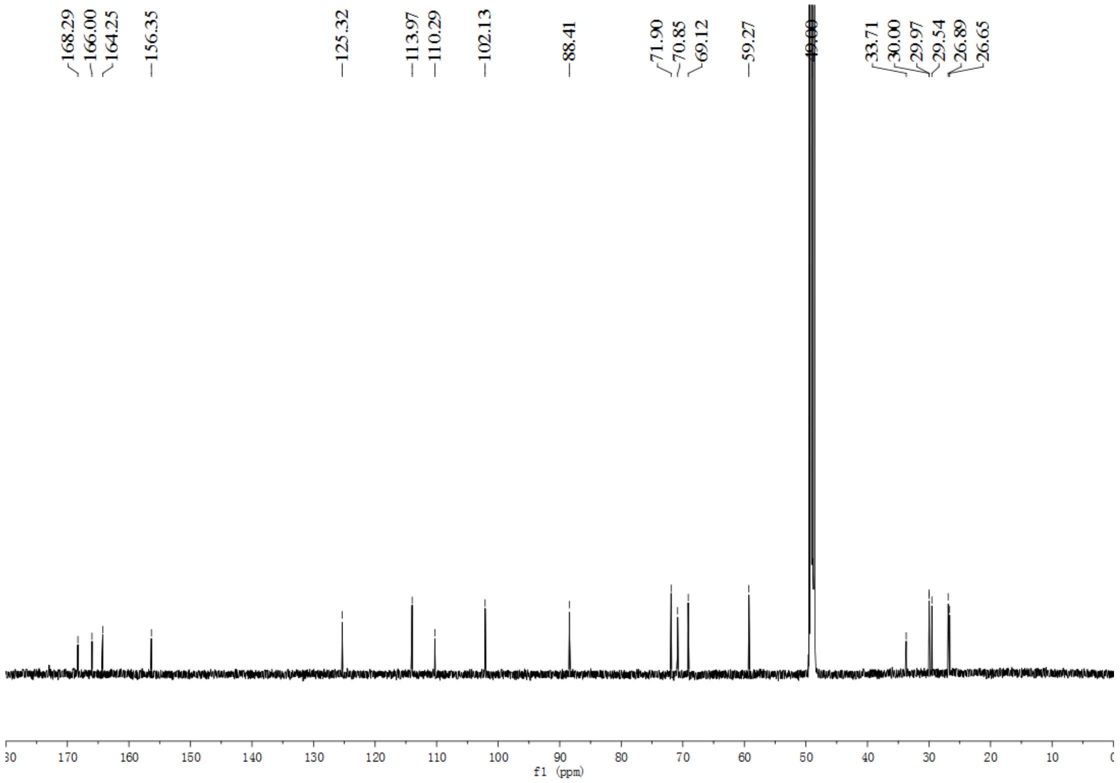

Fig.10. The ^{13}C NMR spectrum for **13c**

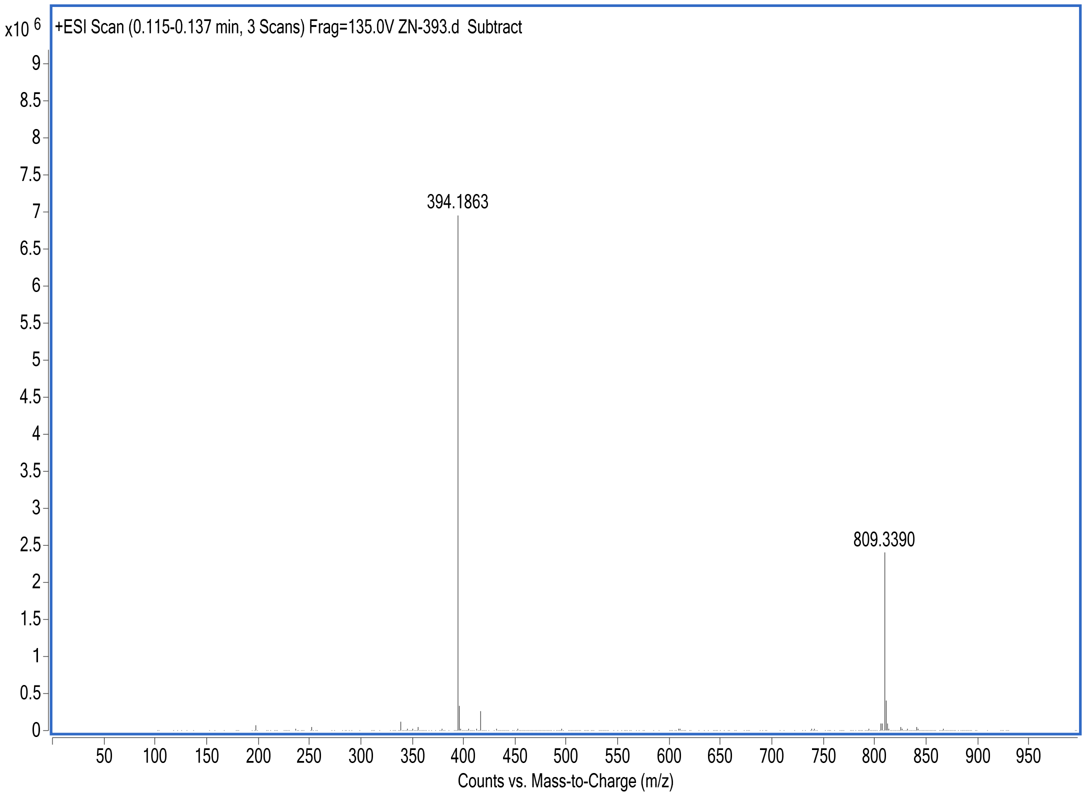

Fig.11. The HR MS spectrum for **13c**

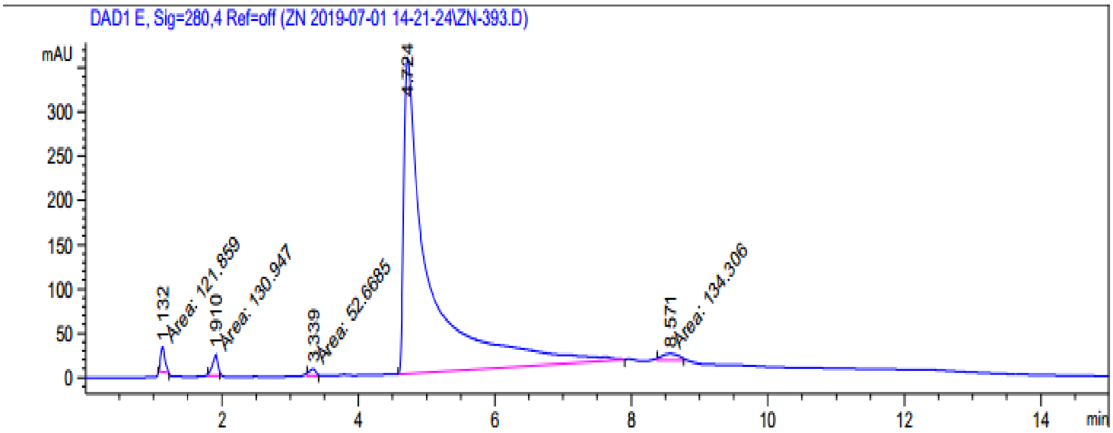

Signal 2: DAD1 E, Sig=280,4 Ref=off

Peak #	RetTime [min]	Type	Width [min]	Area [mAU*s]	Height [mAU]	Area %
1	1.132	MM	0.0714	121.85931	28.45122	1.2666
2	1.910	MM	0.0939	130.94739	23.25252	1.3611
3	3.339	MM	0.1100	52.66855	7.98057	0.5474
4	4.724	BV R	0.3368	9180.92676	356.14371	95.4288
5	8.571	MM	0.2899	134.30647	7.72207	1.3960

Fig.12. The HPLC for **13c**

Compound 13d

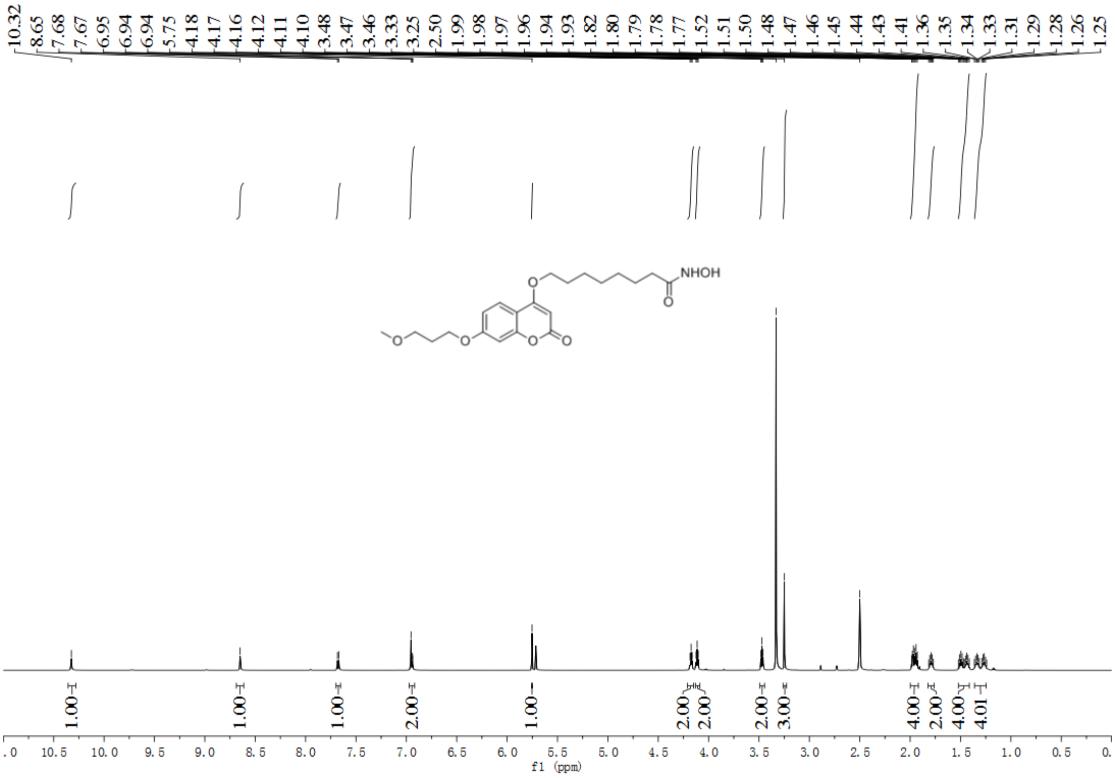

Fig.13. The ^{1}H NMR spectrum for **13d**

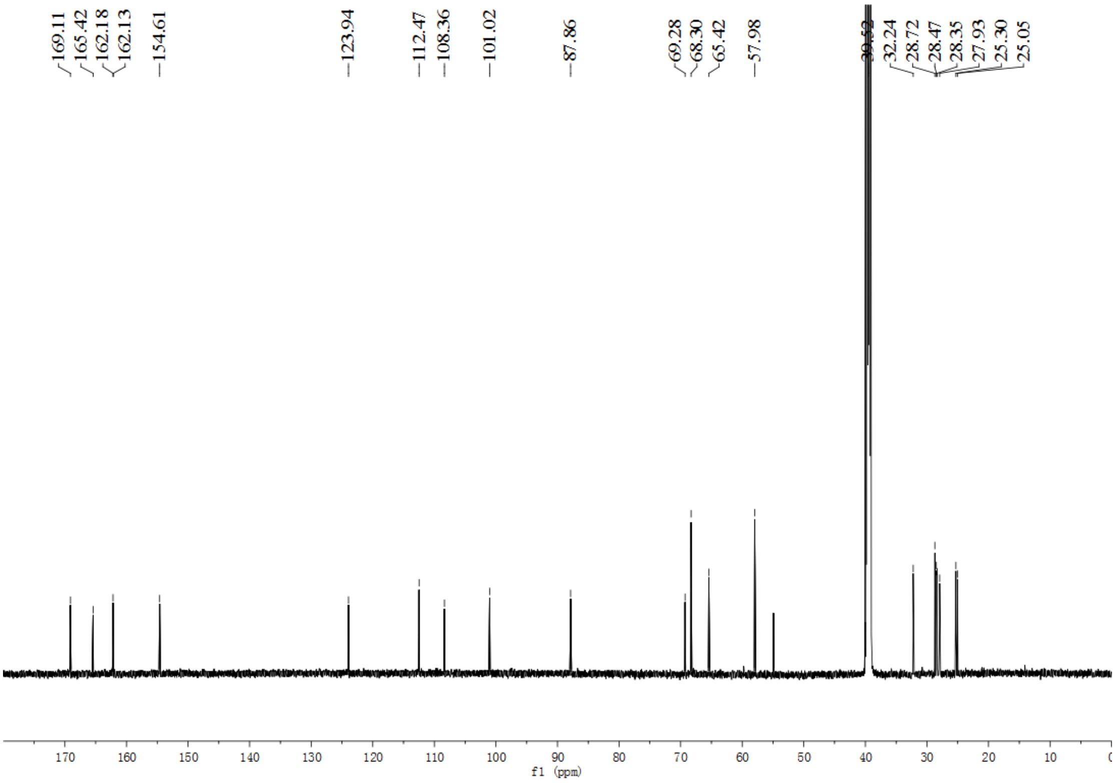

Fig.14. The ^{13}C NMR spectrum for **13d**

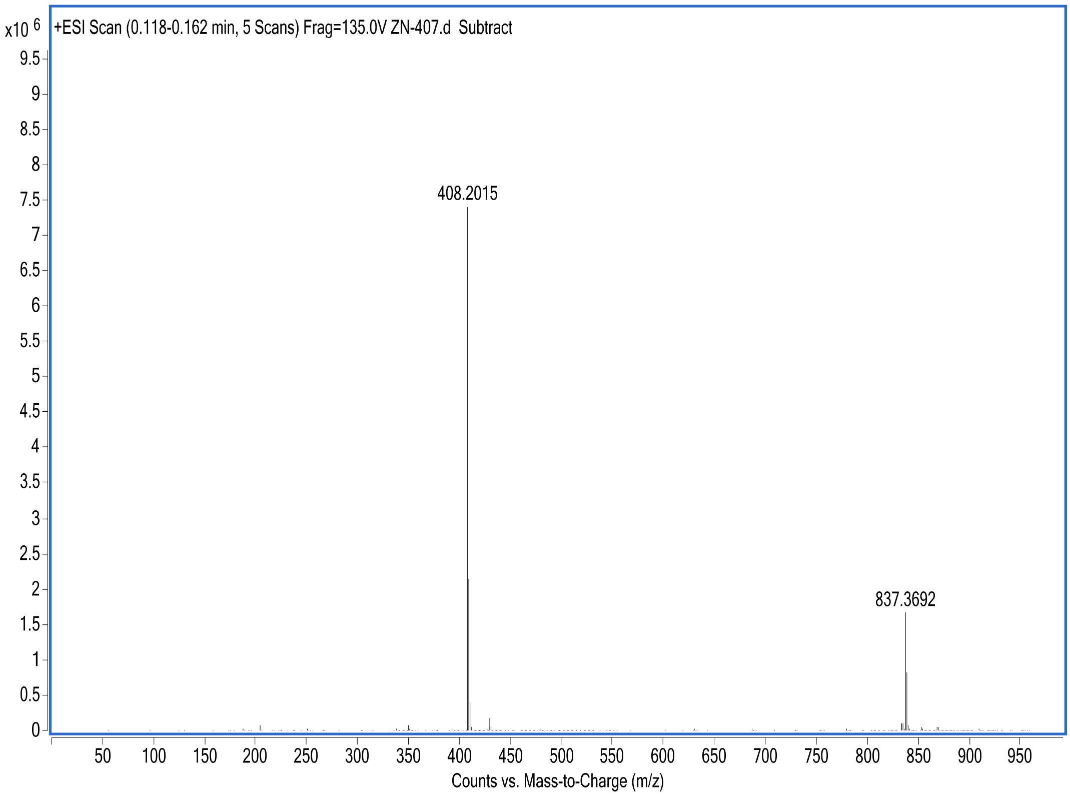

Fig.15. The HR MS spectrum for **13d**

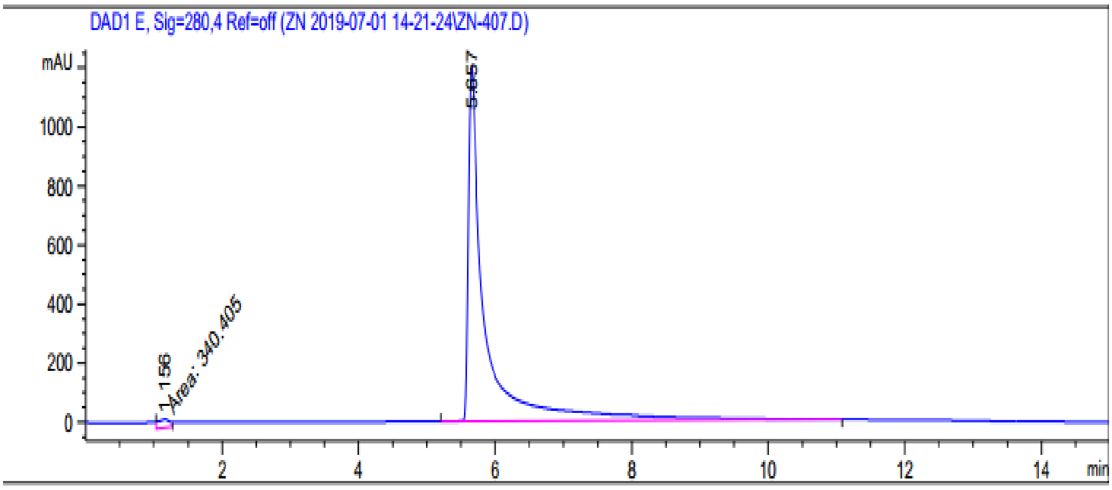

Signal 2: DAD1 E, Sig=280,4 Ref=off

Peak #	RetTime [min]	Type	Width [min]	Area [mAU*s]	Height [mAU]	Area %
1	1.156	MM	0.1993	340.40472	28.46984	1.6364
2	5.657	BV R	0.2278	2.04612e4	1199.98840	98.3636

Fig.16. The HPLC for **13d**

Compound 13e

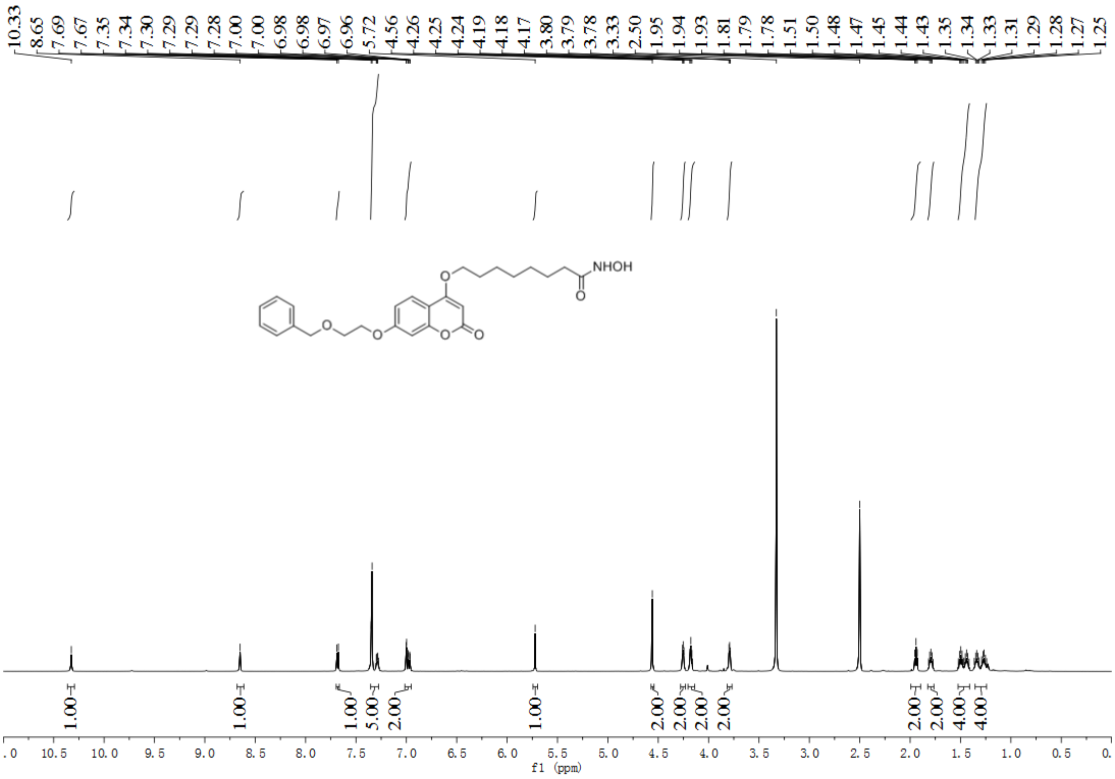

Fig.17. The ^{1}H NMR spectrum for **13e**

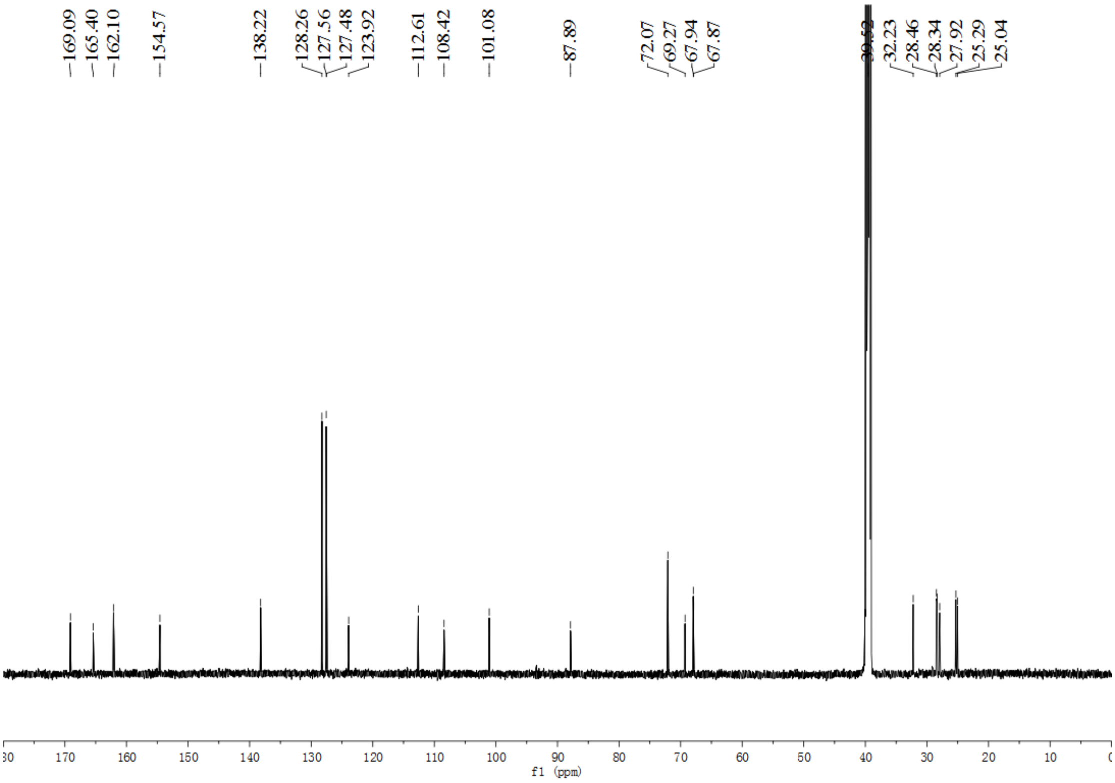

Fig.18. The ^{13}C NMR spectrum for **13e**

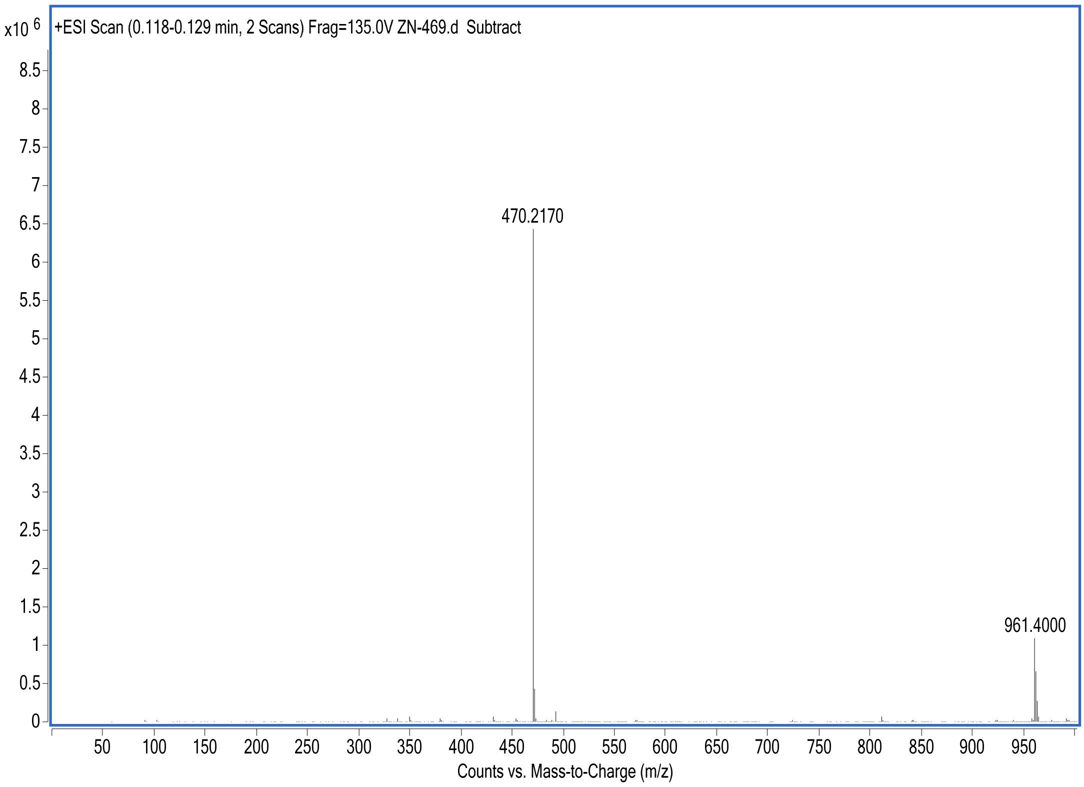

Fig. 19. The HR MS spectrum for **13e**

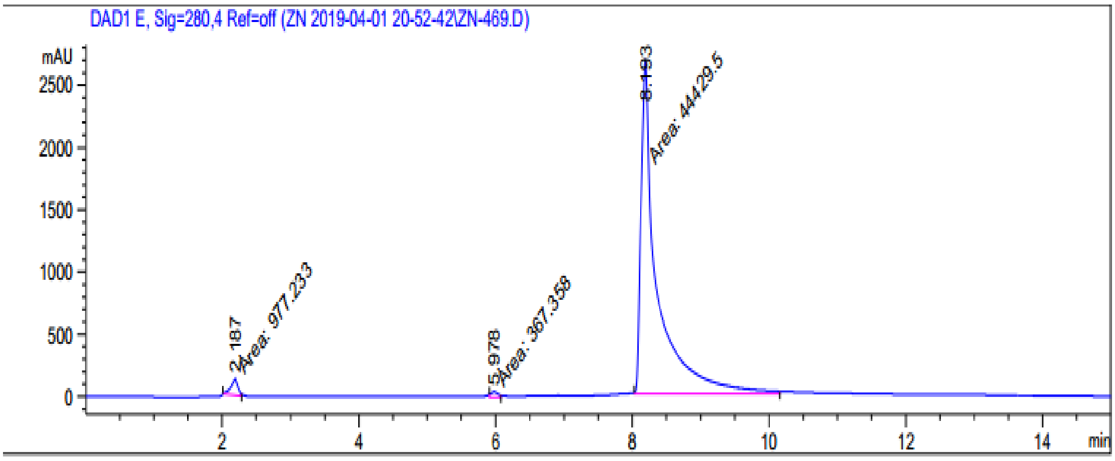

Signal 2: DAD1 E, Sig=280,4 Ref=off

Peak #	RetTime [min]	Type	Width [min]	Area [mAU*s]	Height [mAU]	Area %
1	2.187	MM	0.1267	977.23322	128.55794	2.1349
2	5.978	MM	0.1327	367.35809	46.14465	0.8025
3	8.193	MM	0.2768	4.44295e4	2675.22461	97.0625

Fig. 20. The HPLC for **13e**

Compound 13f

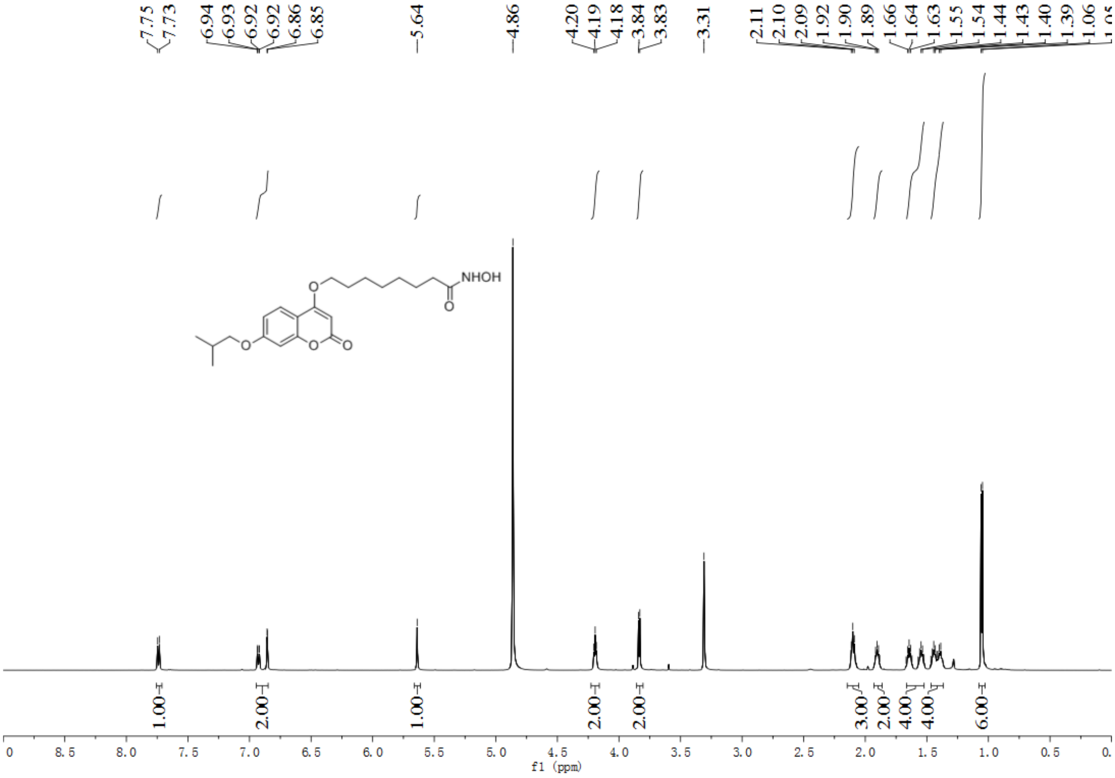

Fig.21. The ^{1}H NMR spectrum for **13f**

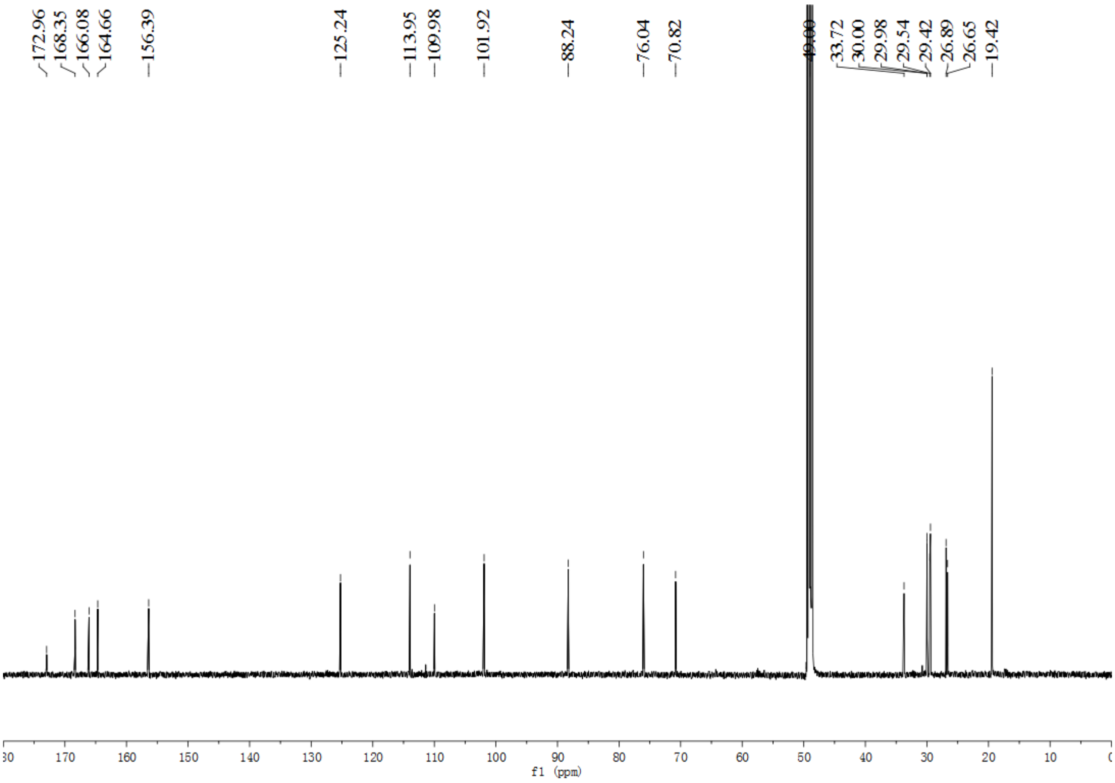

Fig.22. The ^{13}C NMR spectrum for **13f**

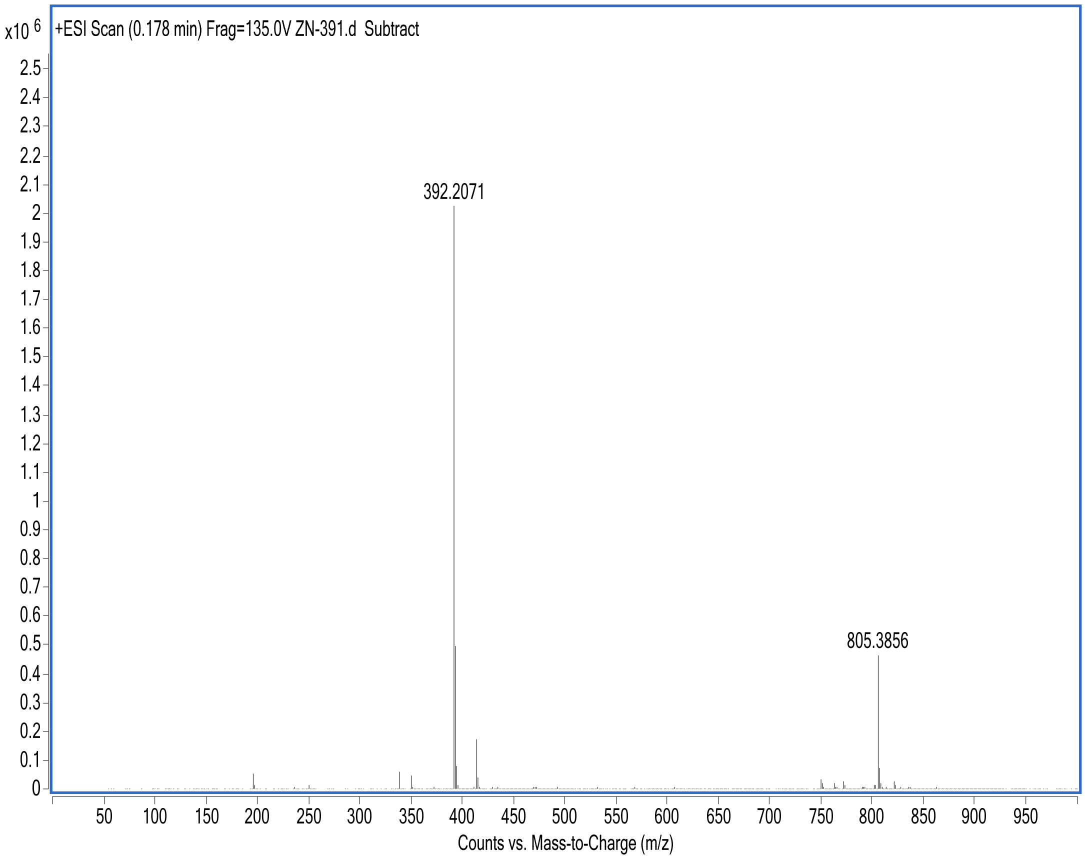

Fig.23. The HR MS spectrum for **13f**

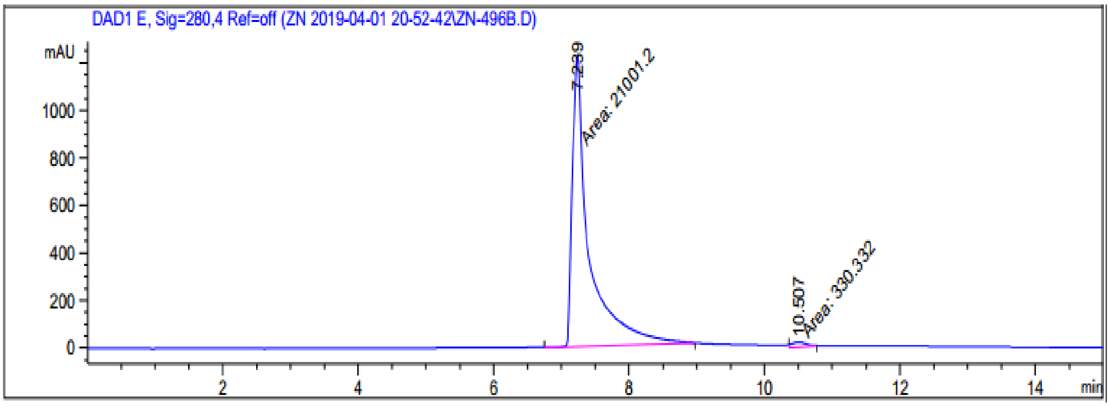

Signal 2: DAD1 E, Sig=280,4 Ref=off

Peak #	RetTime [min]	Type	Width [min]	Area [mAU*s]	Height [mAU]	Area %
1	7.239	MM	0.2857	2.10012e4	1225.19482	98.4514
2	10.507	MM	0.2509	330.33191	21.94214	1.5486

Fig.24. The HPLC for **13f**

Compound 13g

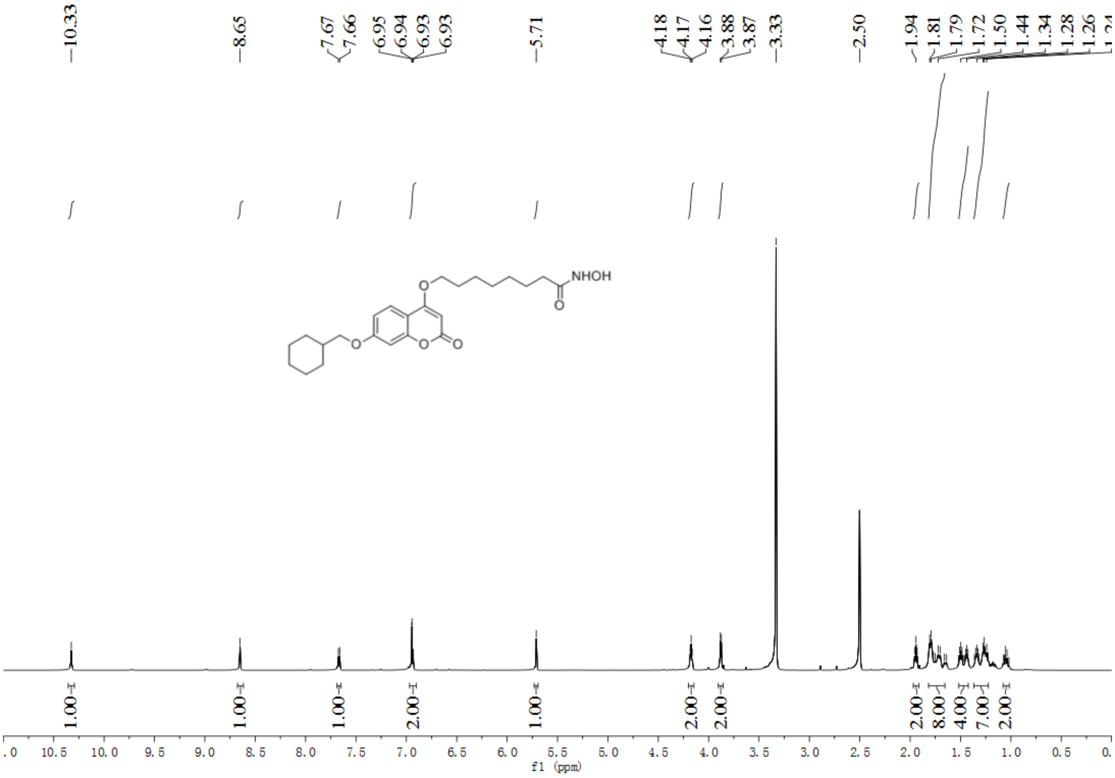

Fig.25. The ^{1}H NMR spectrum for **13g**

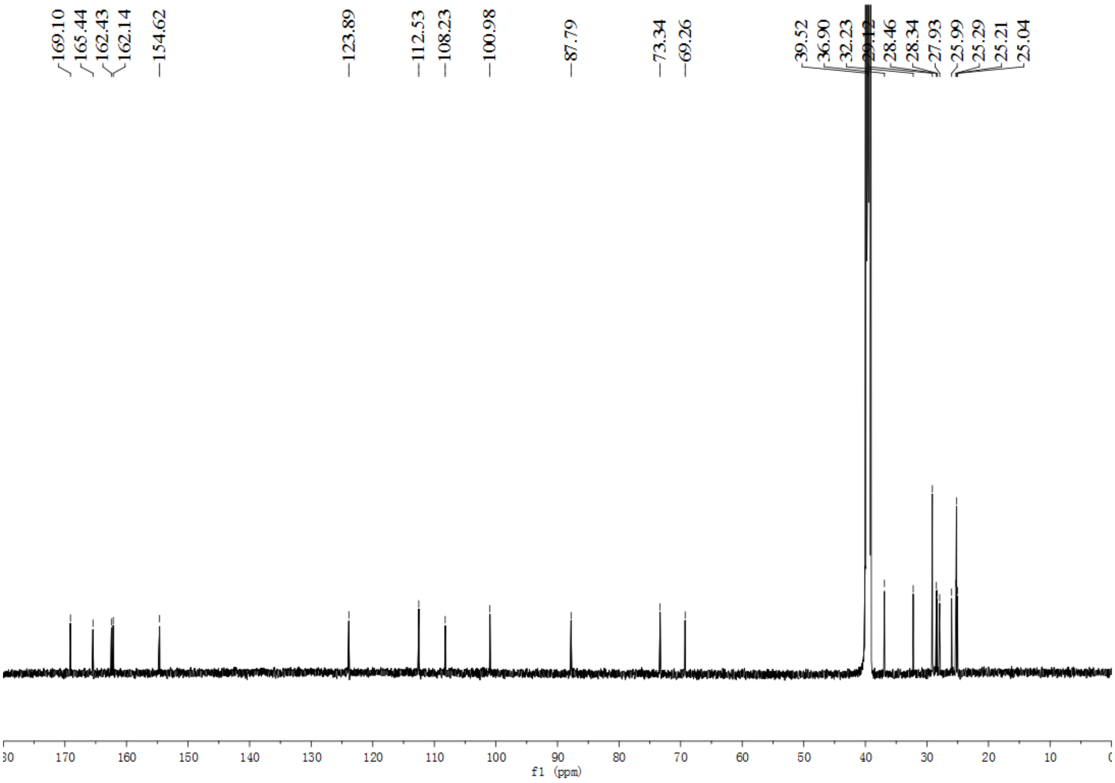

Fig.26. The ^{13}C NMR spectrum for **13g**

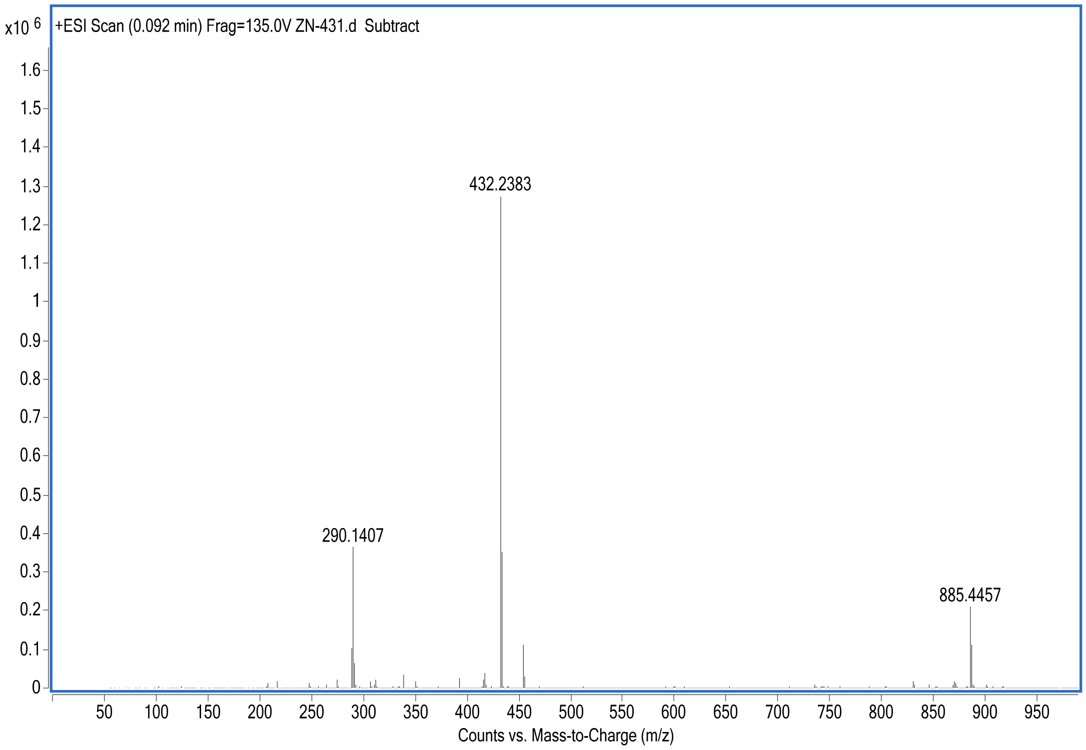

Fig.27. The HR MS spectrum for **13g**

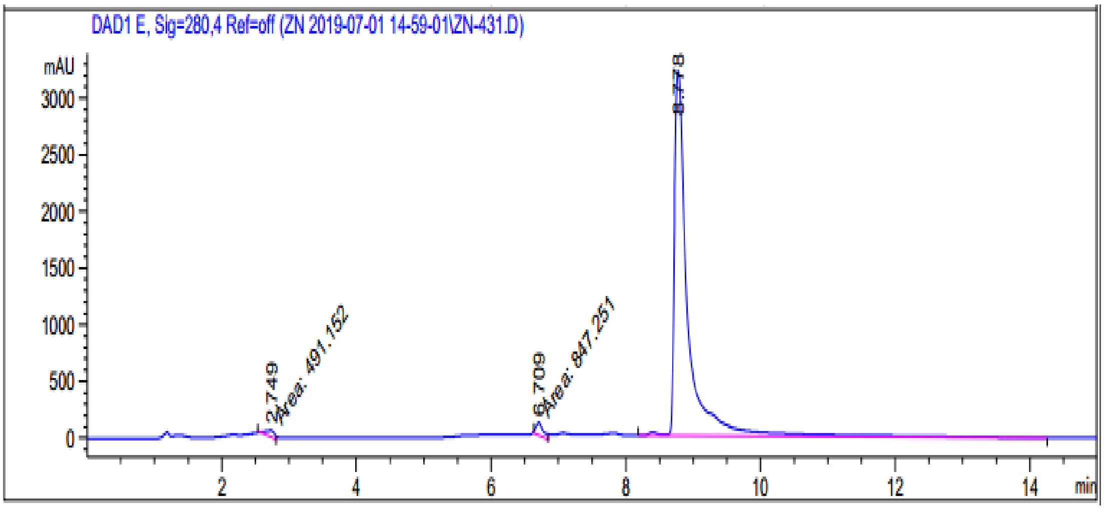

Signal 2: DAD1 E, Sig=280,4 Ref=off

Peak #	RetTime [min]	Type	Width [min]	Area [mAU*s]	Height [mAU]	Area %
1	2.749	MM	0.1407	491.15182	58.19069	1.0605
2	6.709	MM	0.1244	847.25092	113.49999	1.8295
3	8.778	VBAR	0.1980	4.49731e4	3217.69141	97.1100

Fig.28. The HPLC for **13g**

Compound 14a

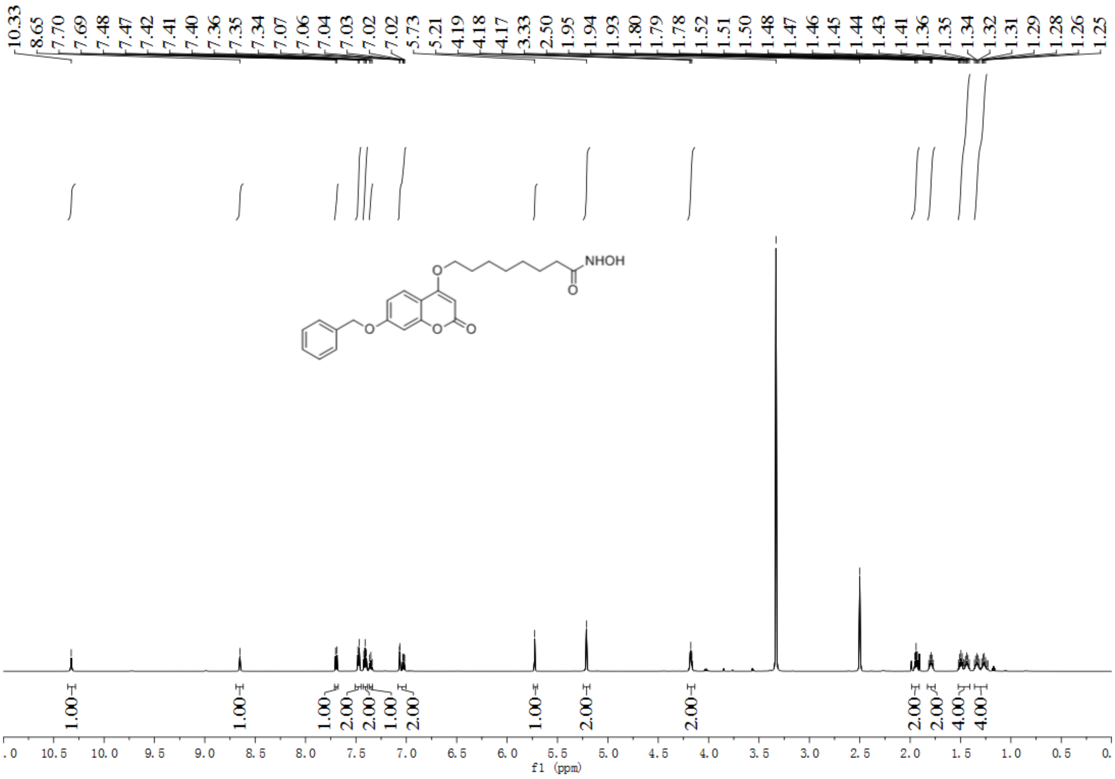

Fig.29. The ^{1}H NMR spectrum for **14a**

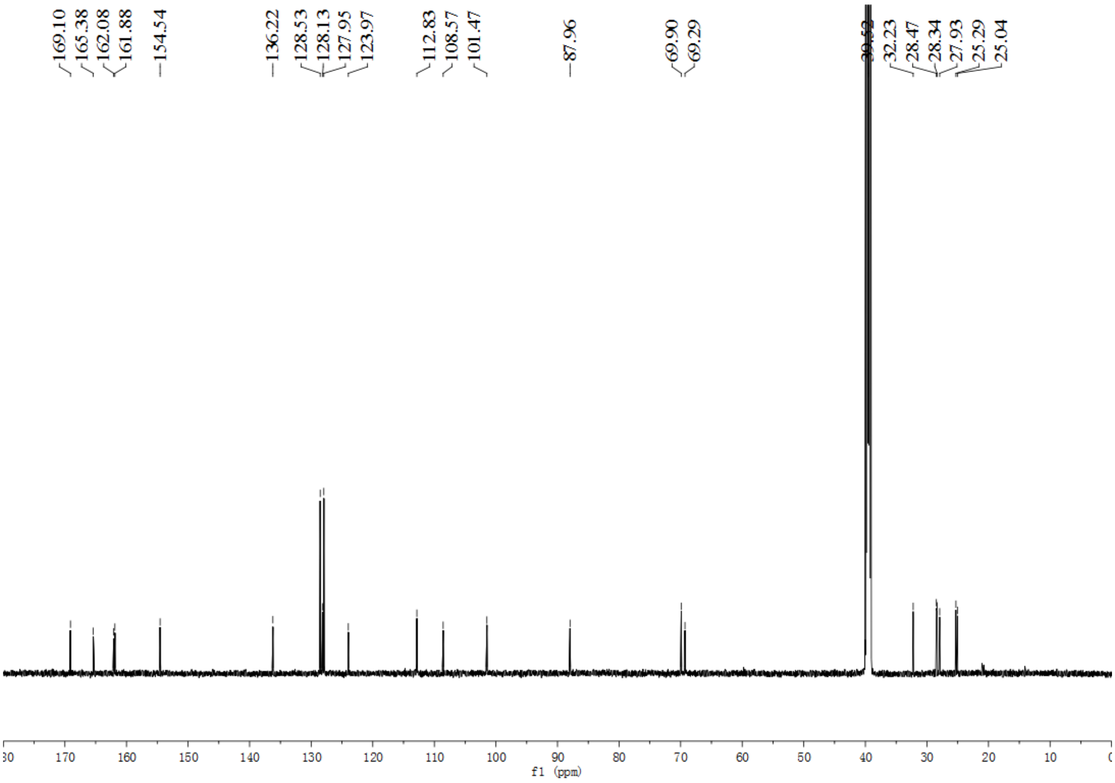

Fig.30. The ^{13}C NMR spectrum for **14a**

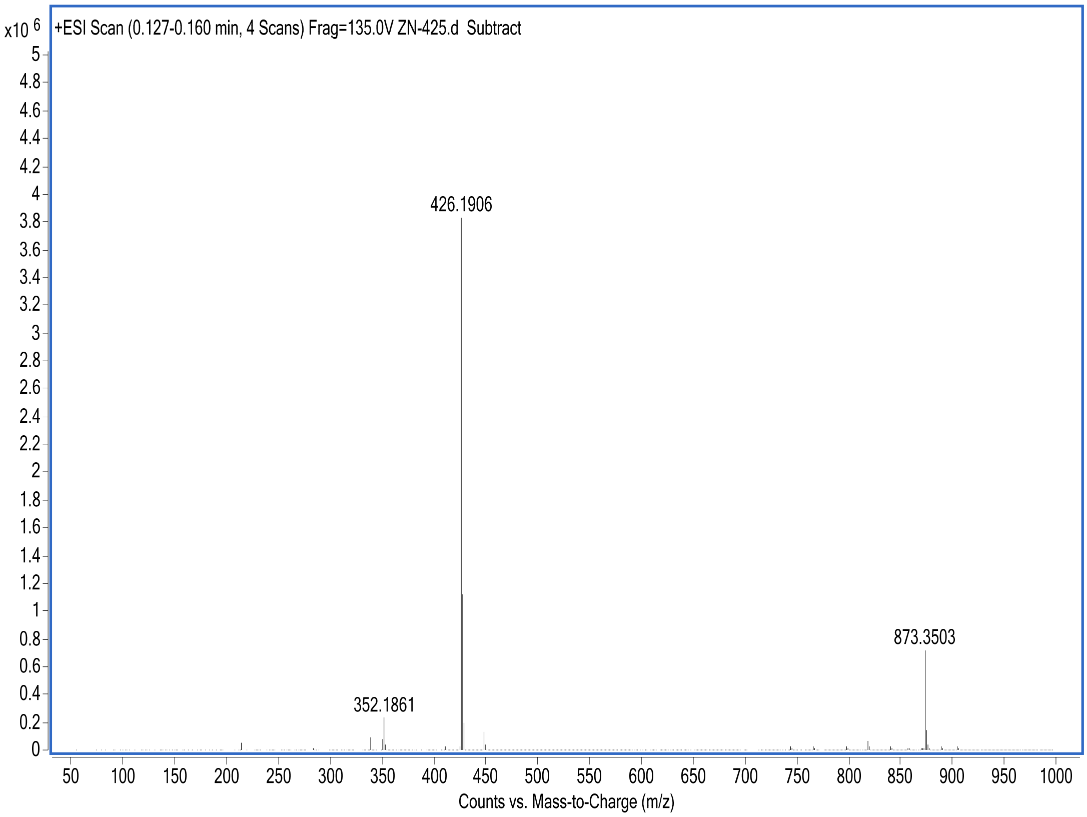

Fig.31. The HR MS spectrum for **14a**

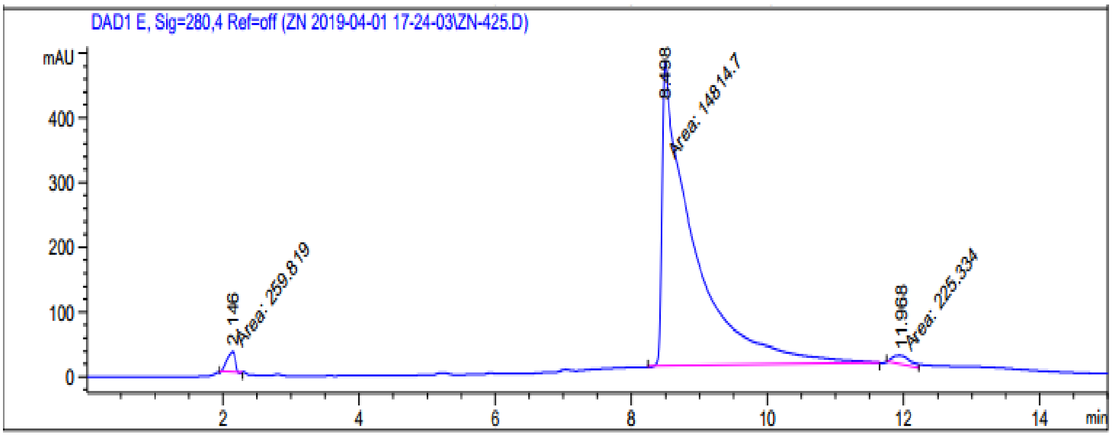

Peak #	RetTime [min]	Type	Width [min]	Area [mAU*s]	Height [mAU]	Area %
1	2.146	MM	0.1355	259.81851	31.94764	1.6982
2	8.498	MM	0.5254	1.48147e4	469.98801	96.8290
3	11.968	MM	0.2932	225.33365	12.80848	1.4728

Fig.32. The HPLC for **14a**

Compound 14b

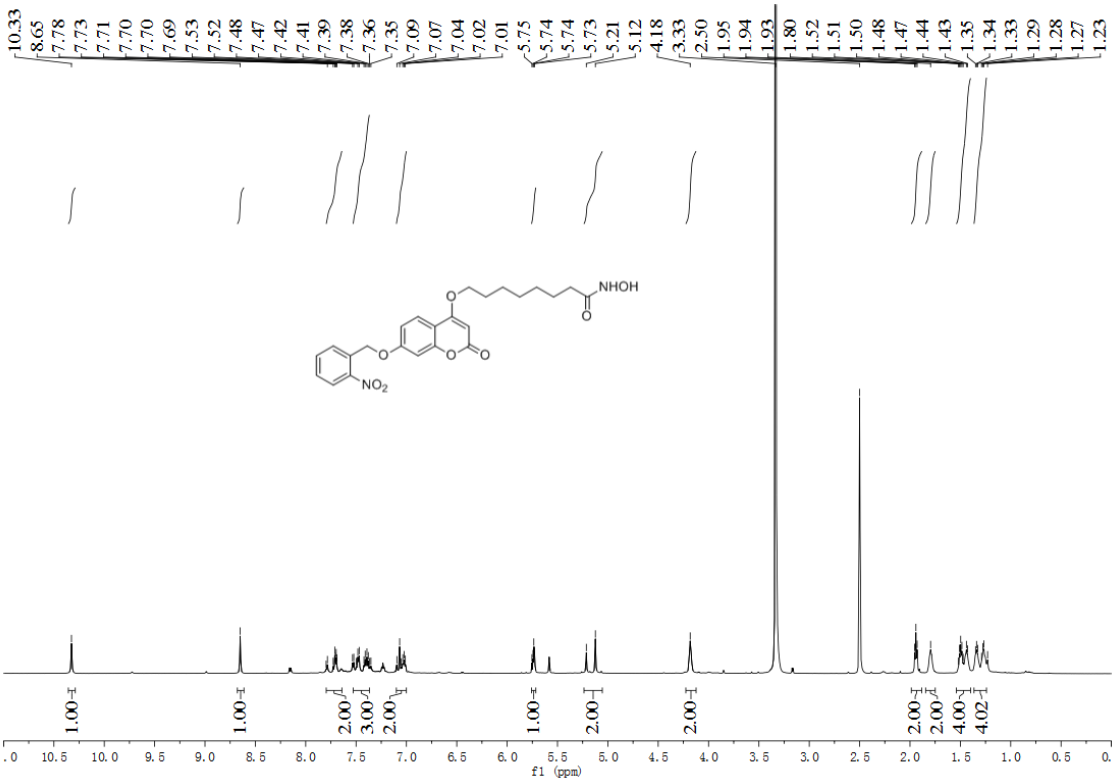

Fig.33. The ^{1}H NMR spectrum for **14b**

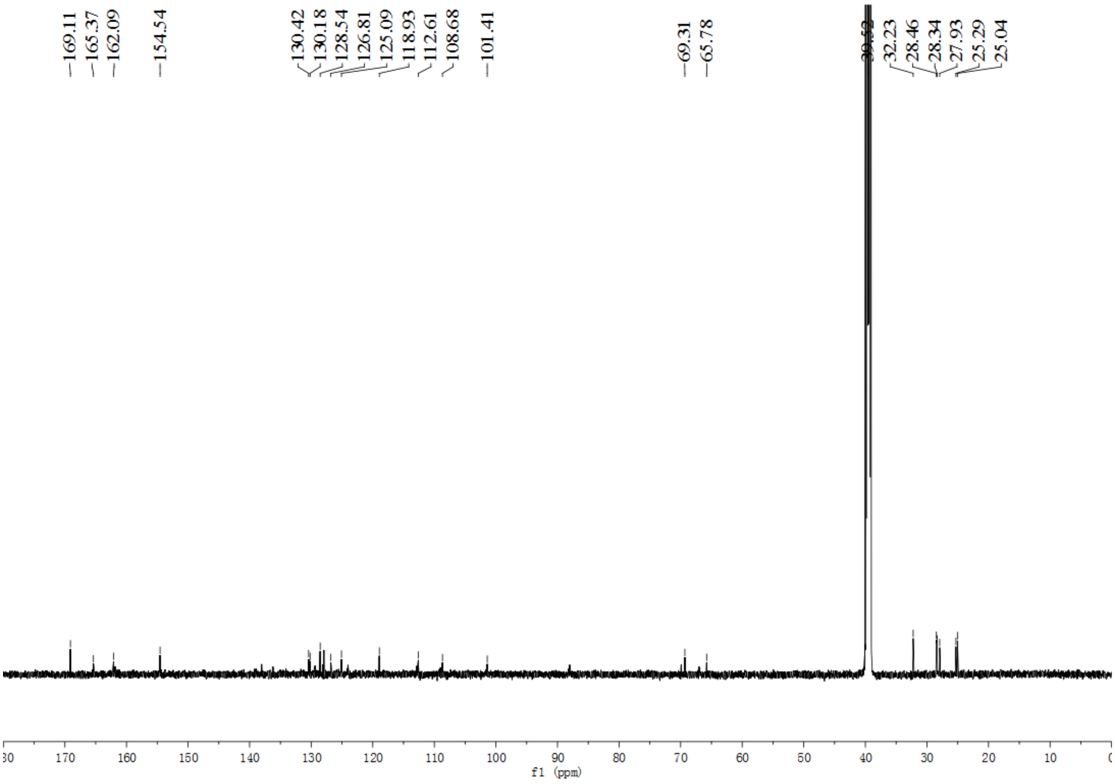

Fig.34. The ^{13}C NMR spectrum for **14b**

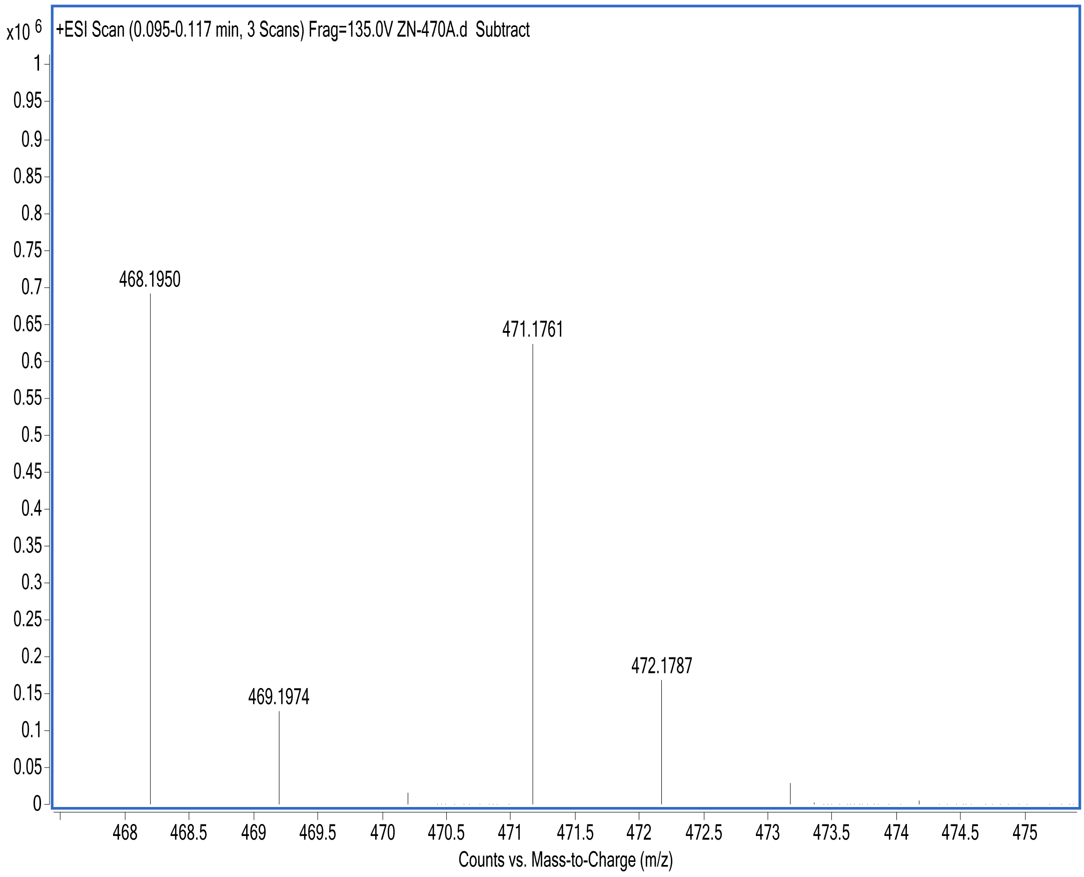

Fig.35. The HR MS spectrum for **14b**

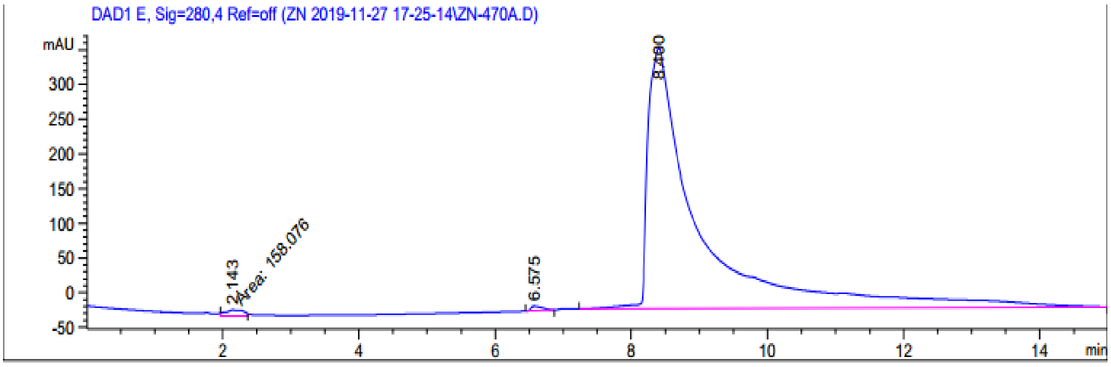

Peak #	RetTime [min]	Type	Width [min]	Area [mAU*s]	Height [mAU]	Area %
1	2.143	MM	0.3079	158.07550	8.55581	0.8008
2	6.575	BB	0.1929	75.35004	6.61317	0.3817
3	8.400	BV R	0.6631	1.95053e4	375.55200	98.8174

Fig.36. The HPLC for **14b**

Compound 14c

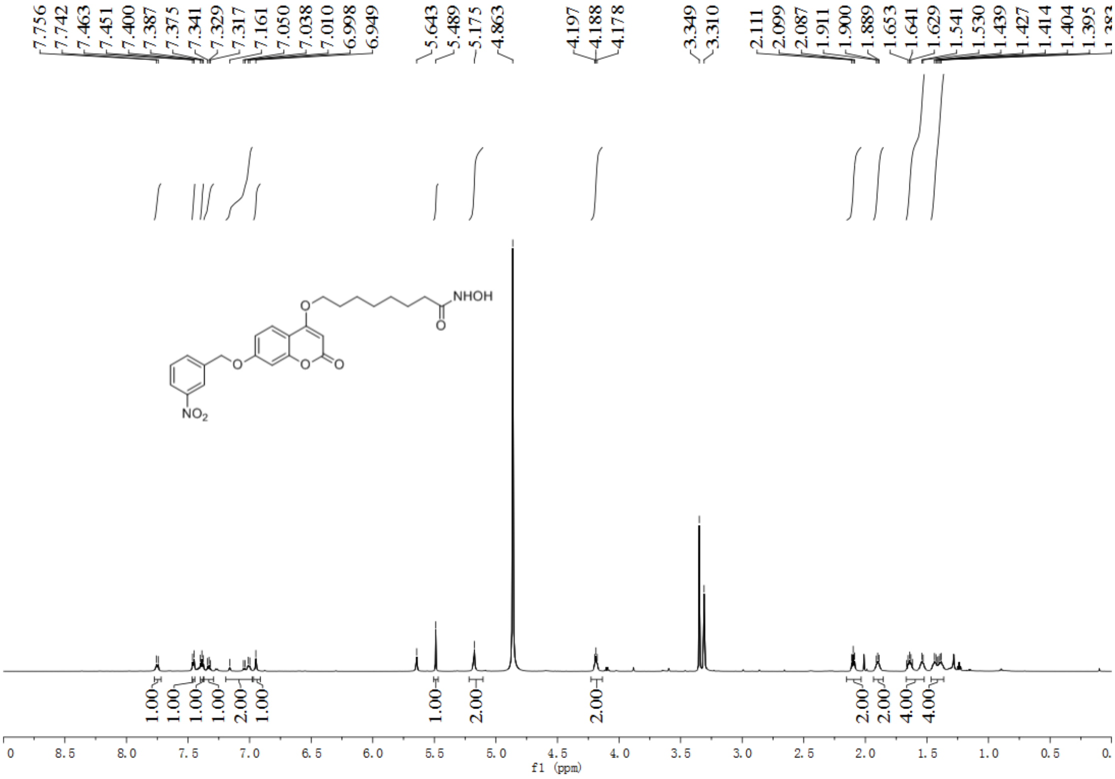

Fig.37. The ^{1}H NMR spectrum for **14c**

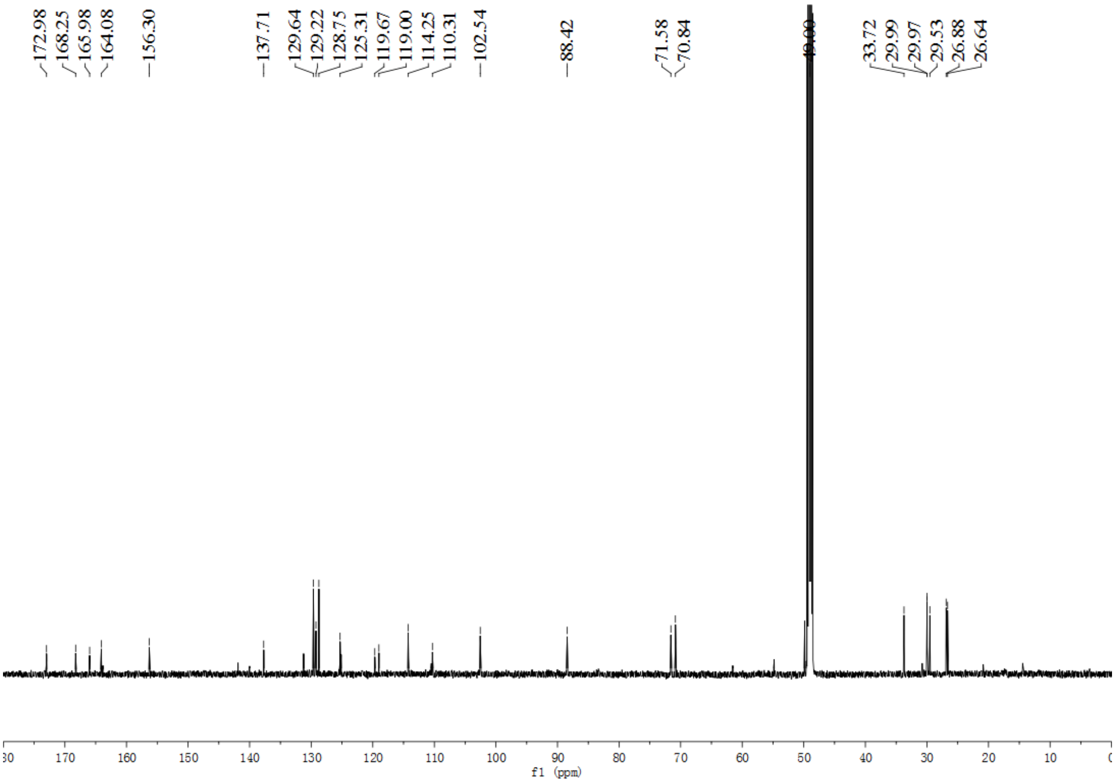

Fig.38. The ^{13}C NMR spectrum for **14c**

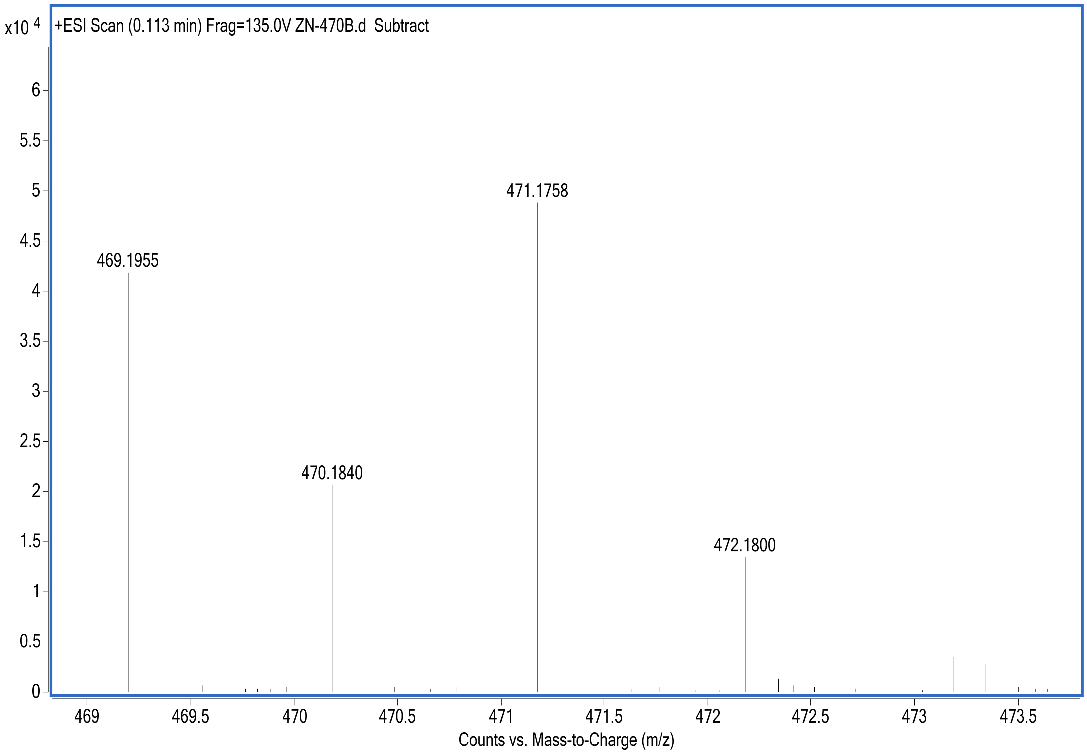

Fig.39. The HR MS spectrum for **14c**

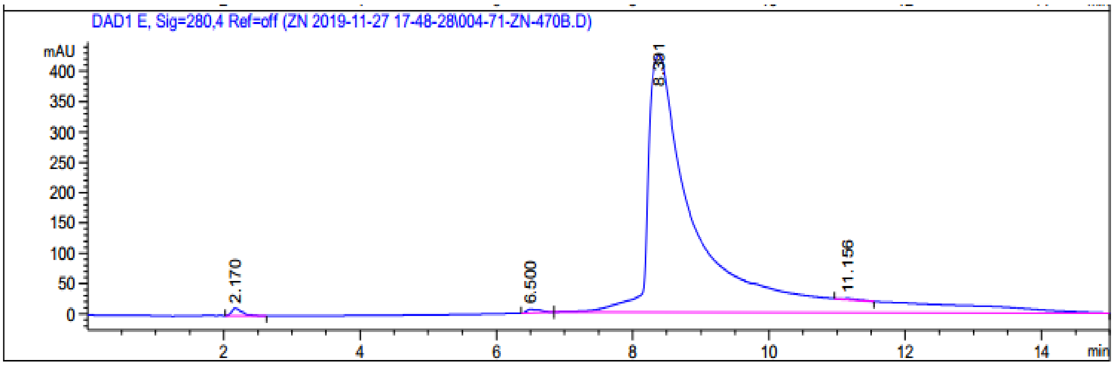

Peak #	RetTime [min]	Type	Width [min]	Area [mAU*s]	Height [mAU]	Area %
1	2.170	BV R	0.1532	133.13284	12.60868	0.5879
2	6.500	BB	0.1762	75.45833	5.78453	0.3332
3	8.391	BV R	0.7093	2.24039e4	424.78693	98.9405
4	11.156	VBAE	0.2309	31.31526	1.87463	0.1383

Fig.40. The HPLC for **14c**

Compound 14d

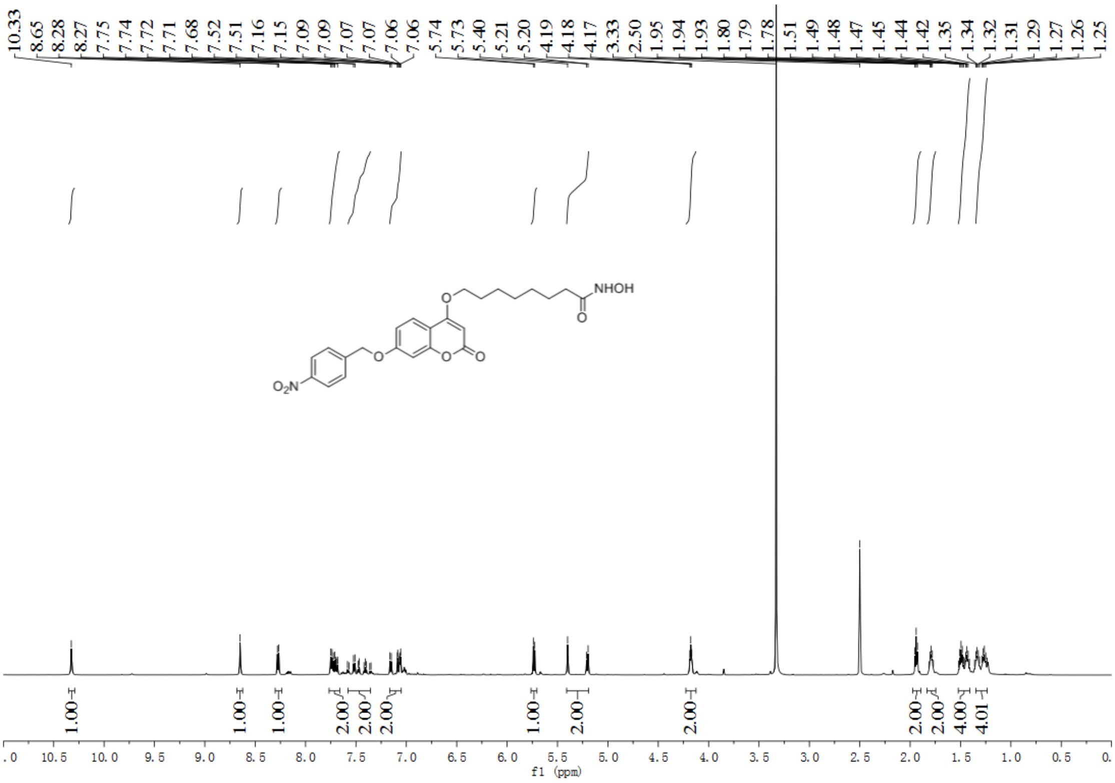

Fig.41. The ^{1}H NMR spectrum for **14d**

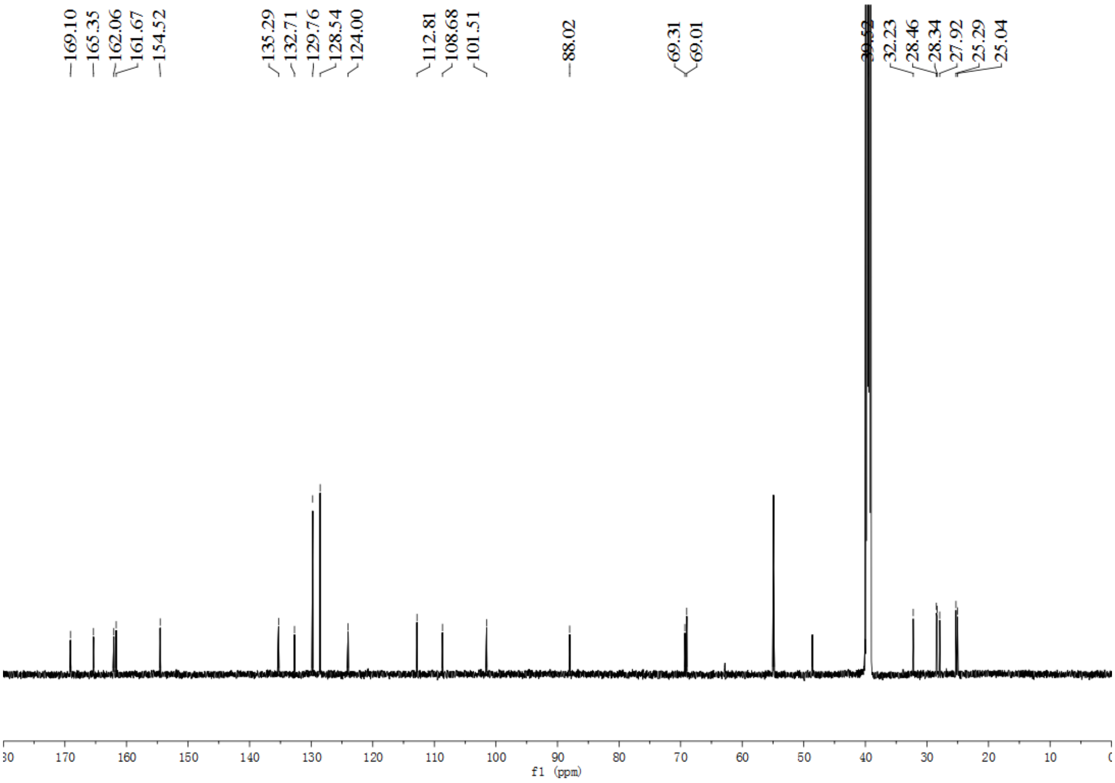

Fig.42. The ^{13}C NMR spectrum for **14d**

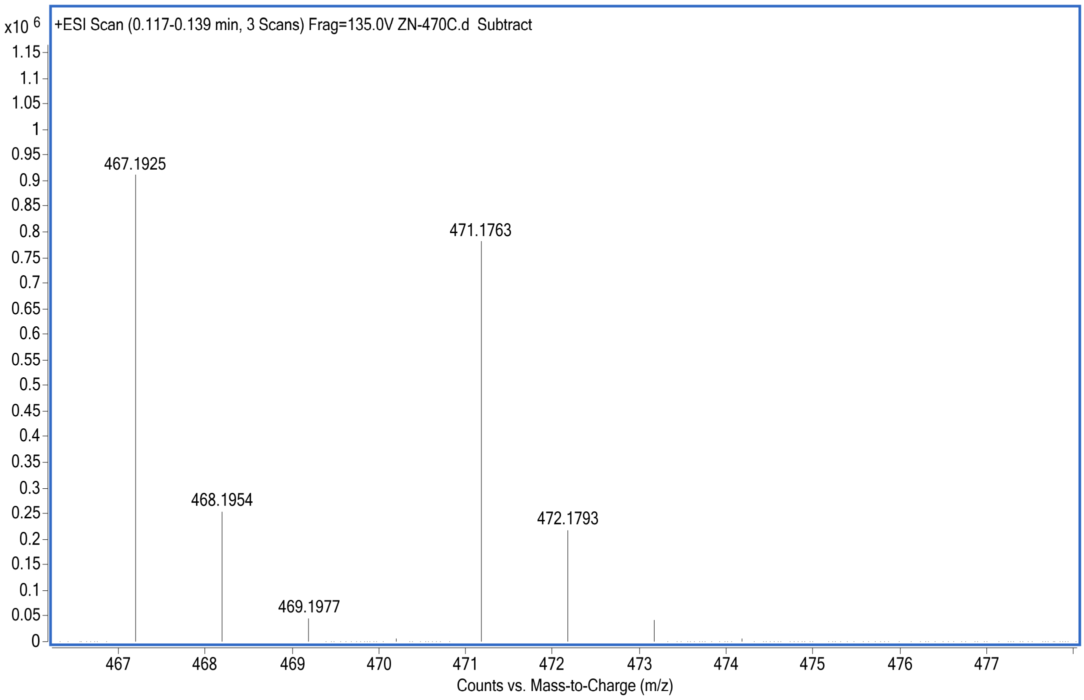

Fig.43. The HR MS spectrum for **14d**

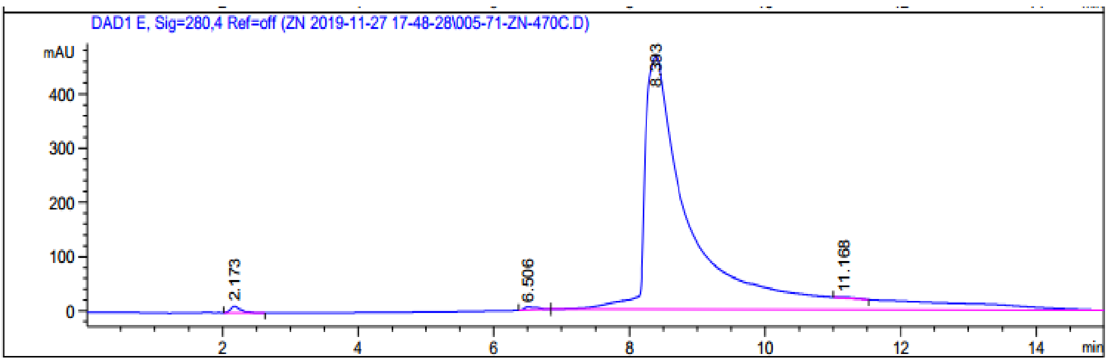

Peak #	RetTime [min]	Type	Width [min]	Area [mAU*s]	Height [mAU]	Area %
1	2.173	BB	0.1578	132.51038	12.09318	0.5577
2	6.506	BB	0.1756	75.22433	5.71474	0.3166
3	8.393	BV R	0.6791	2.35243e4	467.47079	99.0014
4	11.168	VBAE	0.2298	29.54110	1.77838	0.1243

Fig.44. The HPLC for **14d**

Compound 14e

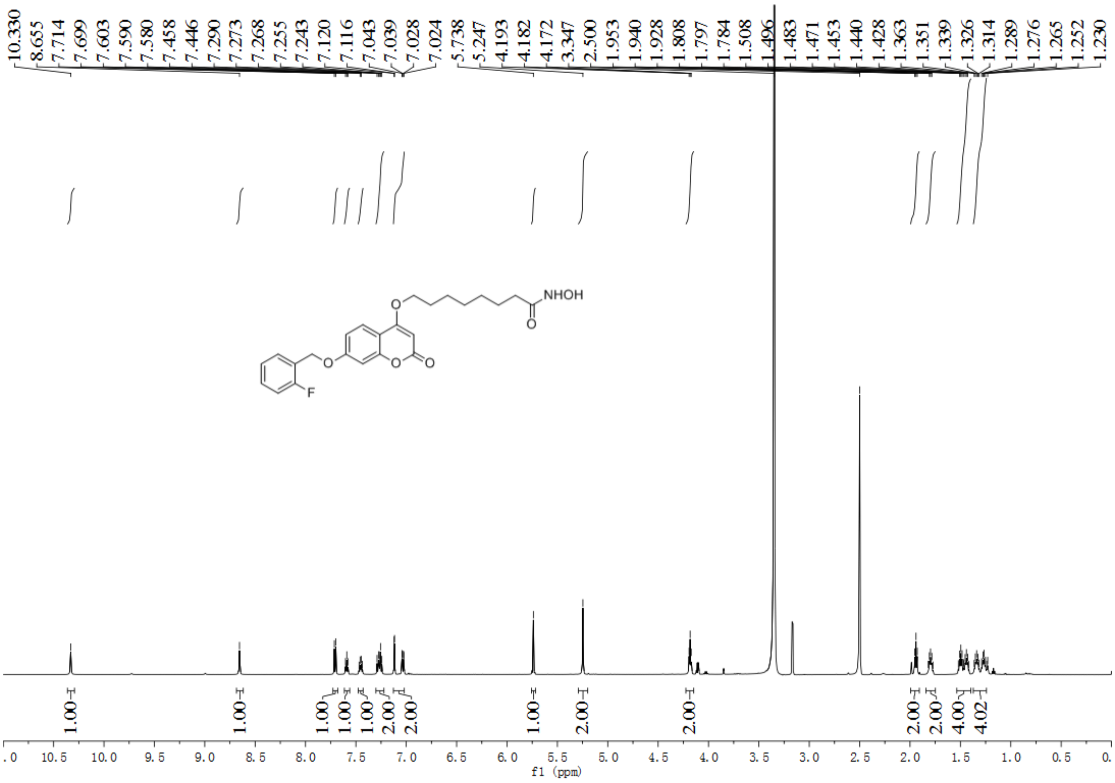

Fig.45. The ¹H NMR spectrum for **14e**

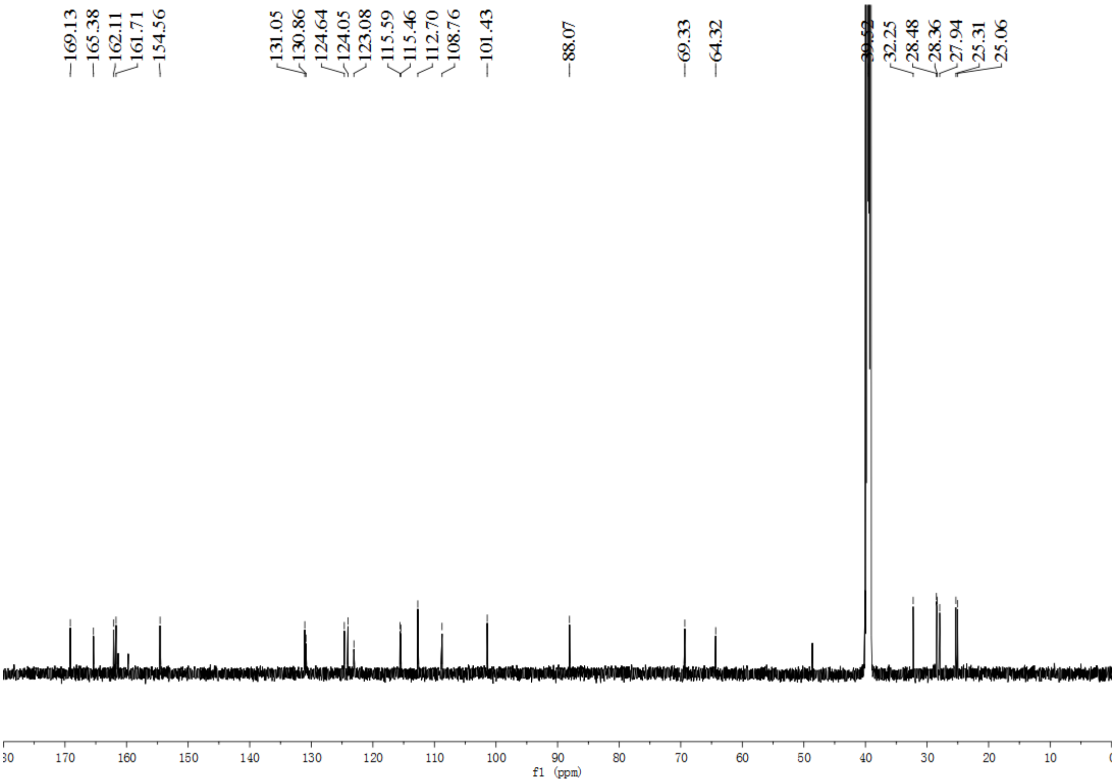

Fig.46. The ^{13}C NMR spectrum for **14e**

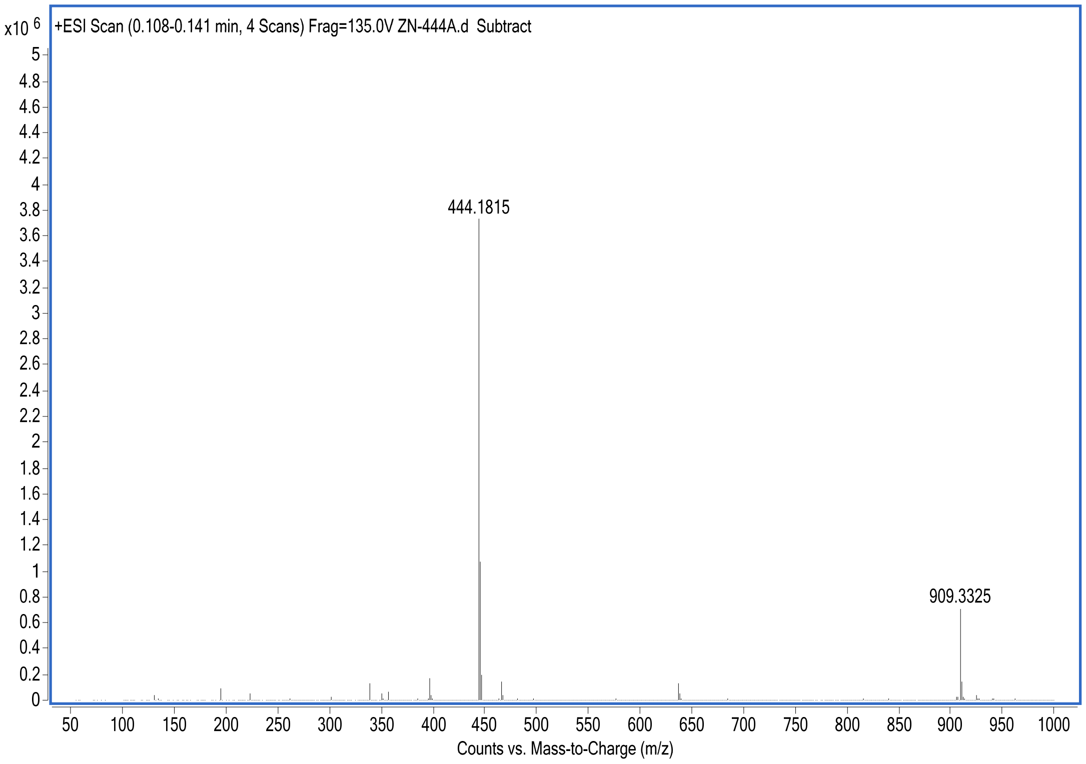

Fig.47. The HR MS spectrum for **14e**

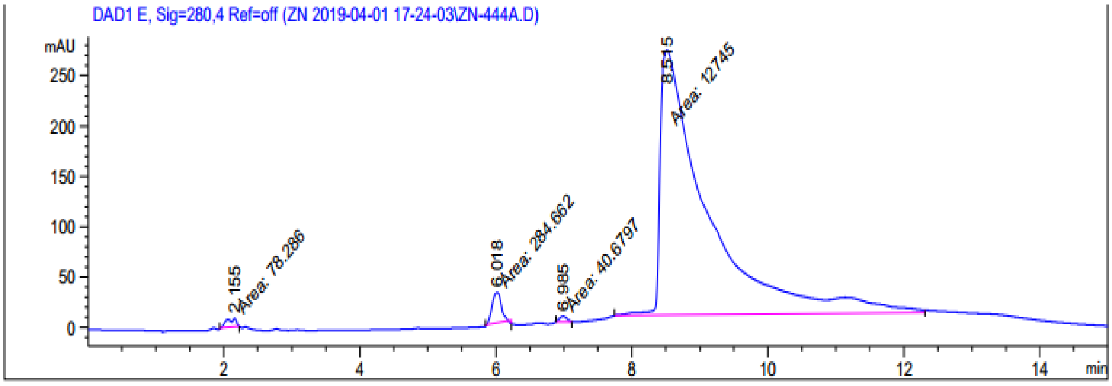

Peak #	RetTime [min]	Type	Width [min]	Area [mAU*s]	Height [mAU]	Area %
1	2.155	MM	0.1627	78.28598	8.02054	0.5954
2	6.018	MM	0.1583	284.66229	29.97689	2.1650
3	6.985	MM	0.1244	40.67971	5.44973	0.3094
4	8.515	PM	0.8068	1.27450e4	263.29767	96.9303

Fig.48. The HPLC for **14e**

Compound 14f

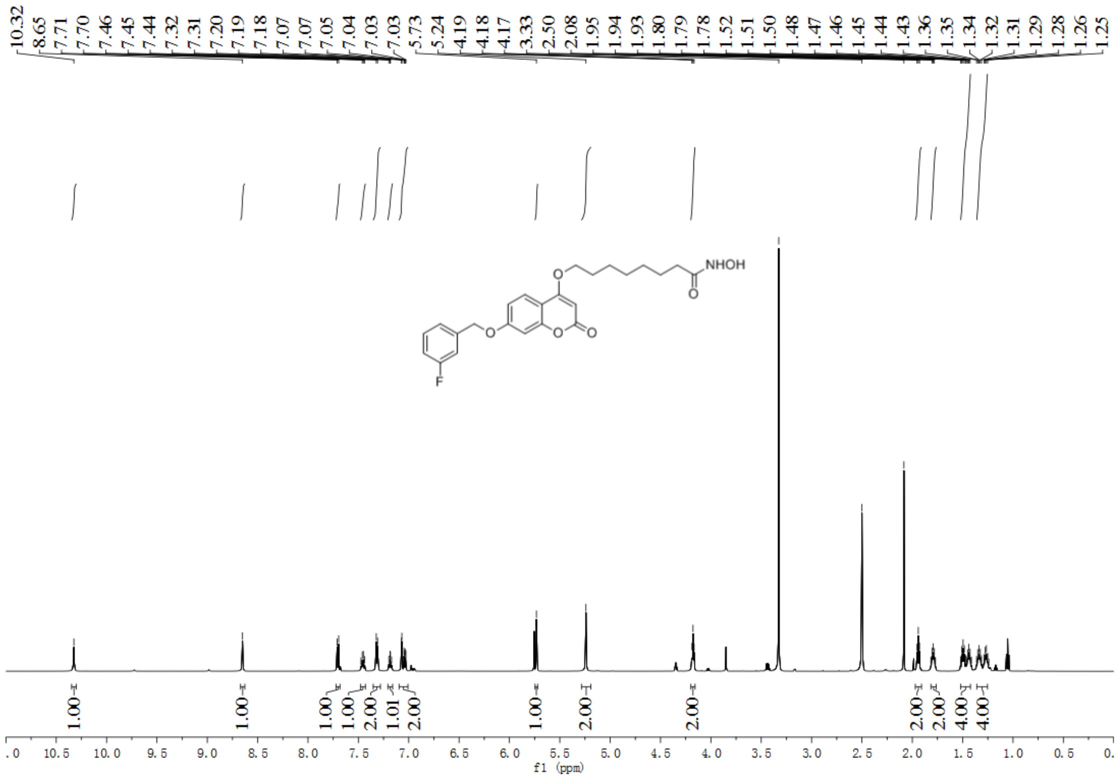

Fig.49. The ^{1}H NMR spectrum for **14f**

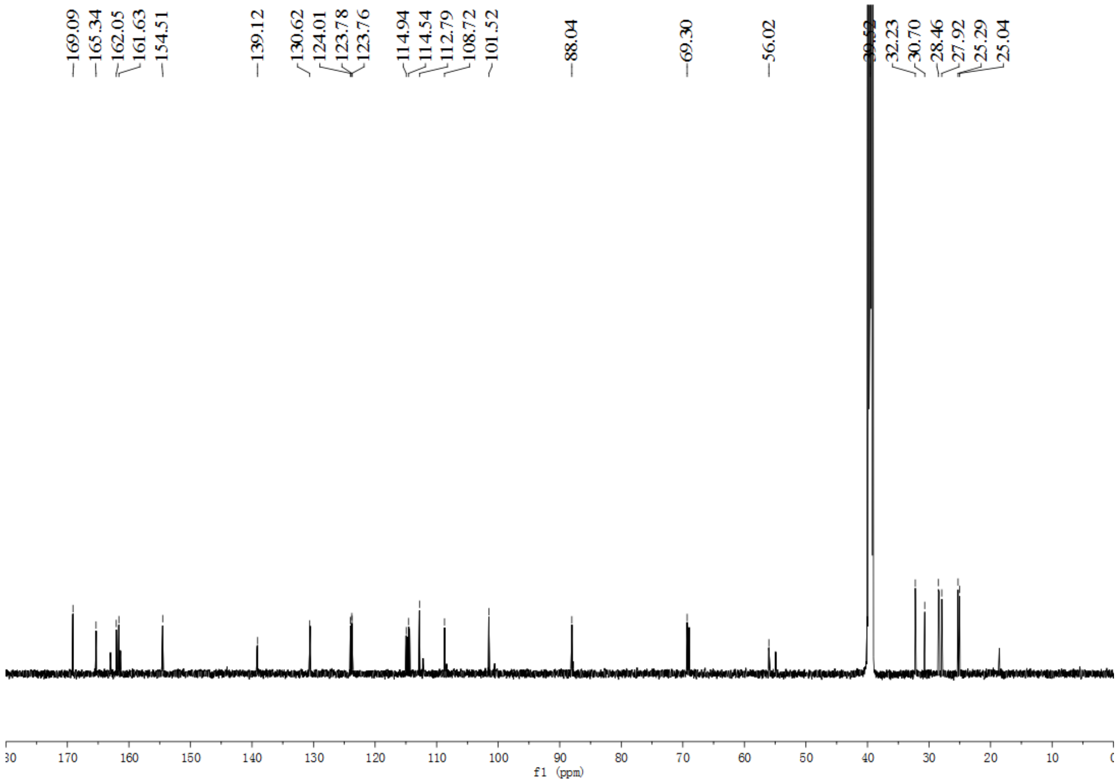

Fig.50. The ^{13}C NMR spectrum for **14f**

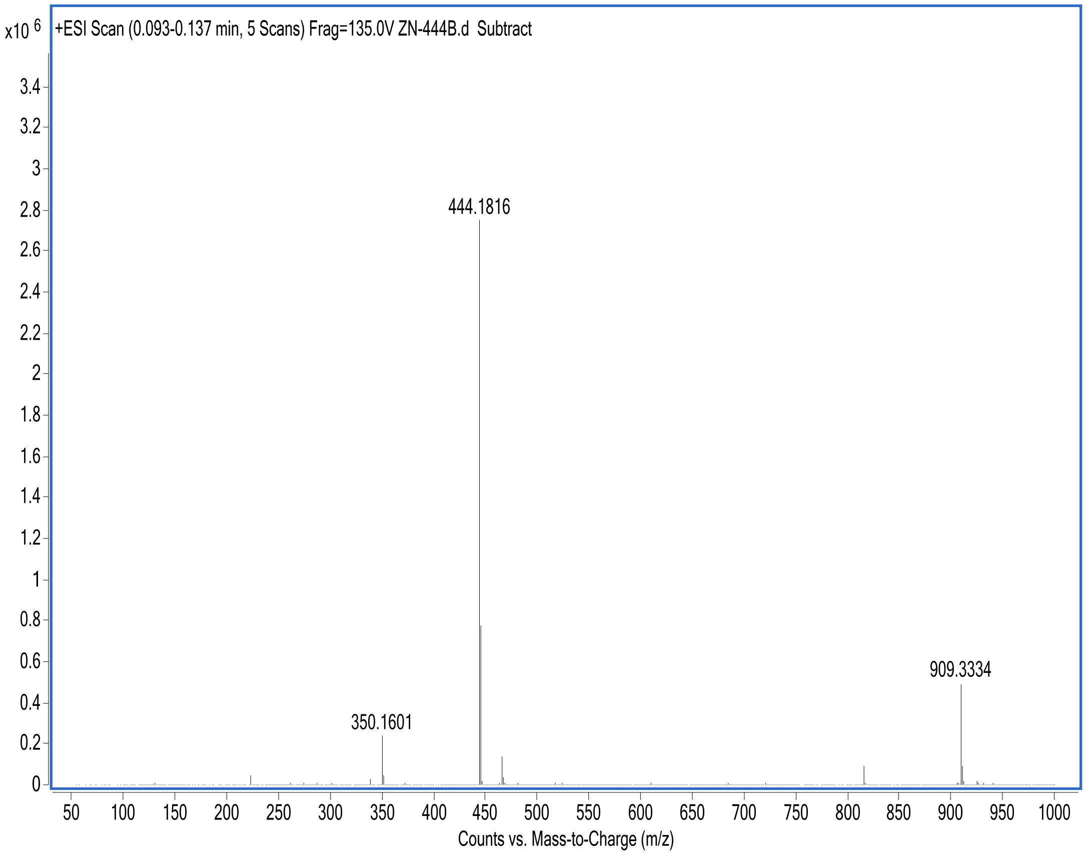

Fig.51. The HR MS spectrum for **14f**

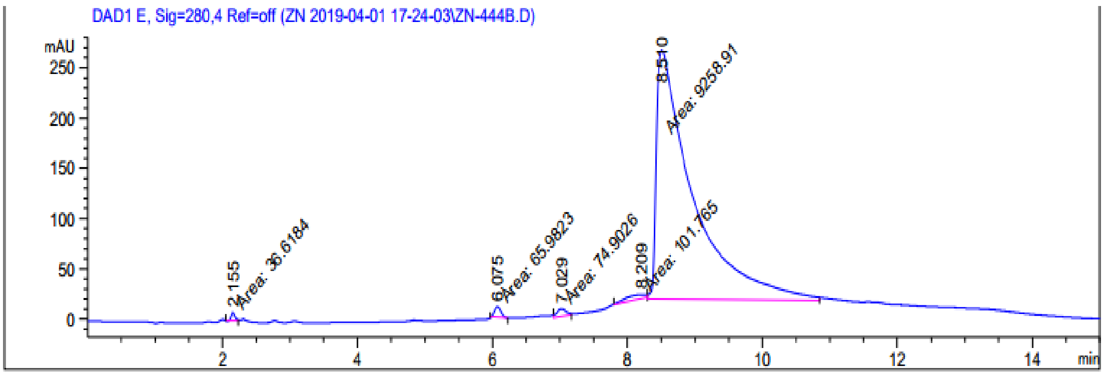

Peak #	RetTime [min]	Type	Width [min]	Area [mAU*s]	Height [mAU]	Area %
1	2.155	MM	0.0723	36.61839	8.44448	0.3839
2	6.075	MM	0.1088	65.98235	10.10817	0.6918
3	7.029	MM	0.1636	74.90263	7.62938	0.7853
4	8.209	MM	0.3125	101.76469	3.98740	1.0669
5	8.510	MM	0.6232	9258.90918	247.60678	97.0721

Fig.52. The HPLC for **14f**

Compound 14g

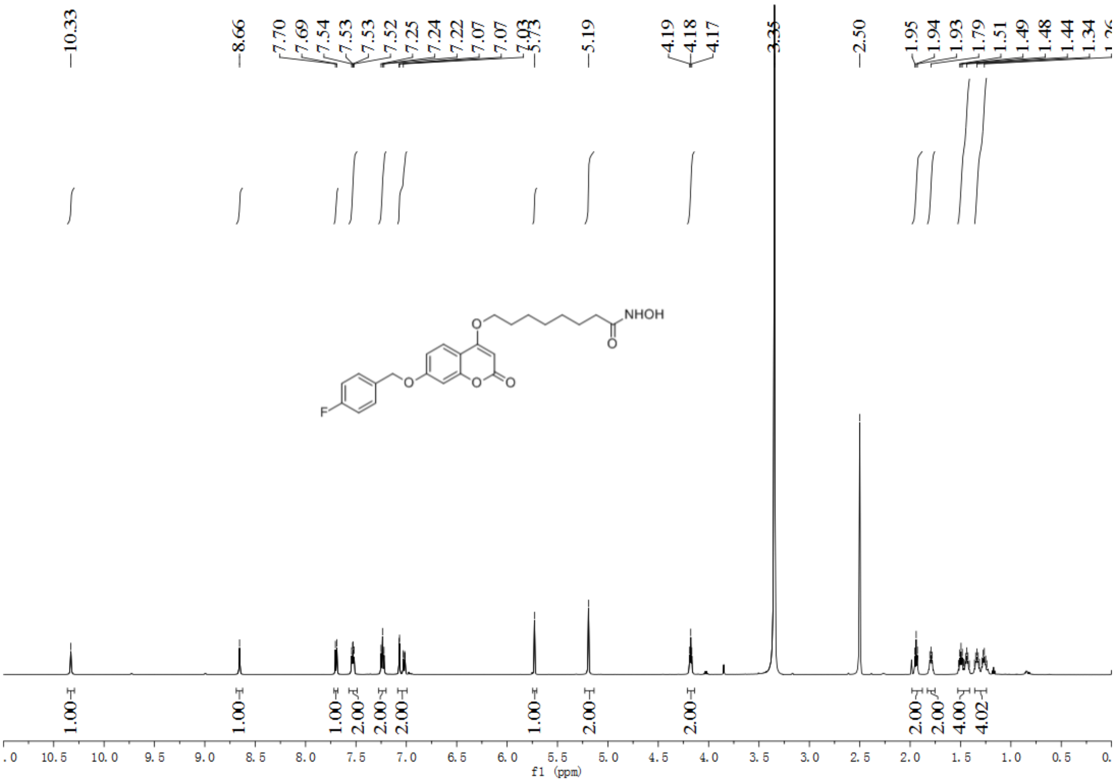

Fig.53. The ^{1}H NMR spectrum for **14g**

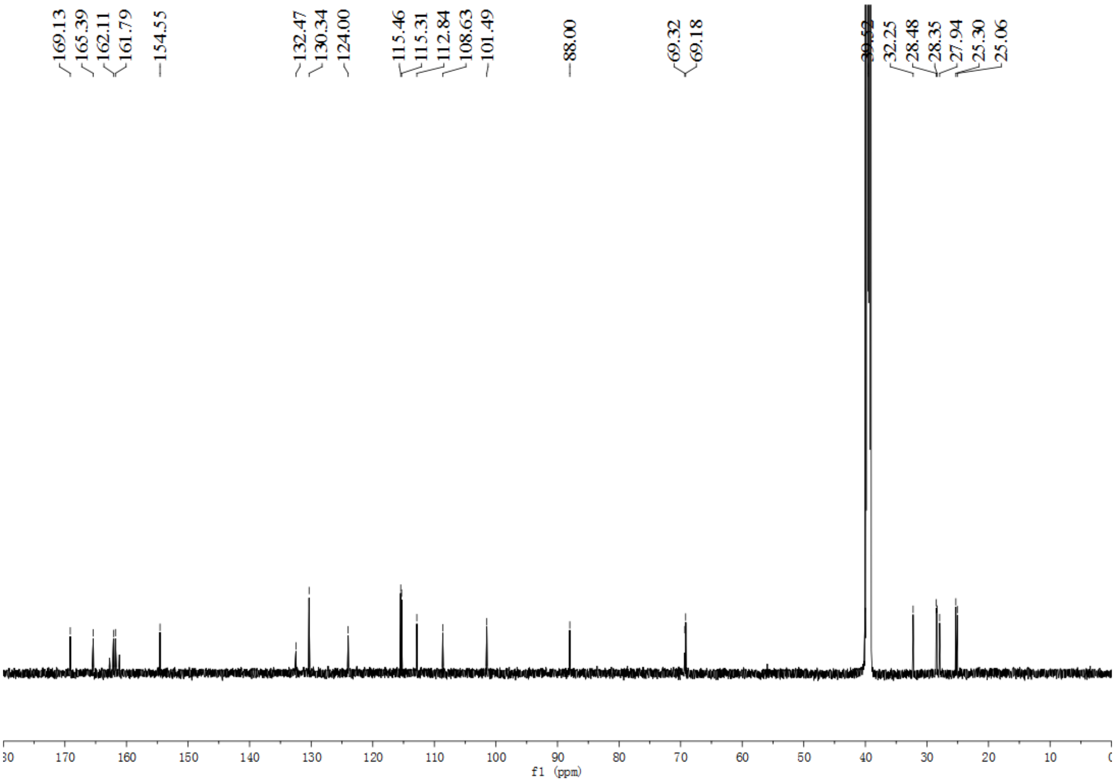

Fig.54. The ^{13}C NMR spectrum for **14g**

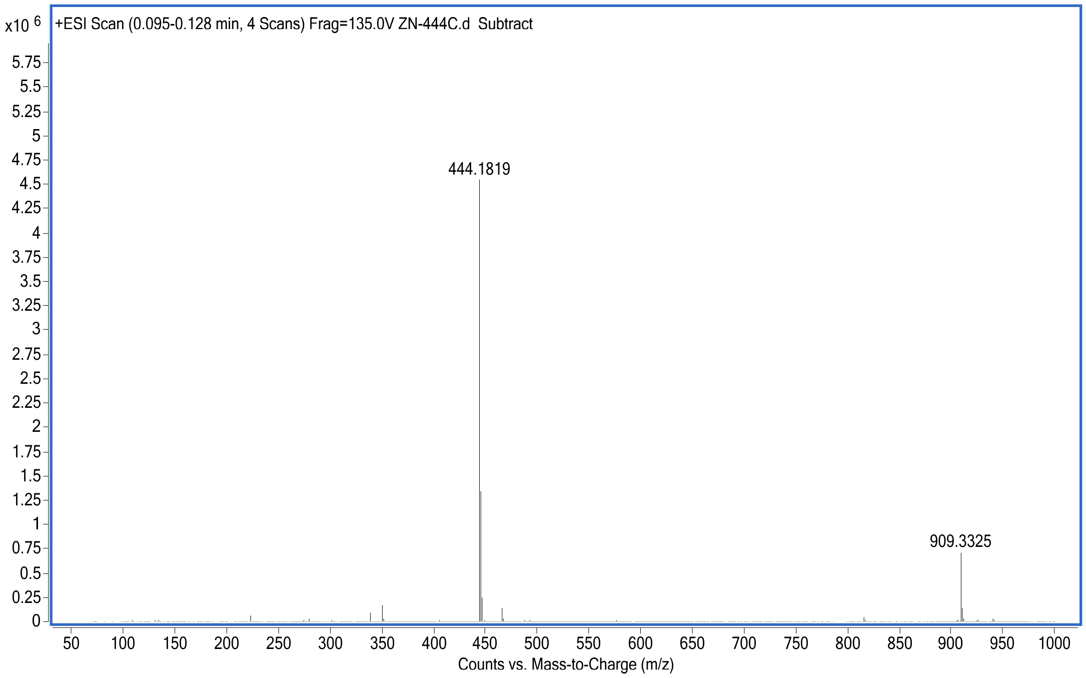

Fig.55. The HR MS spectrum for **14g**

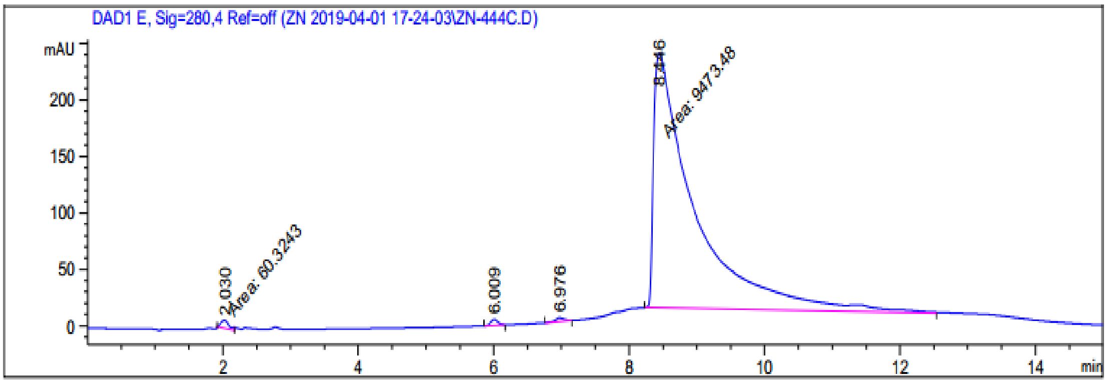

Peak #	RetTime [min]	Type	Width [min]	Area [mAU*s]	Height [mAU]	Area %
1	2.030	MM	0.1405	60.32434	7.15807	0.6283
2	6.009	BB	0.1112	37.94304	5.31988	0.3952
3	6.976	BB	0.1332	28.83160	3.19867	0.3003
4	8.446	MM	0.7006	9473.48340	225.37888	98.6761

Fig.56. The HPLC for **14g**

Compound 14h

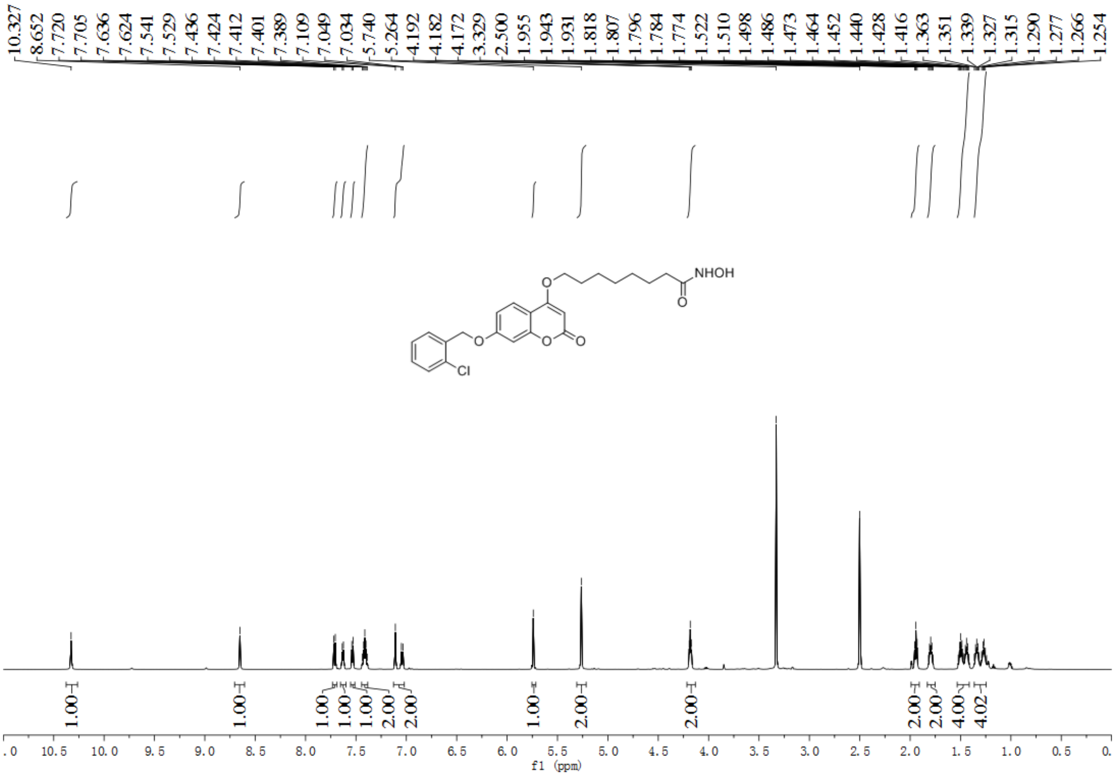

Fig.57. The ^{1}H NMR spectrum for **14h**

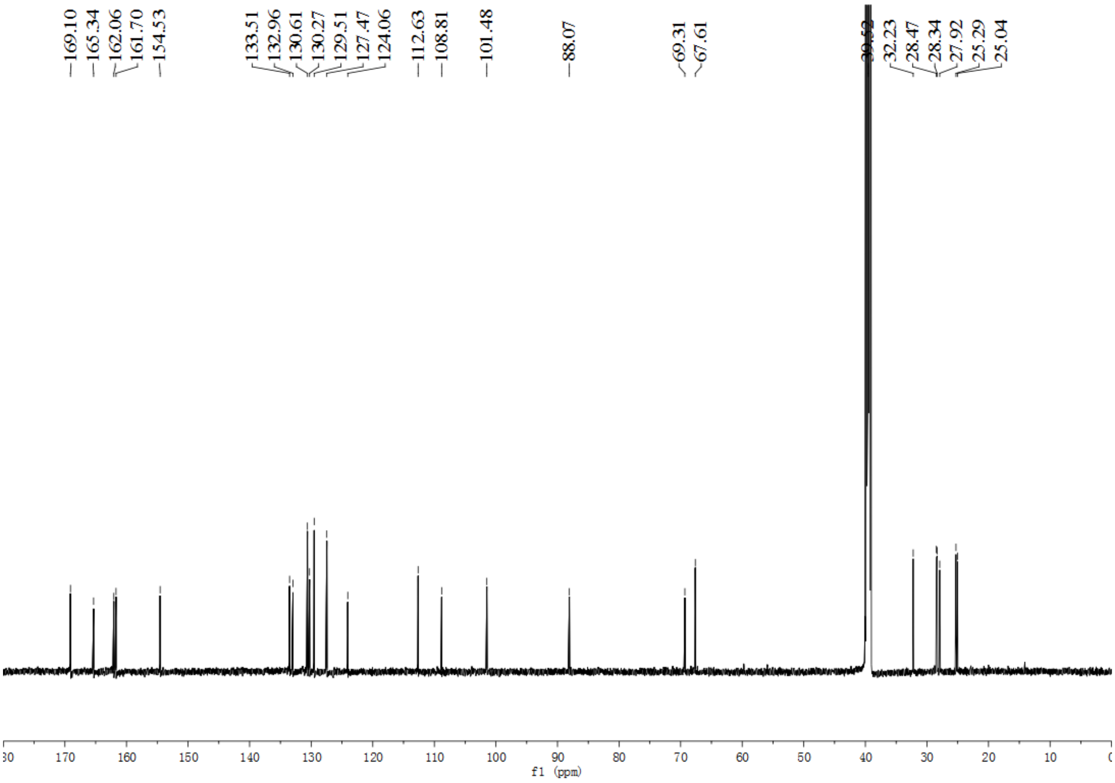

Fig.58. The ^{13}C NMR spectrum for **14h**

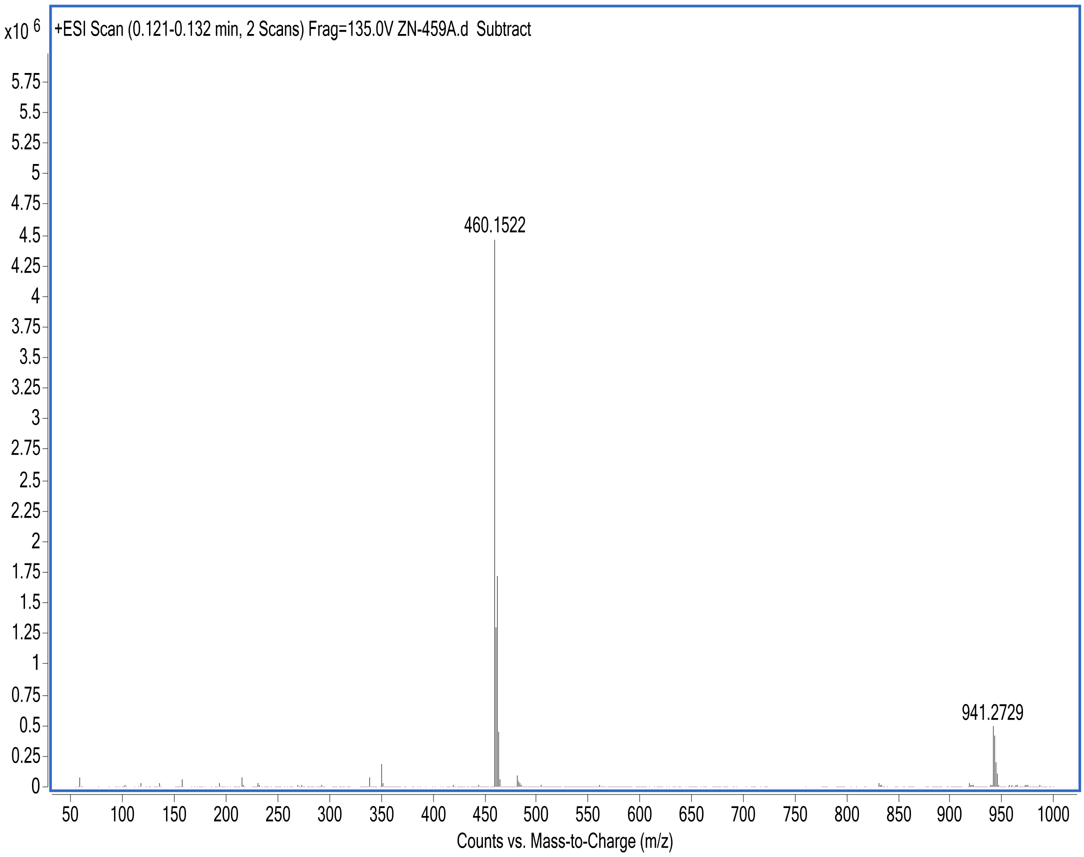

Fig.59. The HR MS spectrum for **14h**

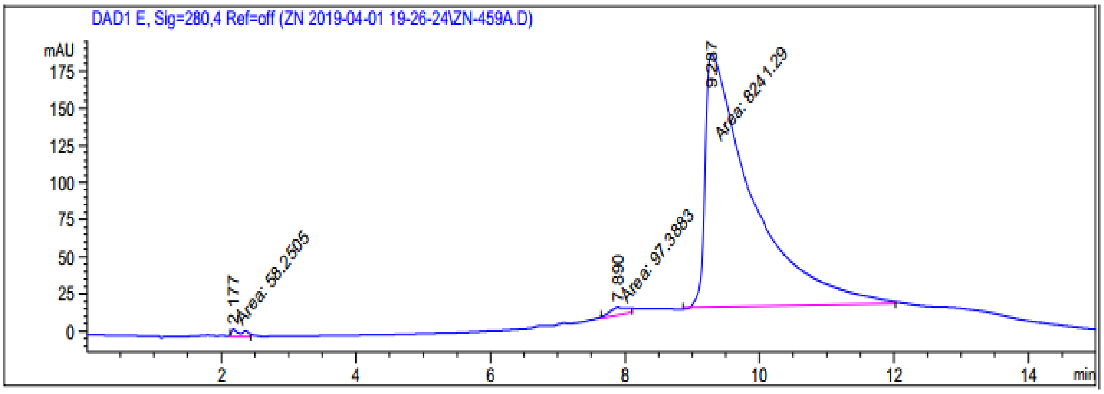

Peak #	RetTime [min]	Type	Width [min]	Area [mAU*s]	Height [mAU]	Area %
1	2.177	MM	0.1941	58.25046	5.00076	0.6937
2	7.890	MM	0.2956	97.38831	5.49125	1.1598
3	9.287	MM	0.8066	8241.28516	170.29294	98.1465

Fig.60. The HPLC for **14h**

Compound 14i

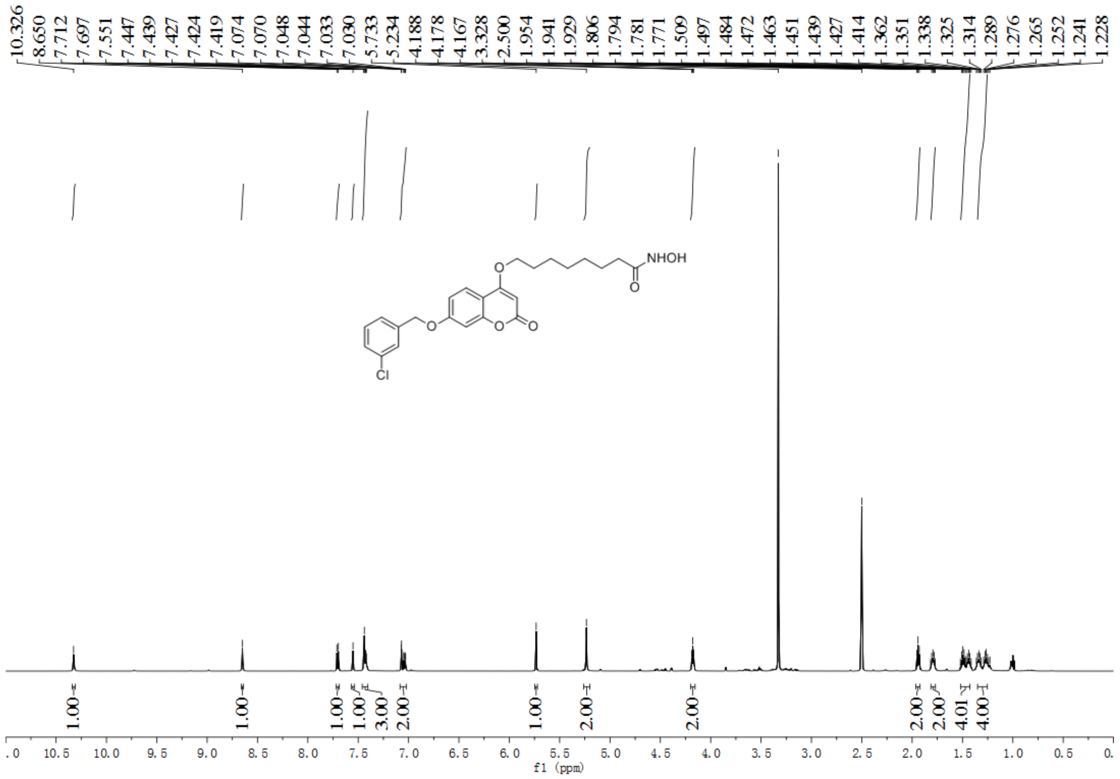

Fig.61. The ^{1}H NMR spectrum for **14i**

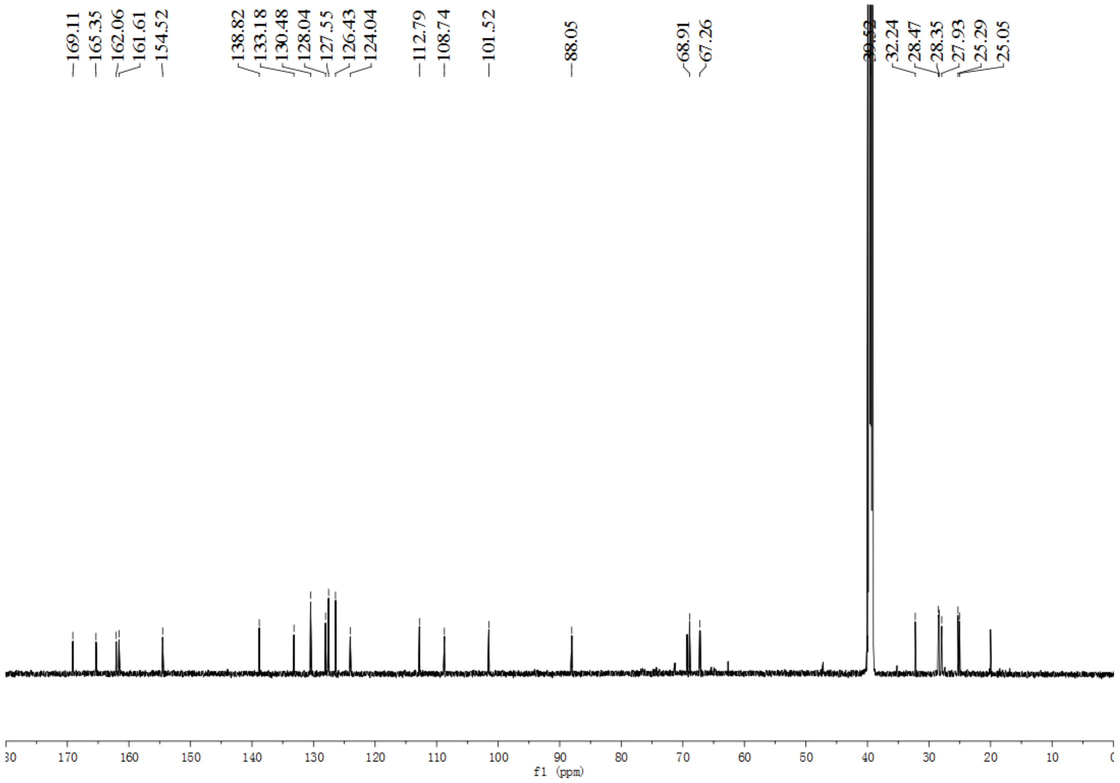

Fig.62. The ^{13}C NMR spectrum for **14i**

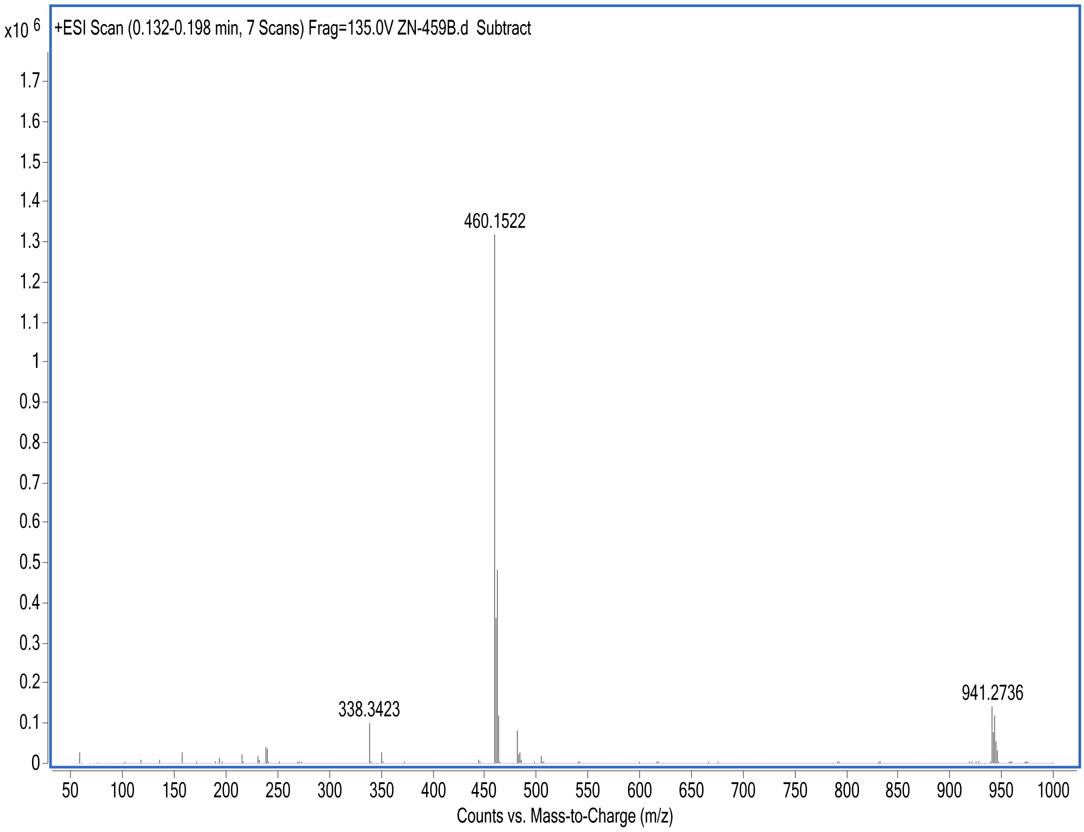

Fig.63. The HR MS spectrum for **14i**

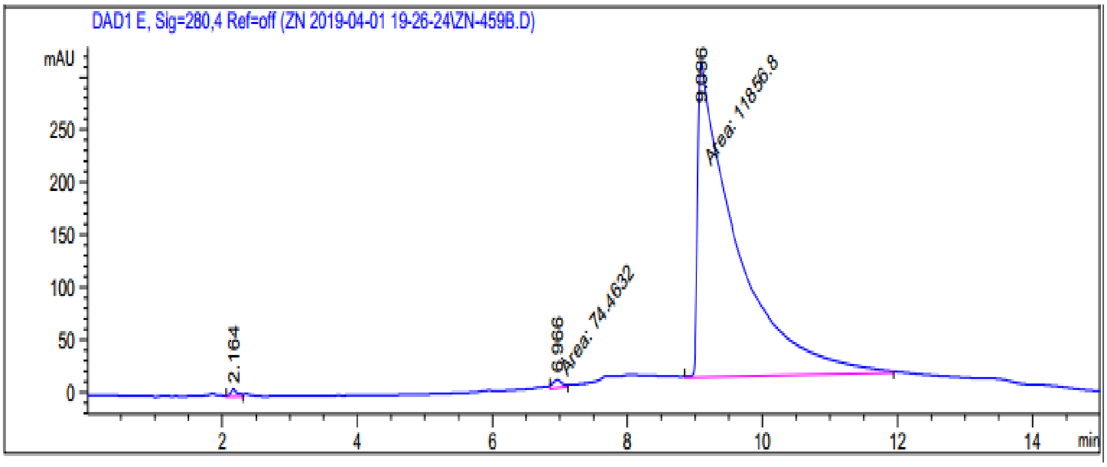

Peak #	RetTime [min]	Type	Width [min]	Area [mAU*s]	Height [mAU]	Area %
1	2.164	VV	0.0991	48.78223	6.85082	0.4072
2	6.966	MM	0.1576	74.46319	7.87577	0.6216
3	9.096	MM	0.6594	1.18568e4	299.69501	98.9712

Fig.64. The HPLC for **14i**

Compound 14j

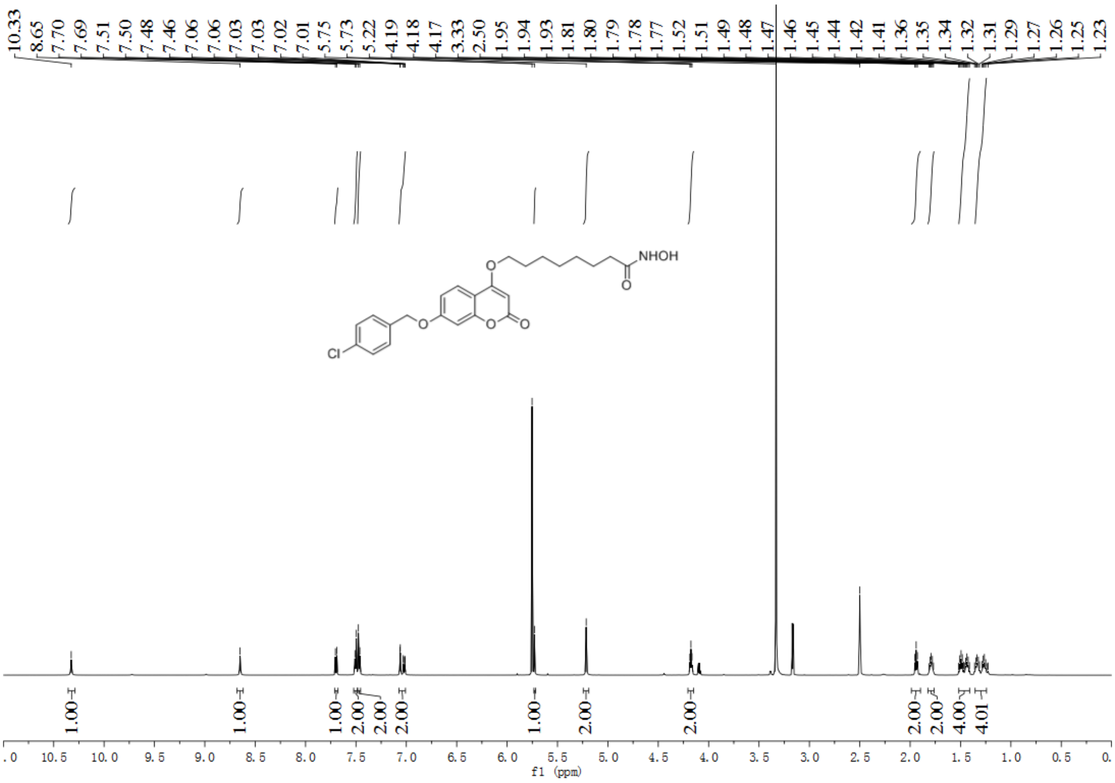

Fig.65. The ^{1}H NMR spectrum for **14j**

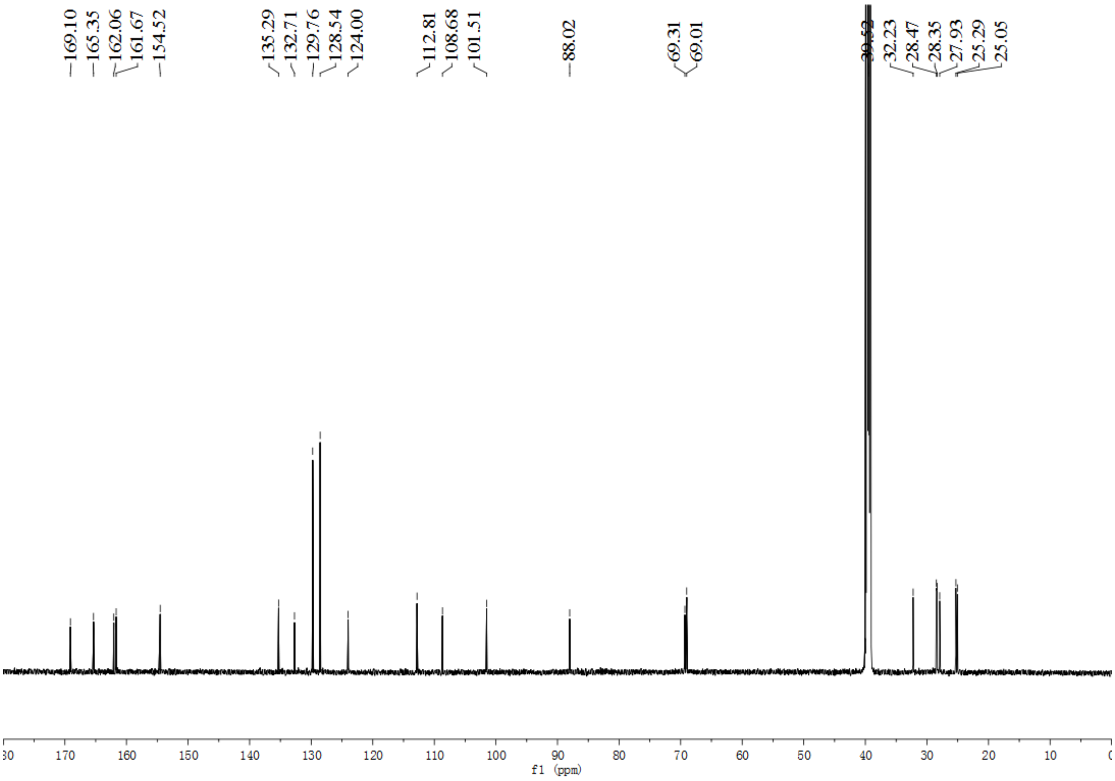

Fig.66. The ^{13}C NMR spectrum for **14j**

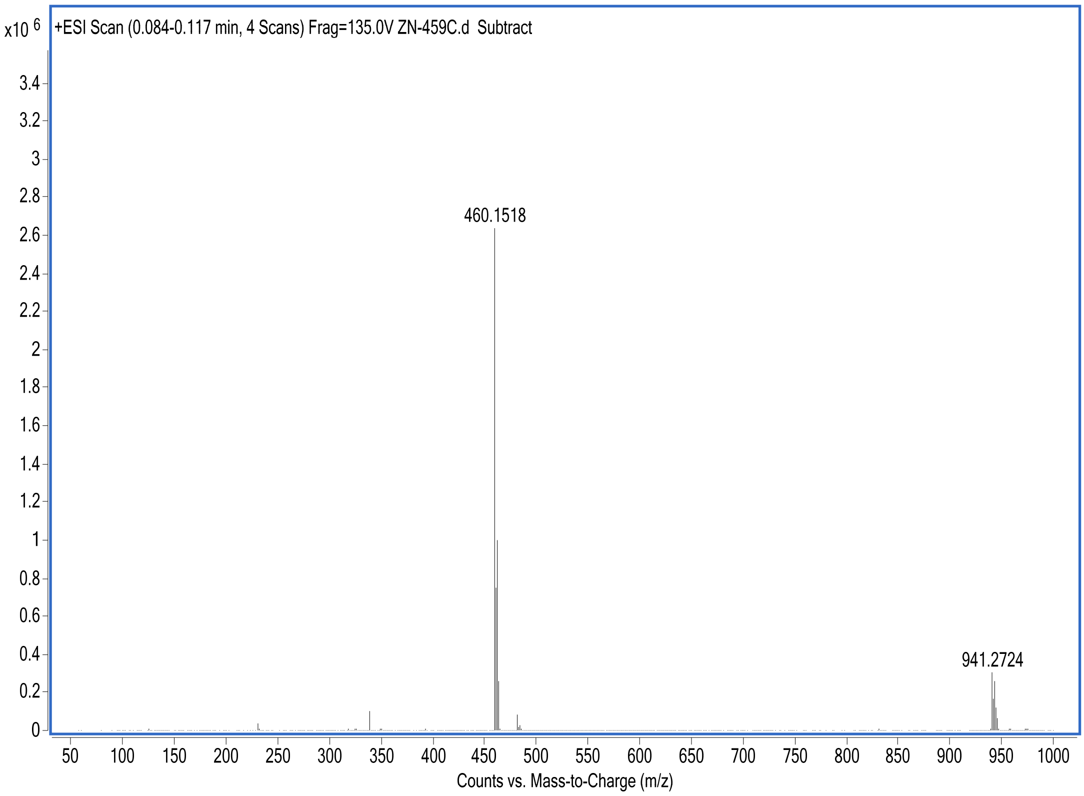

Fig.67. The HR MS spectrum for **14j**

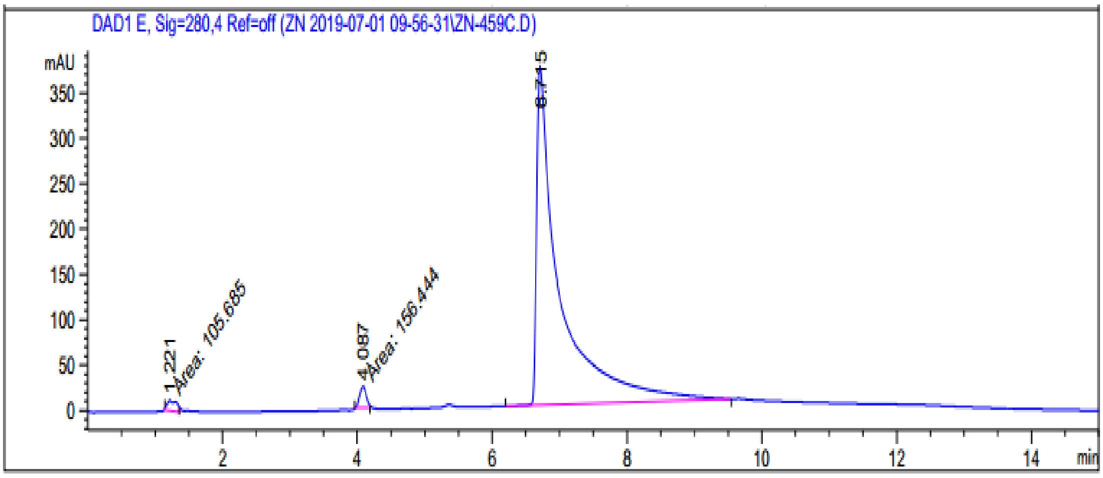

Peak #	RetTime [min]	Type	Width [min]	Area [mAU*s]	Height [mAU]	Area %
1	1.221	MM	0.1631	105.68490	10.79665	1.1535
2	4.087	MM	0.1118	156.44432	23.31689	1.7075
3	6.715	BB	0.3149	8900.29883	372.27359	97.1391

Fig.68. The HPLC for **14j**

Compound 14k

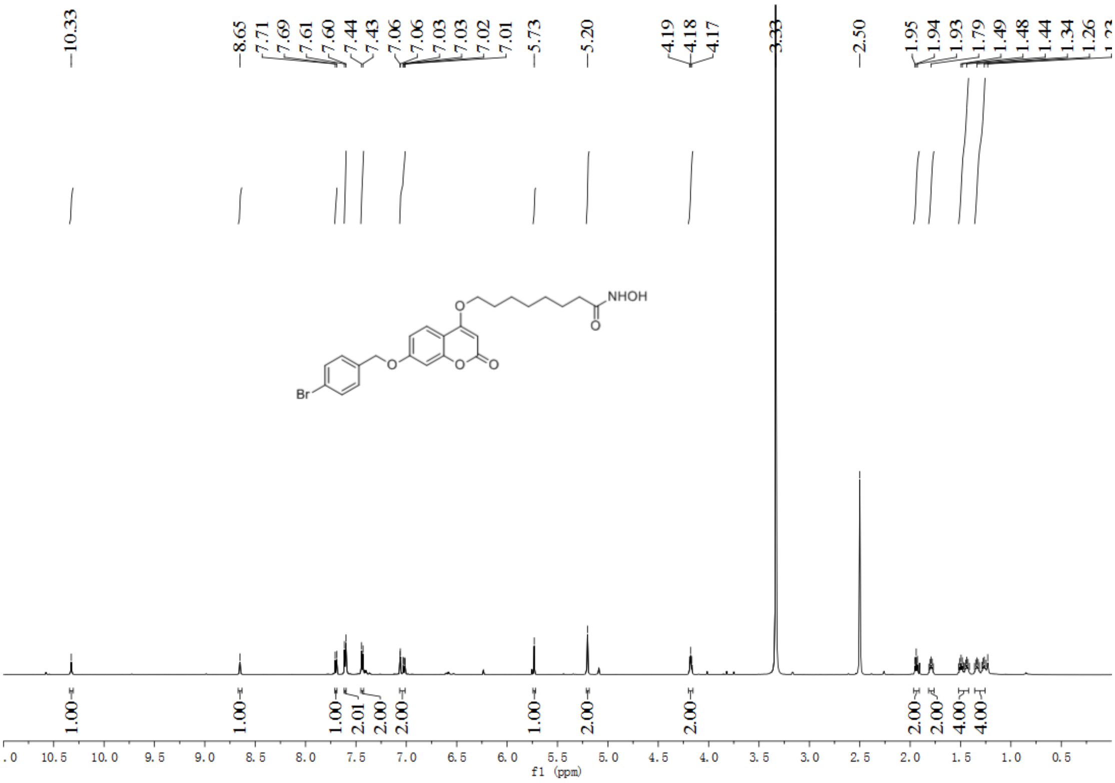

Fig.69. The ^{1}H NMR spectrum for **14k**

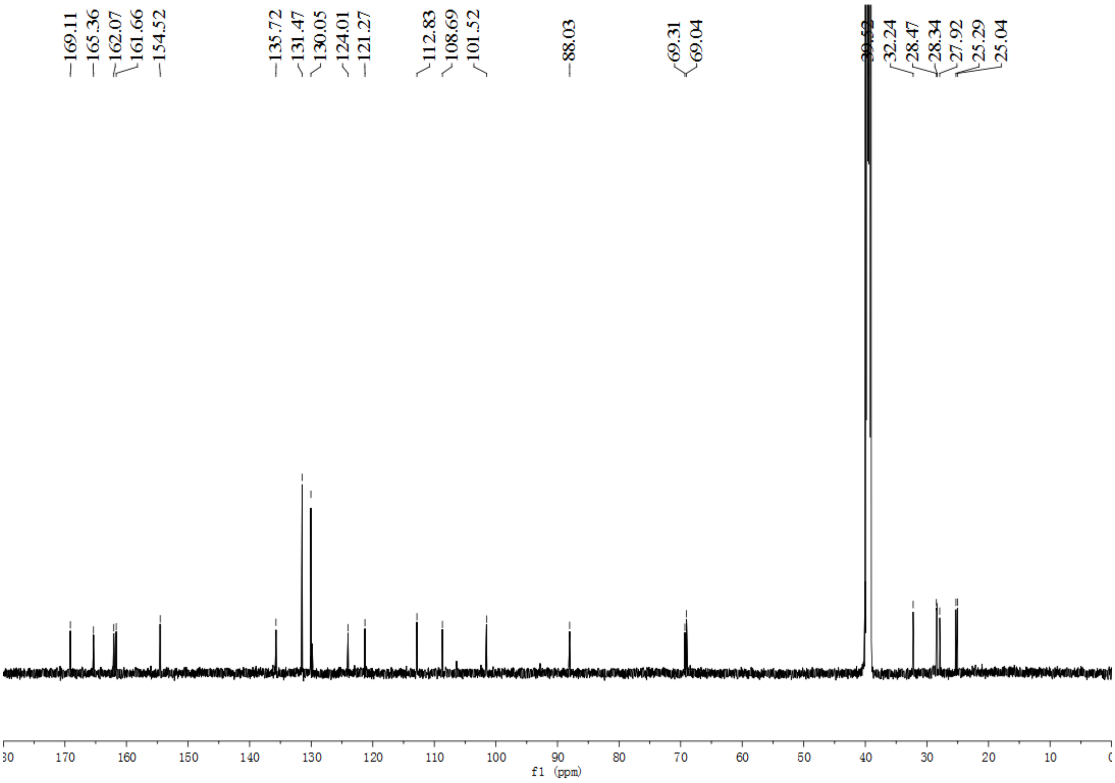

Fig.70. The ^{13}C NMR spectrum for **14k**

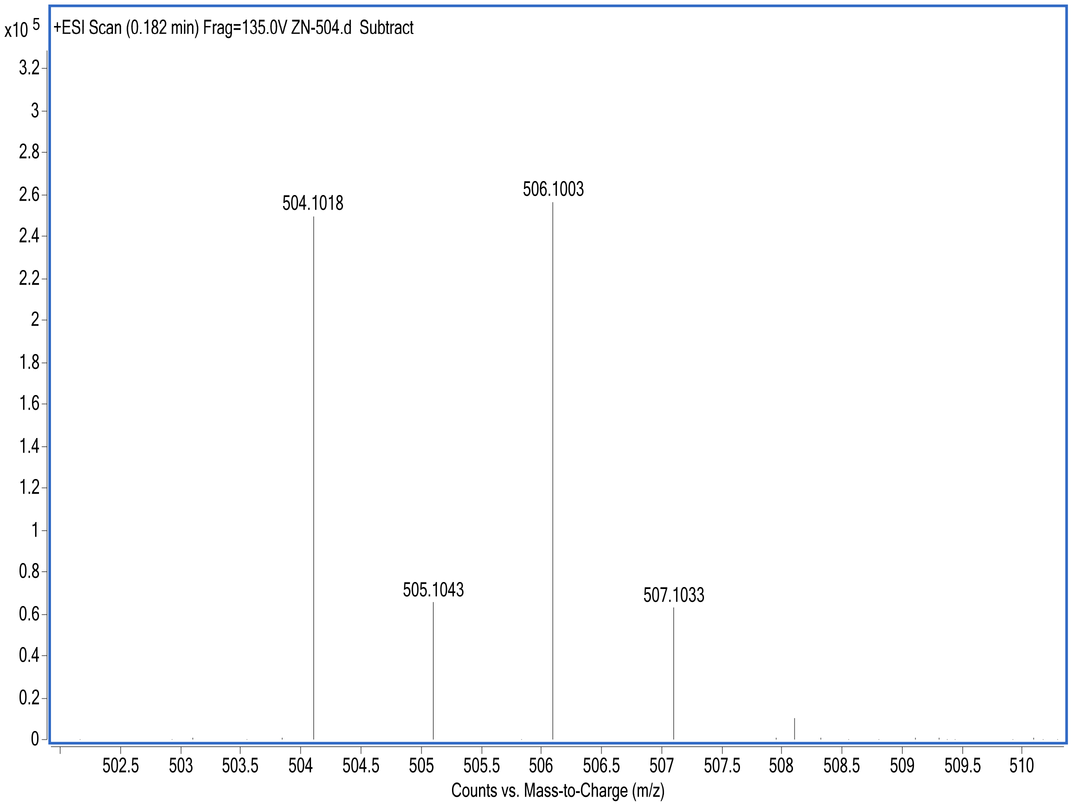

Fig.71. The HR MS spectrum for **14k**

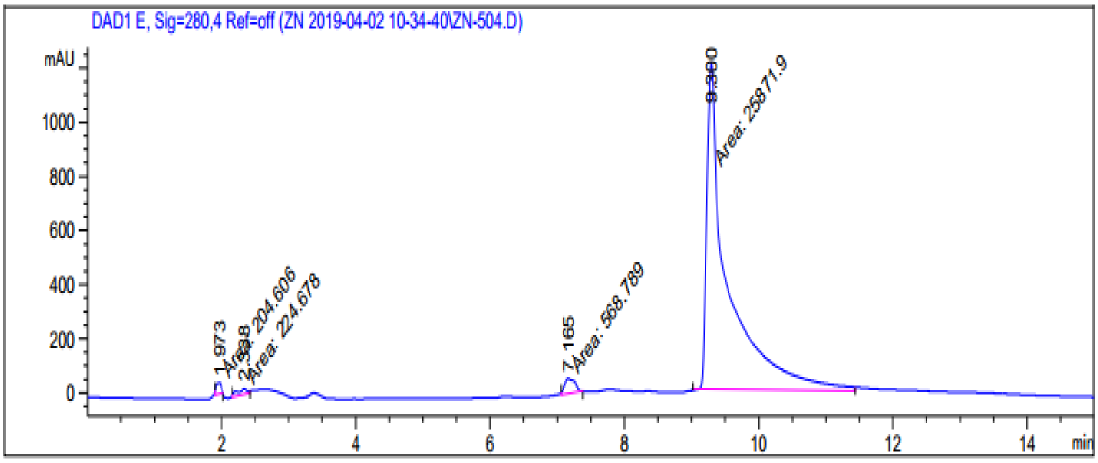

Peak #	RetTime [min]	Type	Width [min]	Area [mAU*s]	Height [mAU]	Area %
1	1.973	MM	0.0847	204.60579	40.24200	0.7615
2	2.338	MM	0.1731	224.67801	21.63332	0.8362
3	7.165	MM	0.1697	568.78894	55.85863	2.1168
4	9.300	MM	0.3583	2.58719e4	1203.58838	96.2855

Fig.72. The HPLC for **14k**

Compound 14l

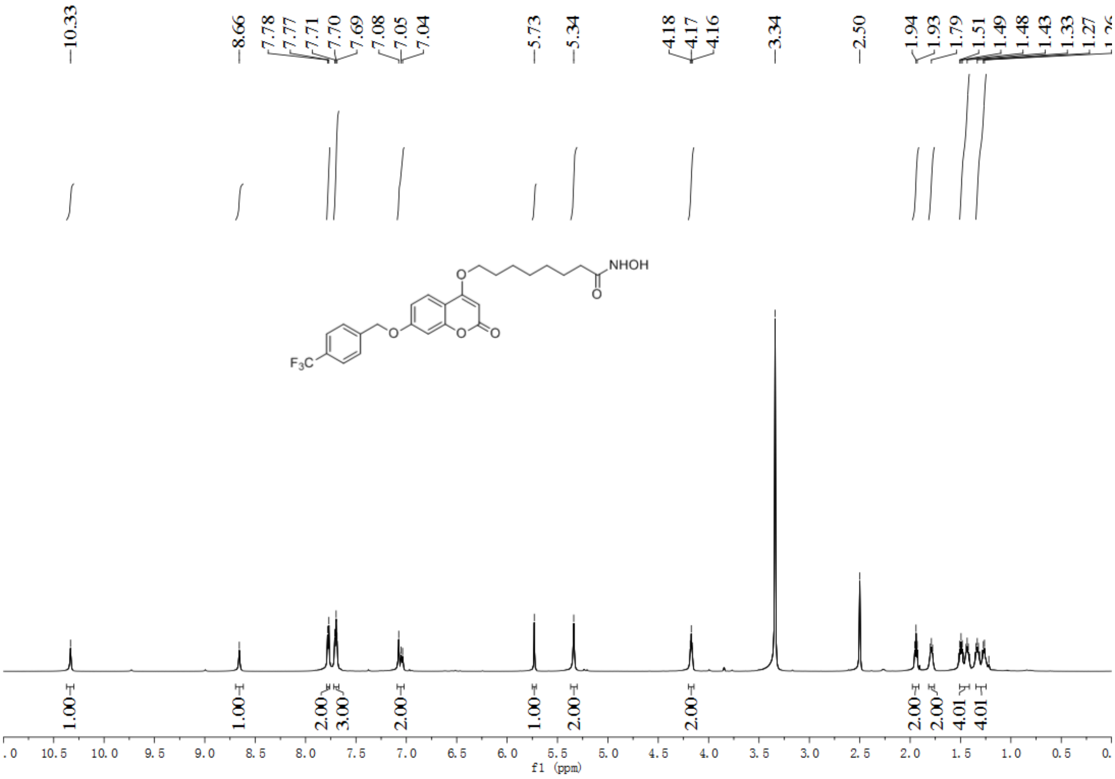

Fig.73. The ^{1}H NMR spectrum for **14l**

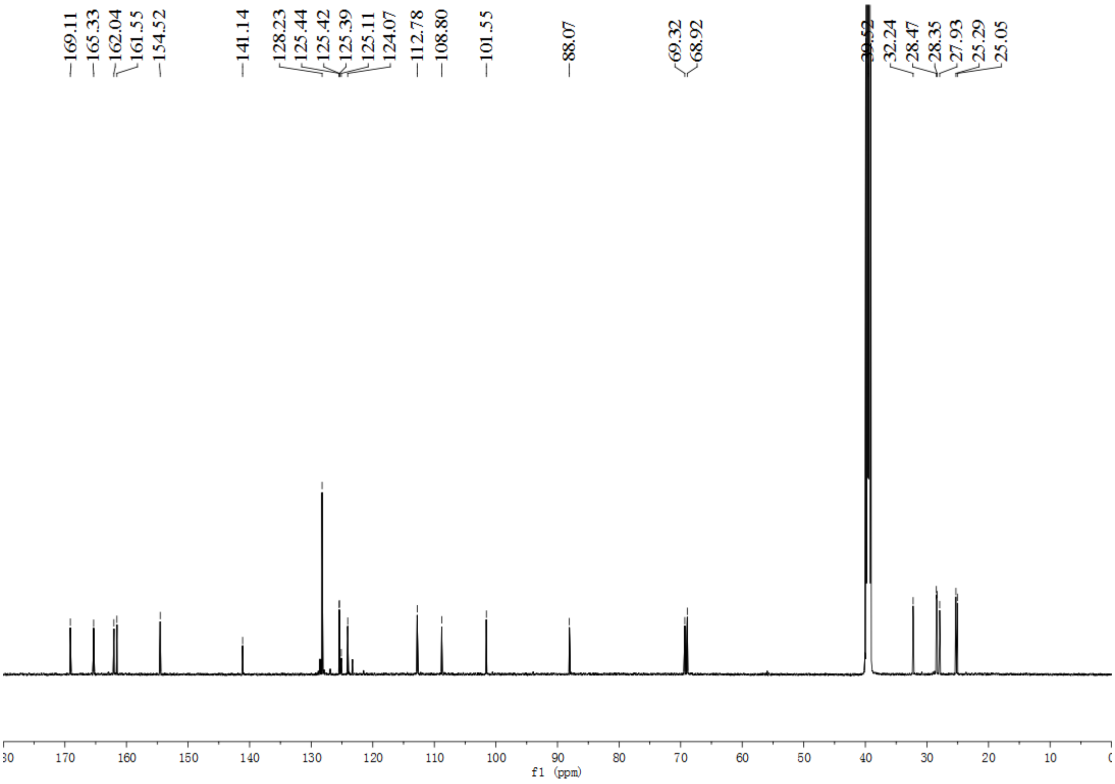

Fig.74. The ^{13}C NMR spectrum for **14l**

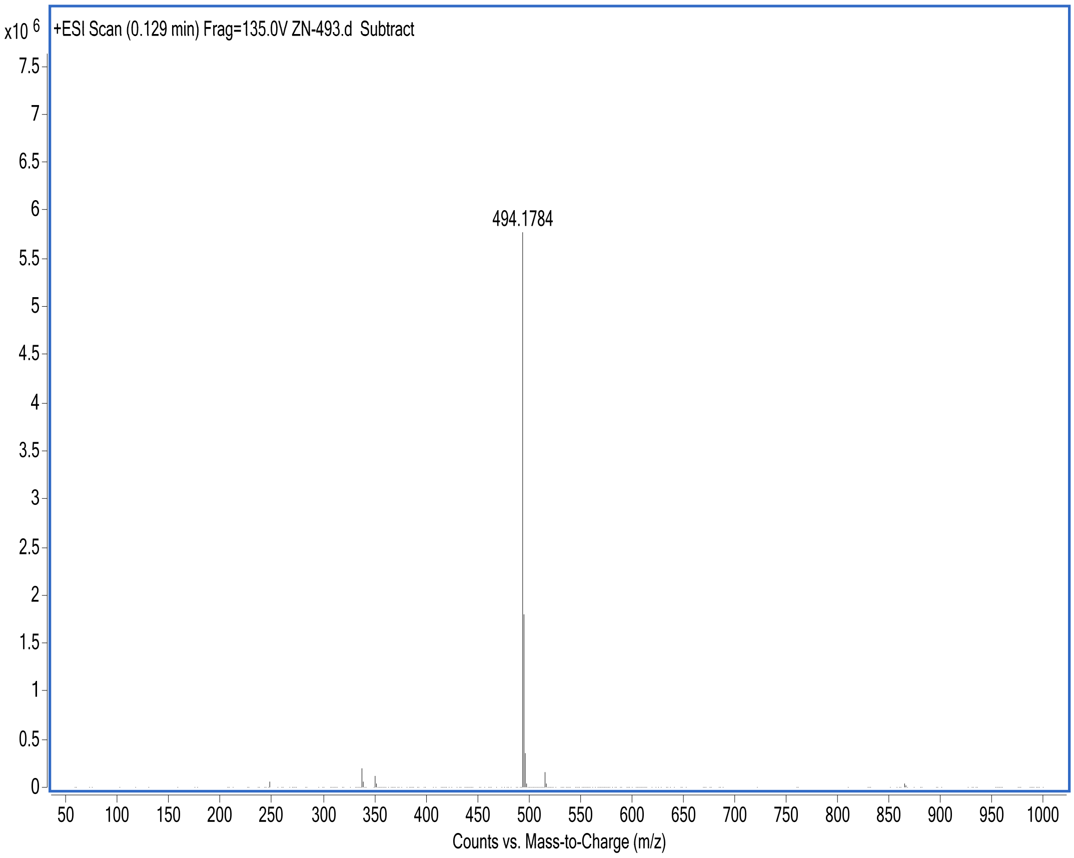

Fig.75. The HR MS spectrum for **14l**

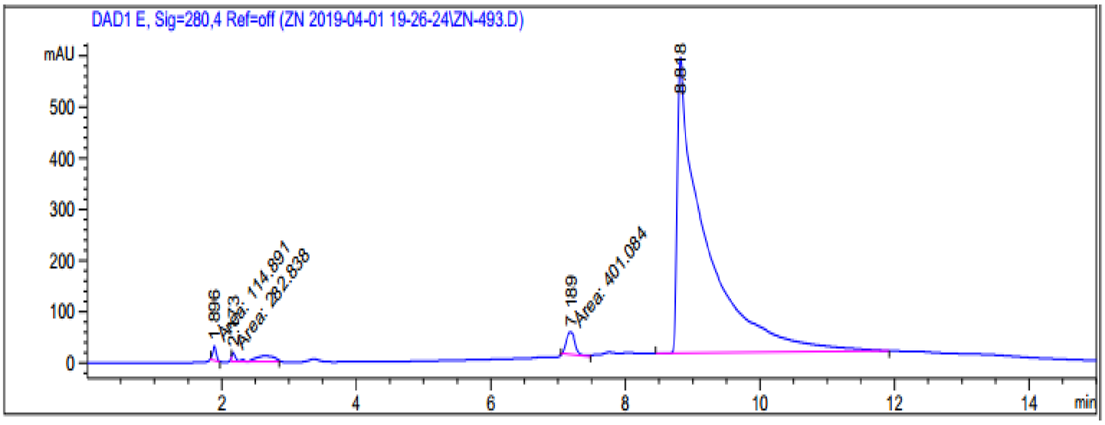

Peak #	RetTime [min]	Type	Width [min]	Area [mAU*s]	Height [mAU]	Area %
1	1.896	MM	0.0672	114.89136	28.48376	0.6577
2	2.173	MM	0.2778	282.83759	16.96699	1.6192
3	7.189	MM	0.1527	401.08353	43.78085	2.2961
4	8.818	BB	0.3603	1.66689e4	578.26117	95.4269

Fig.76. The HPLC for **14l**

Compound 14m

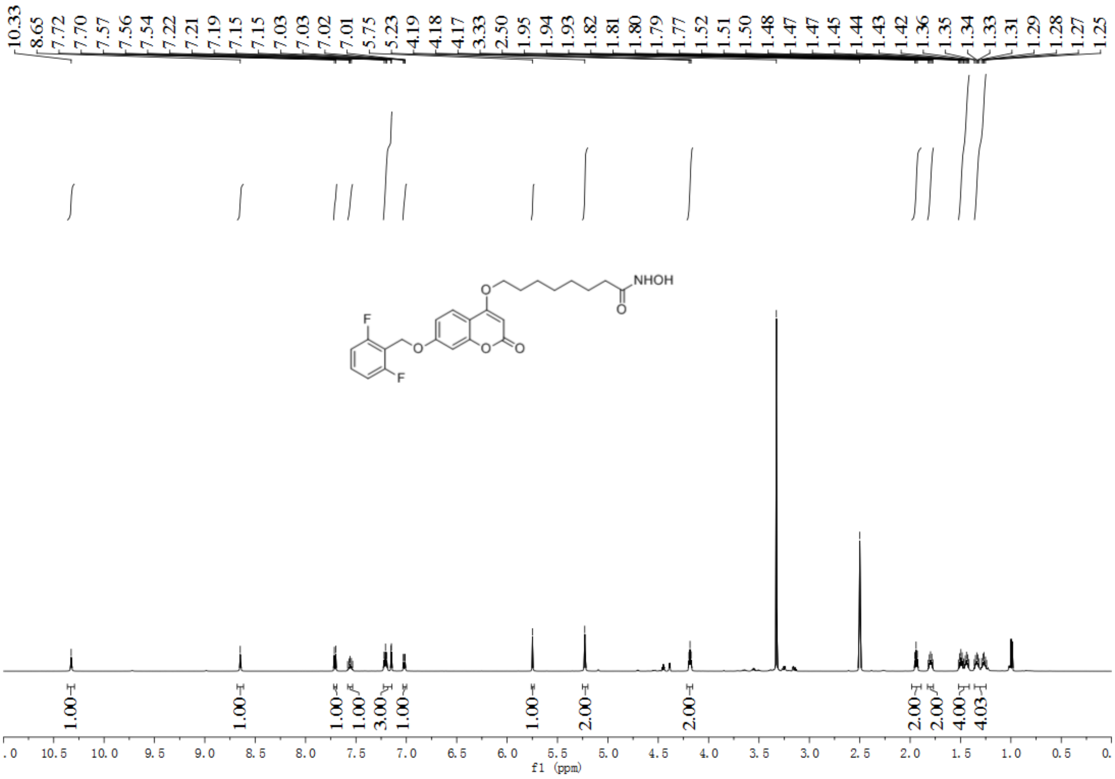

Fig.77. The ^{1}H NMR spectrum for **14m**

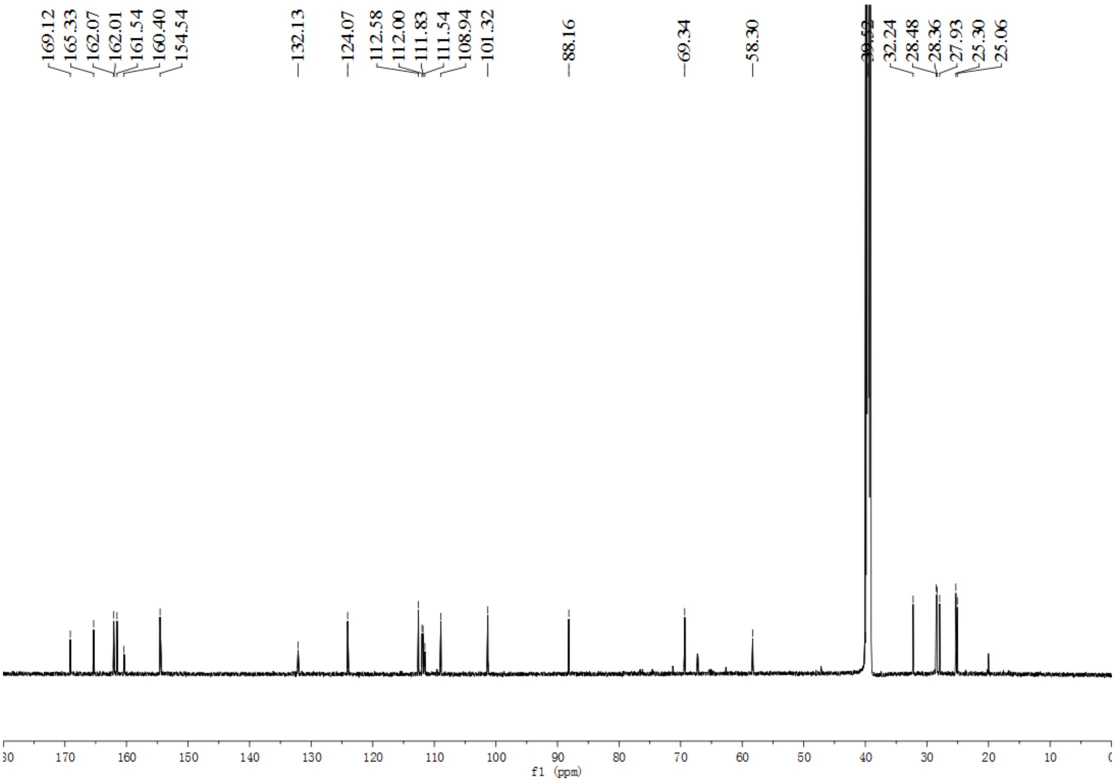

Fig.78. The ^{13}C NMR spectrum for **14m**

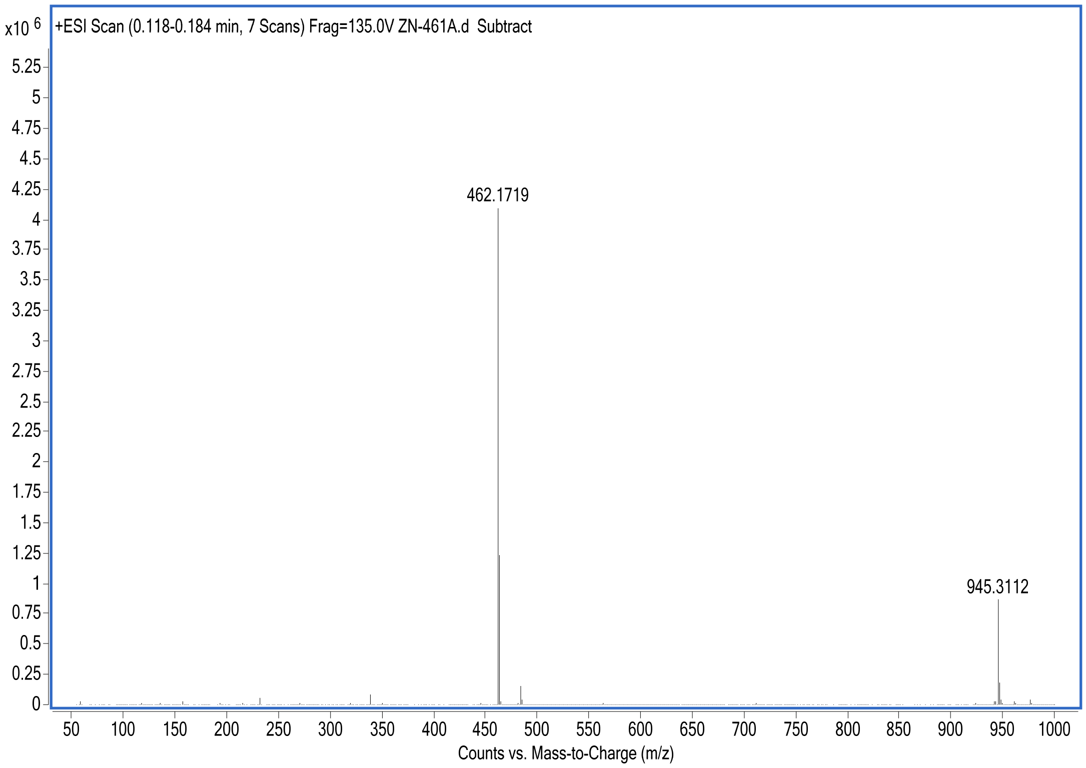

Fig.79. The HR MS spectrum for **14m**

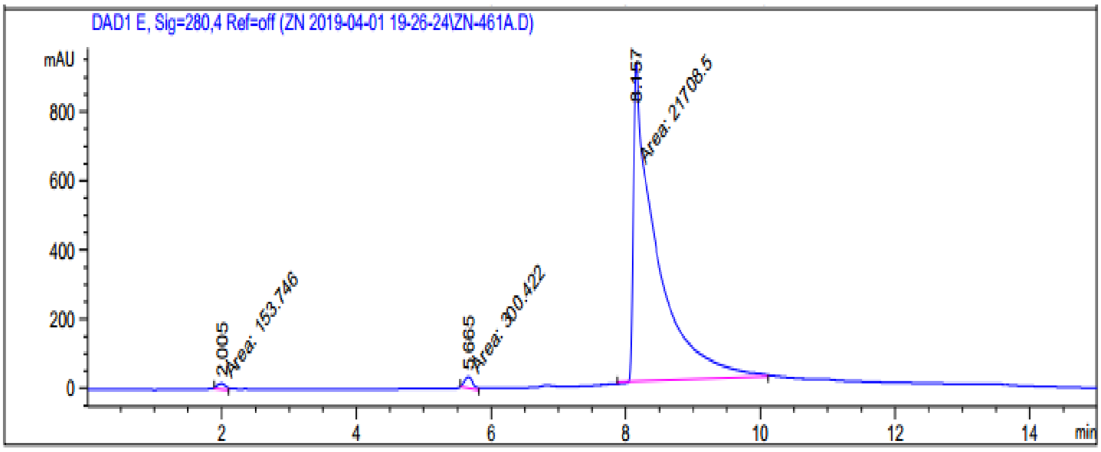

Peak #	RetTime [min]	Type	Width [min]	Area [mAU*s]	Height [mAU]	Area %
1	2.005	MM	0.1450	153.74632	17.67453	0.6937
2	5.665	MM	0.1508	300.42178	33.20177	1.3555
3	8.157	MM	0.3941	2.17085e4	918.03033	97.9508

Fig.80. The HPLC for **14m**

Compound 14n

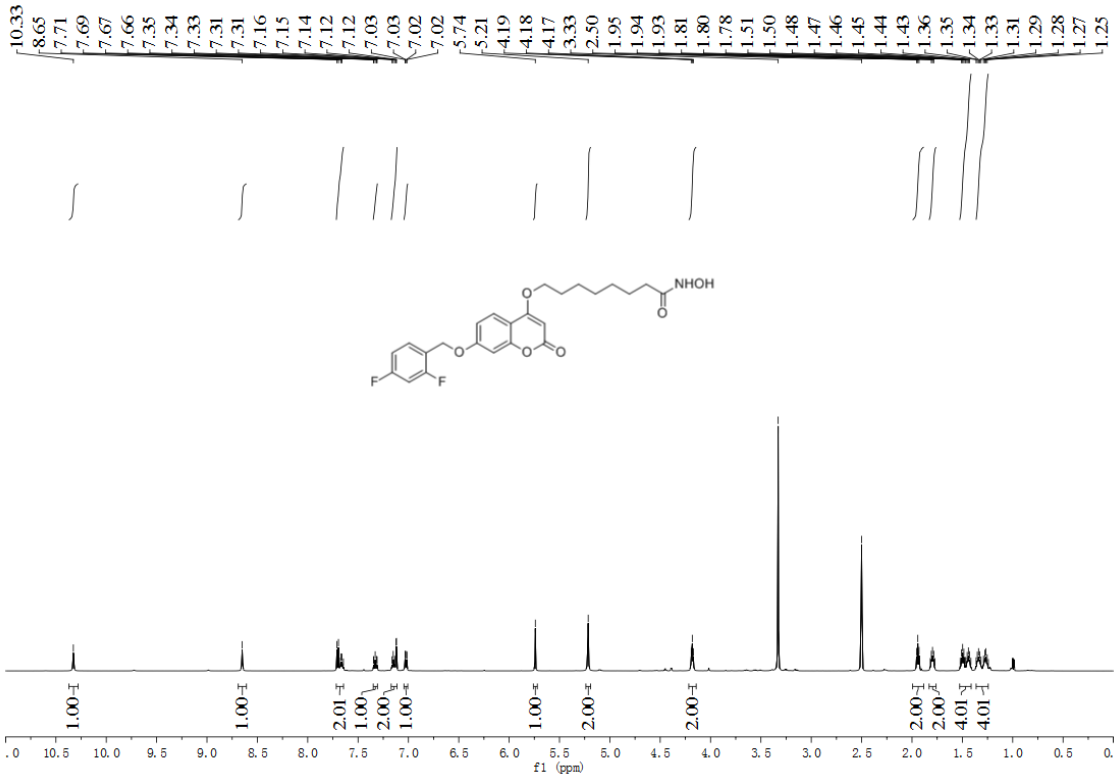

Fig.81. The ^{1}H NMR spectrum for **14n**

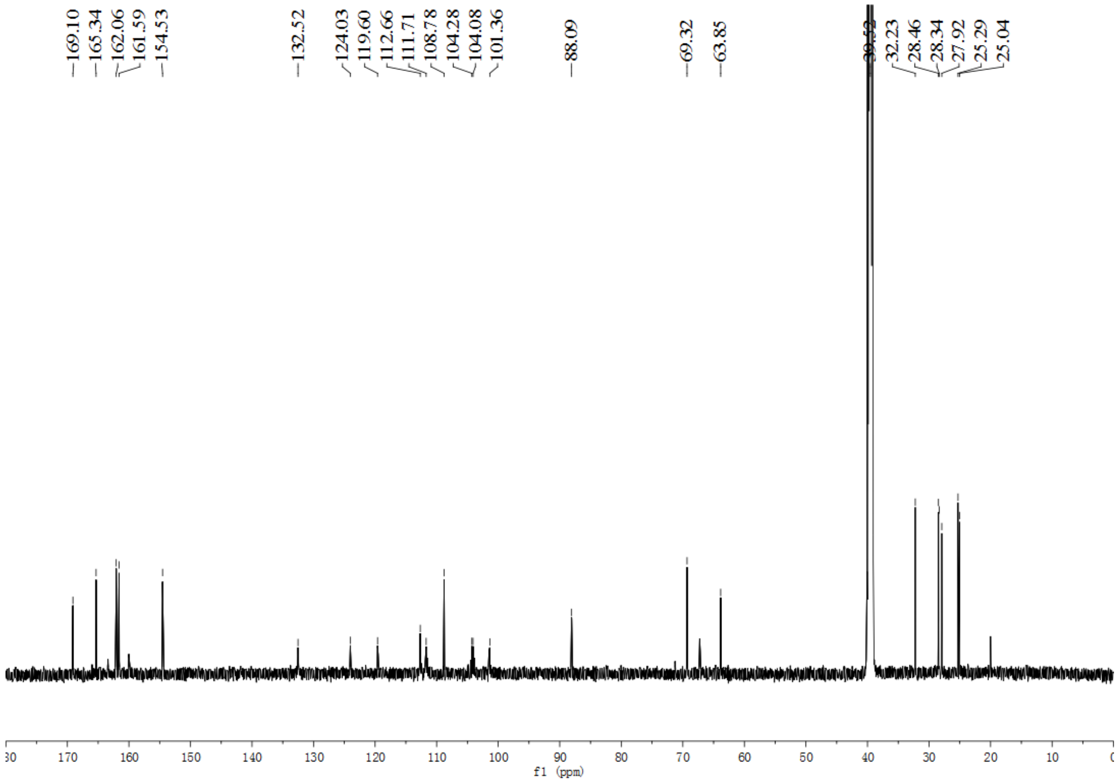

Fig.82. The ^{13}C NMR spectrum for **14n**

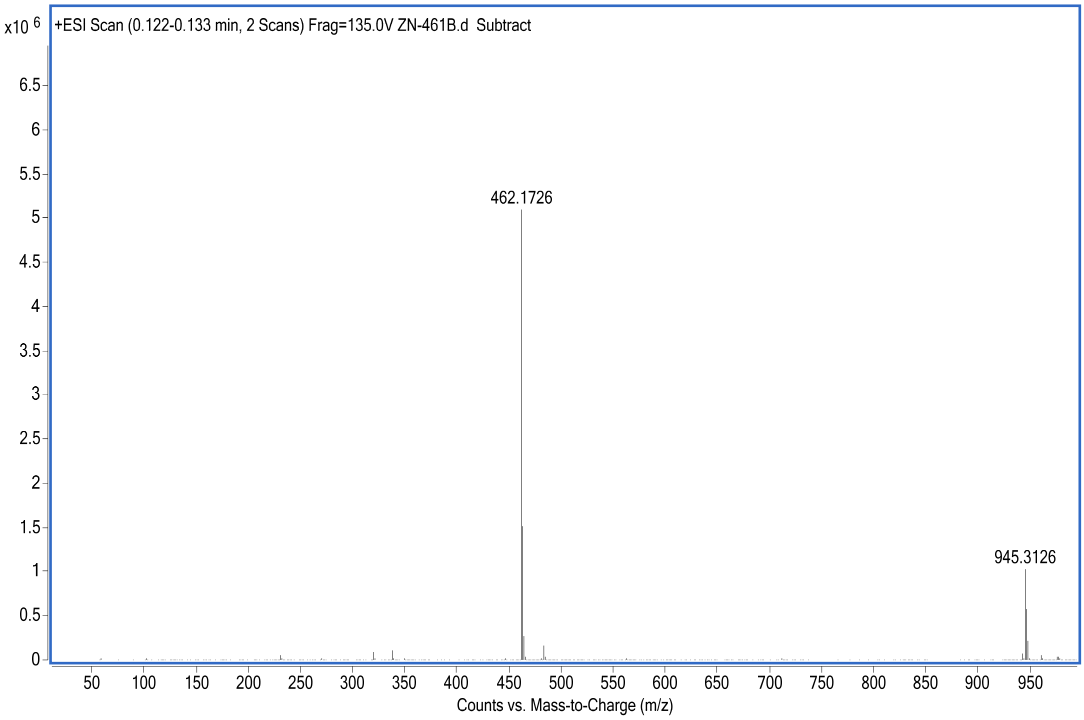

Fig.83. The HR MS spectrum for **14n**

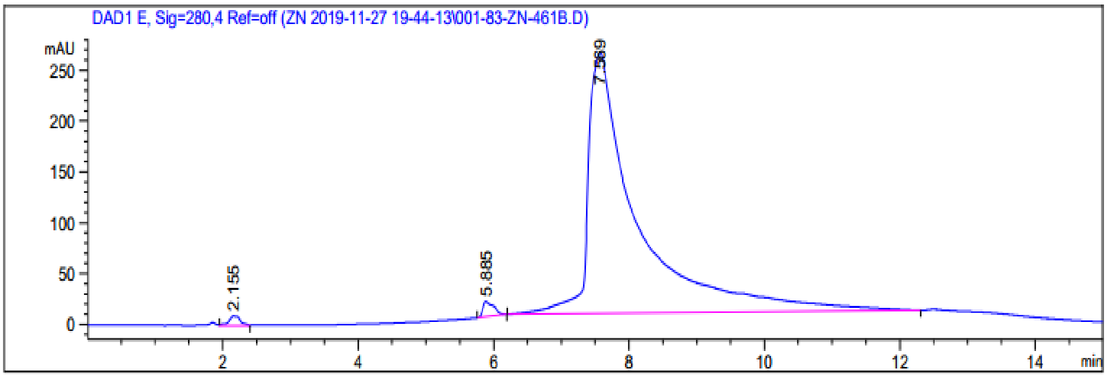

Peak #	RetTime [min]	Type	Width [min]	Area [mAU*s]	Height [mAU]	Area %
1	2.155	VB R	0.1577	108.43851	9.32556	0.8112
2	5.885	BB	0.1502	166.50893	15.15794	1.2457
3	7.569	BB	0.6883	1.30919e4	256.98825	97.9431

Fig.84. The HPLC for **14n**

Compound 14o

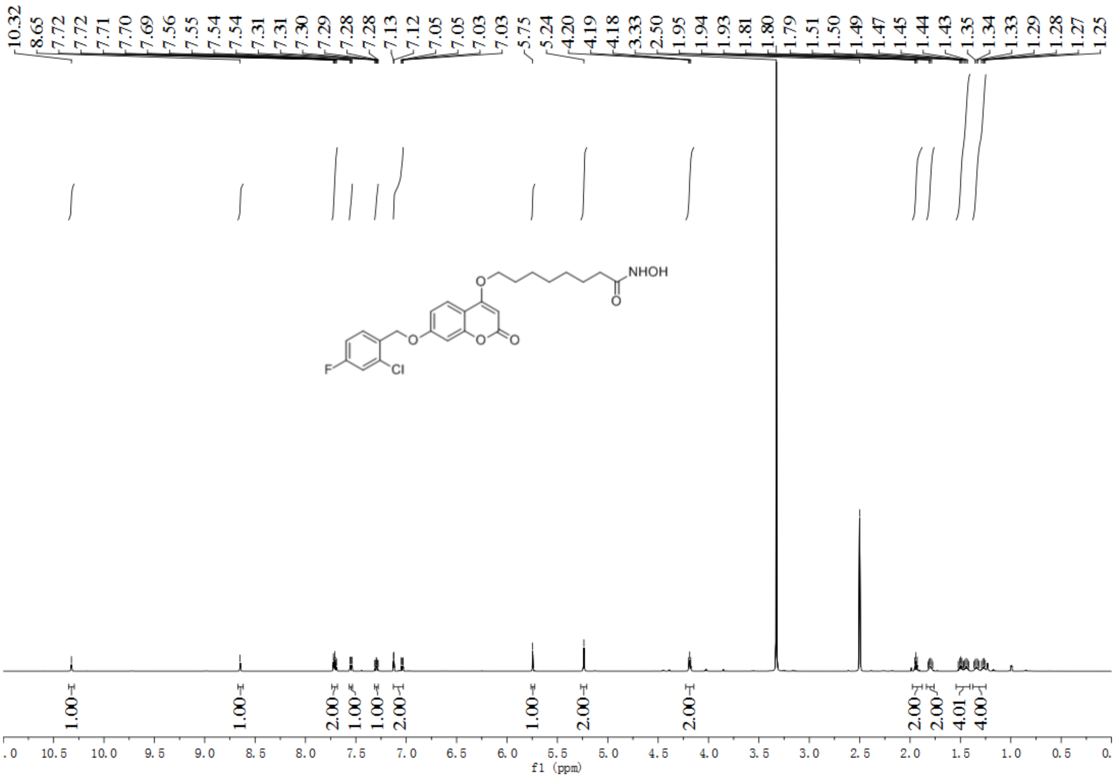

Fig.85. The ^{1}H NMR spectrum for **14o**

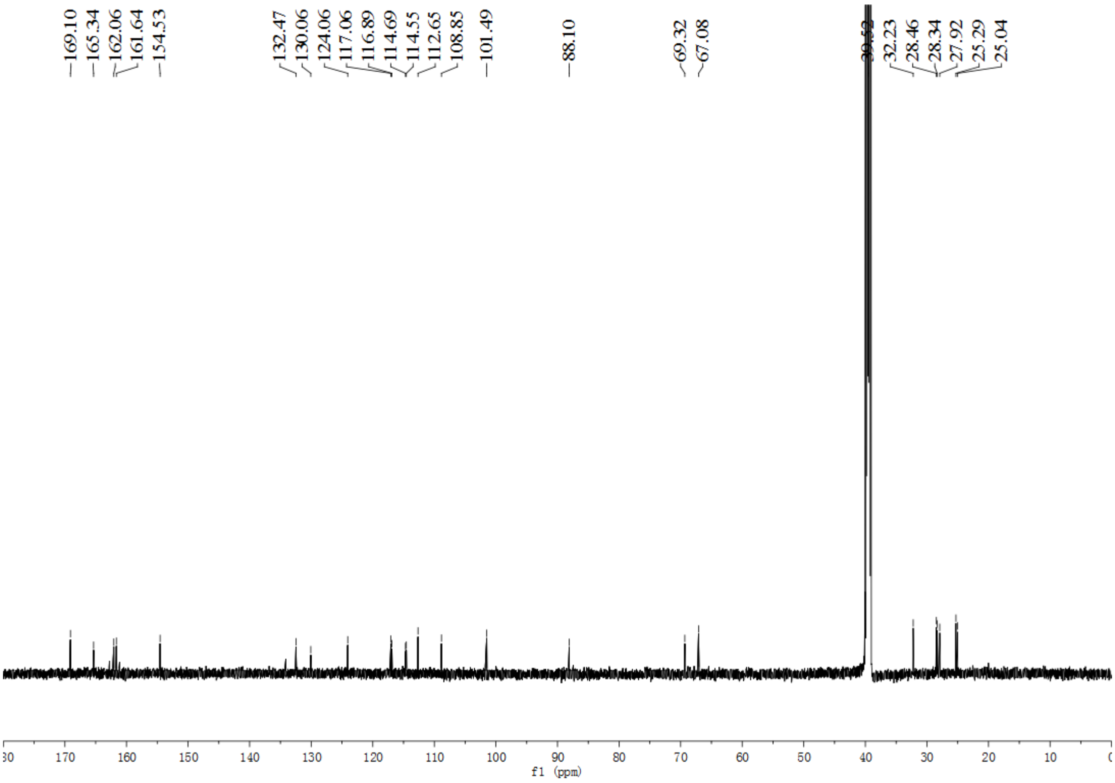

Fig.86. The ^{13}C NMR spectrum for **14o**

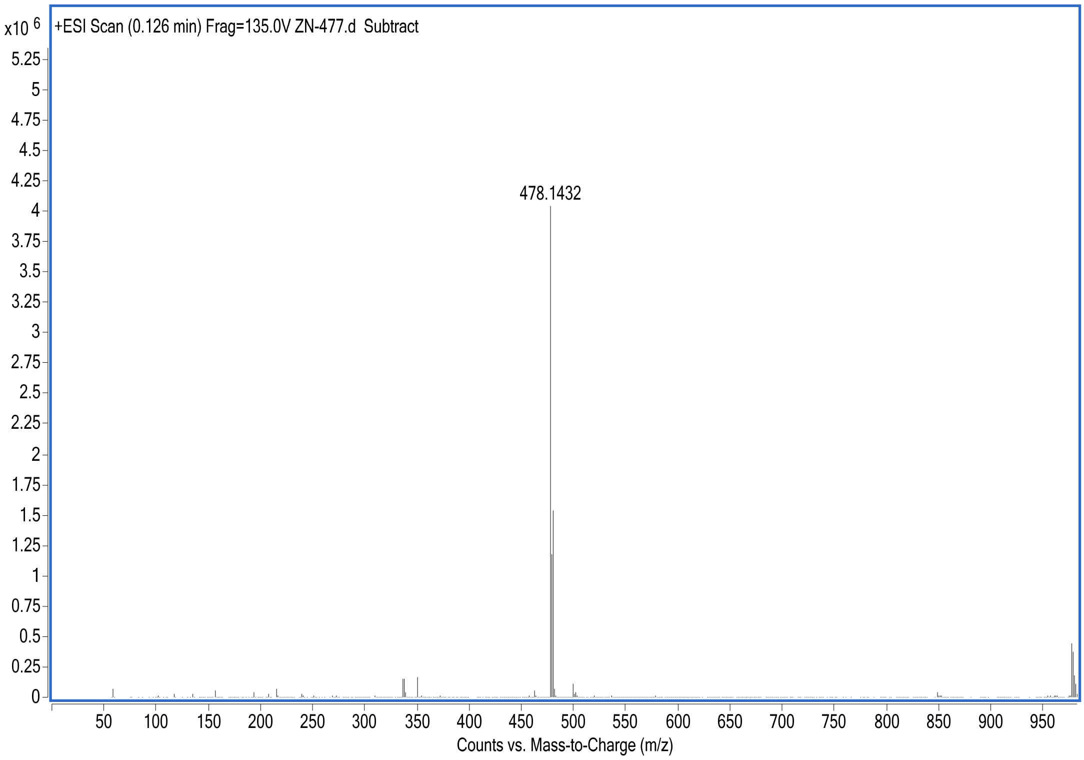

Fig.87. The HR MS spectrum for **14o**

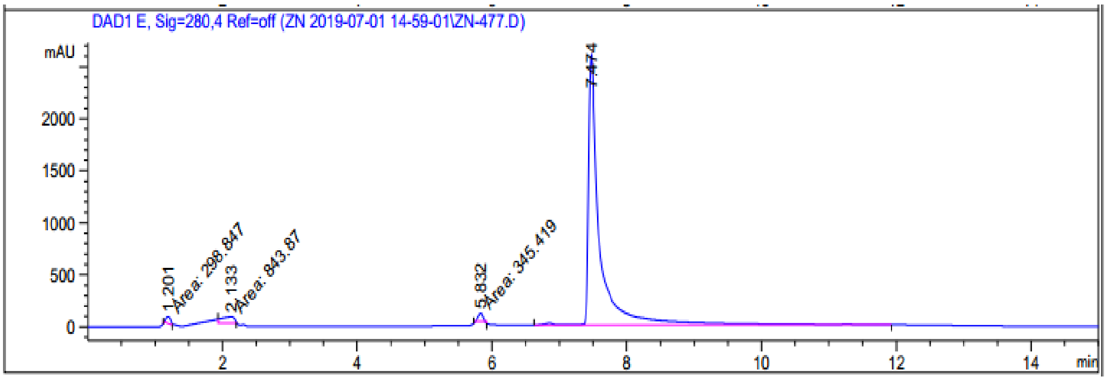

Peak #	RetTime [min]	Type	Width [min]	Area [mAU*s]	Height [mAU]	Area %
1	1.201	MM	0.0769	298.84714	64.76675	0.9287
2	2.133	MM	0.2265	843.87006	62.10731	2.6223
3	5.832	MM	0.0760	345.41922	75.70790	1.0734
4	7.474	VV R	0.1611	3.06926e4	2589.60791	95.3757

Fig.88. The HPLC for **14o**

Compound 14p

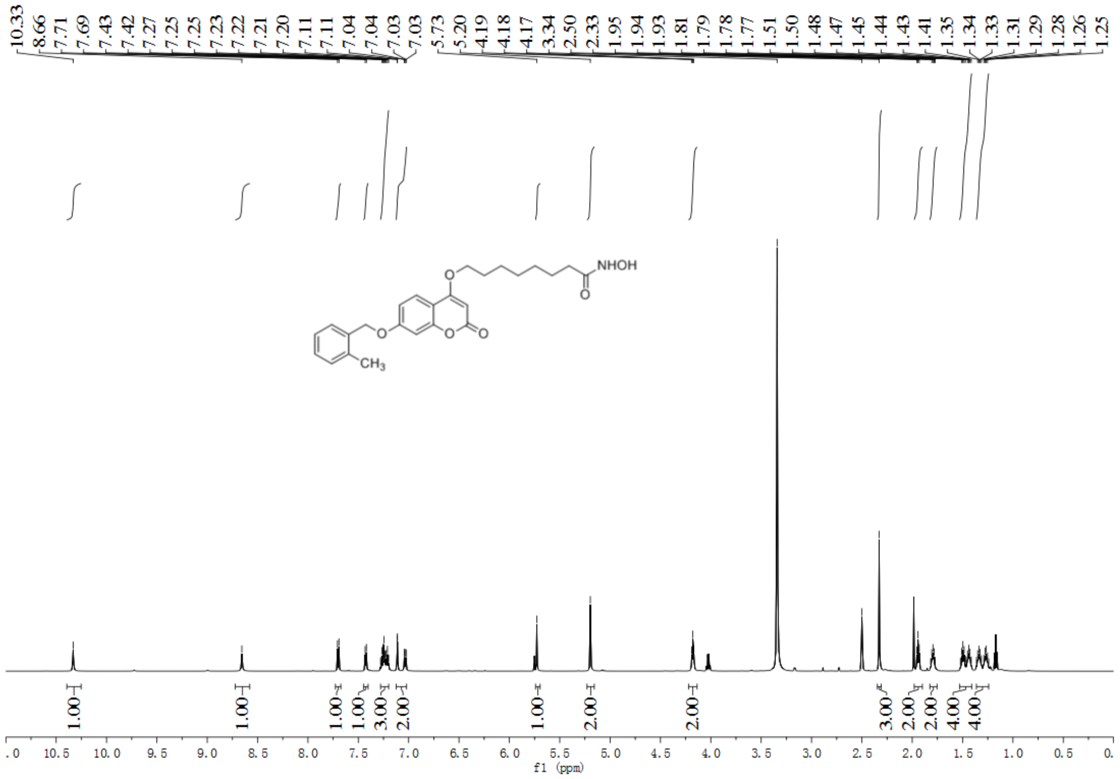

Fig.89. The ^{1}H NMR spectrum for **14p**

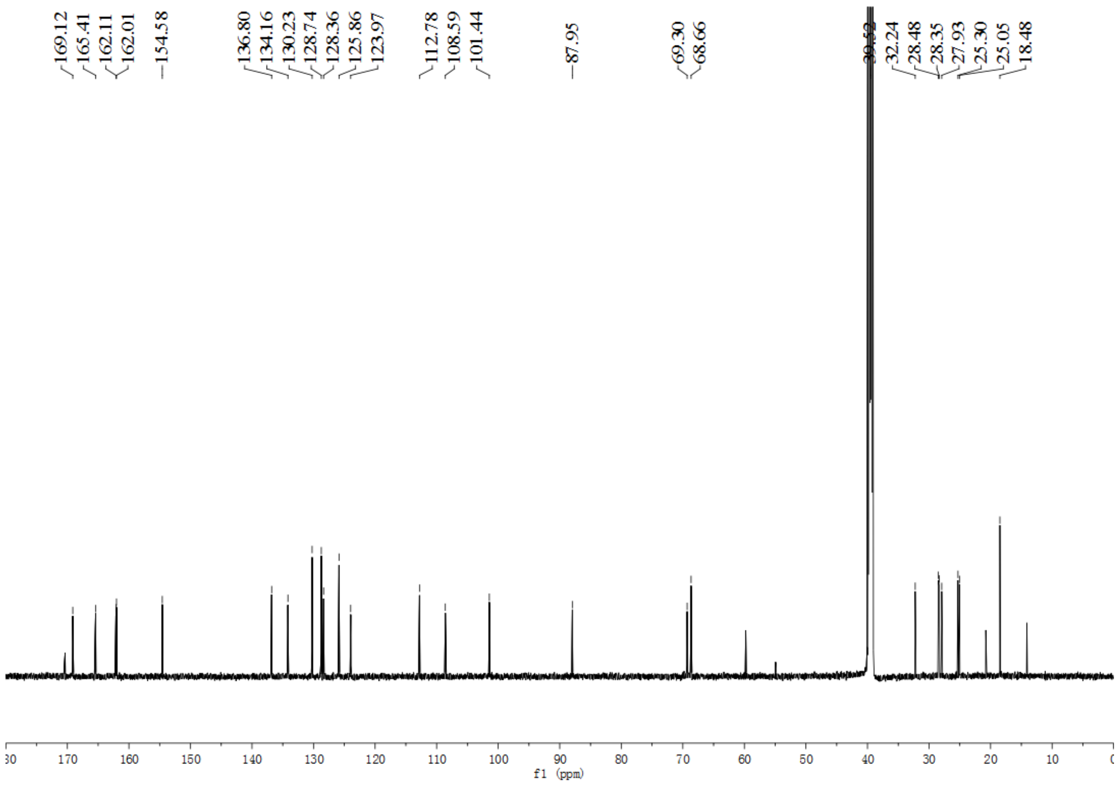

Fig.90. The ^{13}C NMR spectrum for **14p**

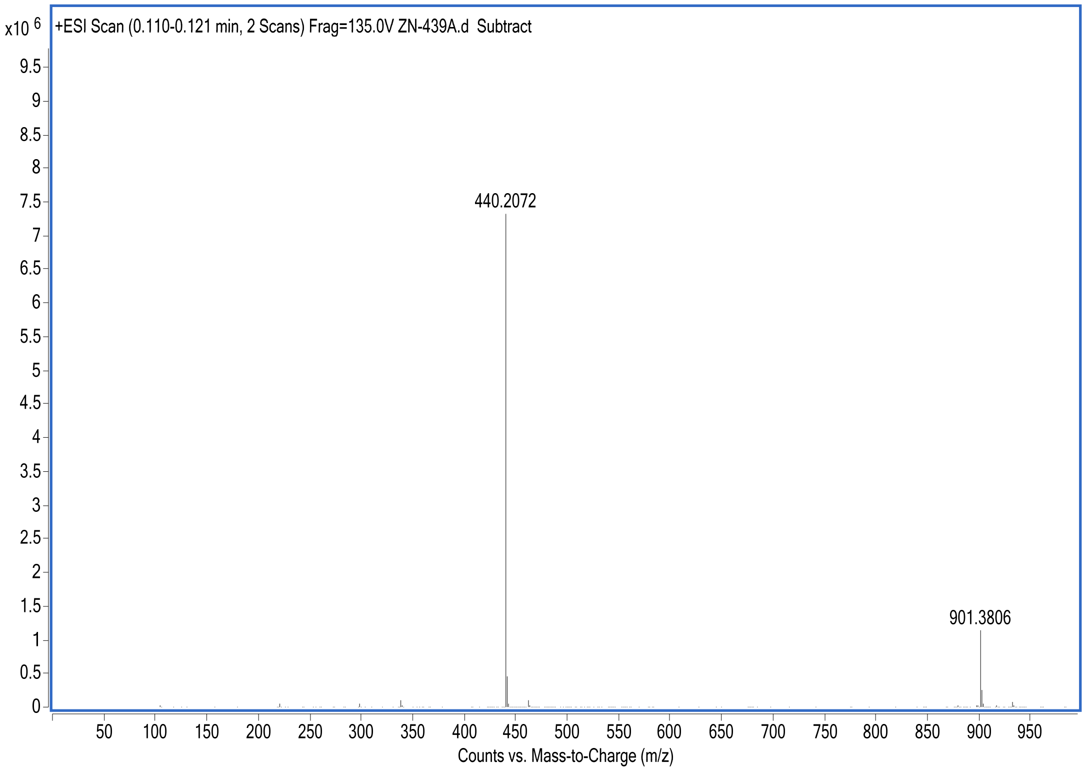

Fig.91. The HR MS spectrum for **14p**

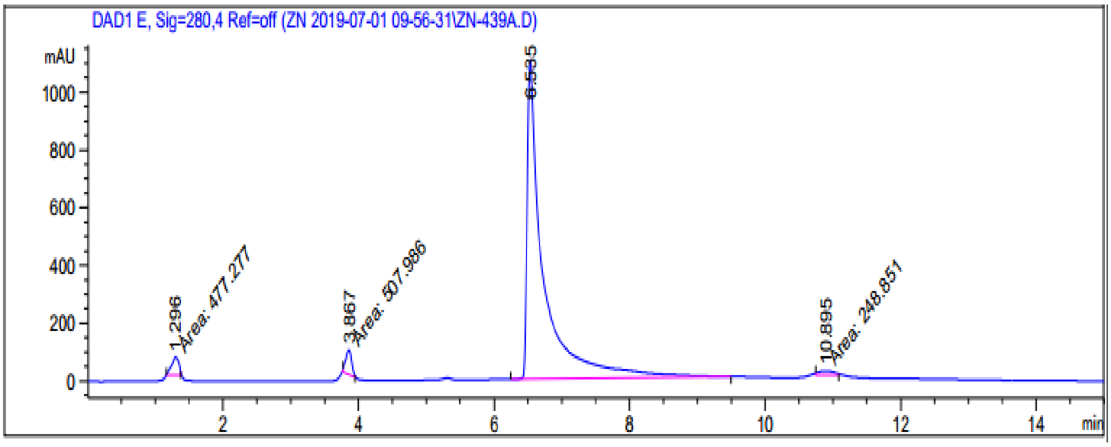

Peak #	RetTime [min]	Type	Width [min]	Area [mAU*s]	Height [mAU]	Area %
1	1.296	MM	0.1266	477.27661	62.82330	2.4023
2	3.867	MM	0.0995	507.98602	85.07893	2.5568
3	6.535	BB	0.2252	1.86336e4	1102.63391	93.7883
4	10.895	MM	0.2642	248.85106	15.69951	1.2525

Fig.92. The HPLC for **14p**

Compound 14q

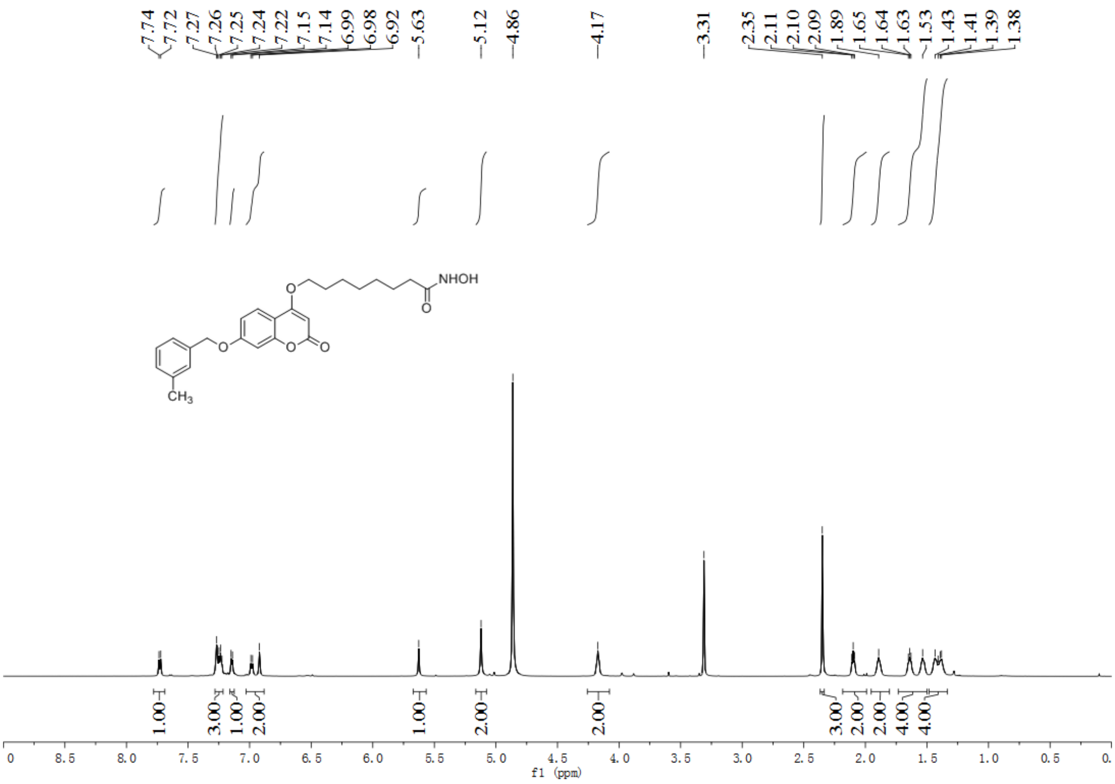

Fig.93. The ^{1}H NMR spectrum for **14q**

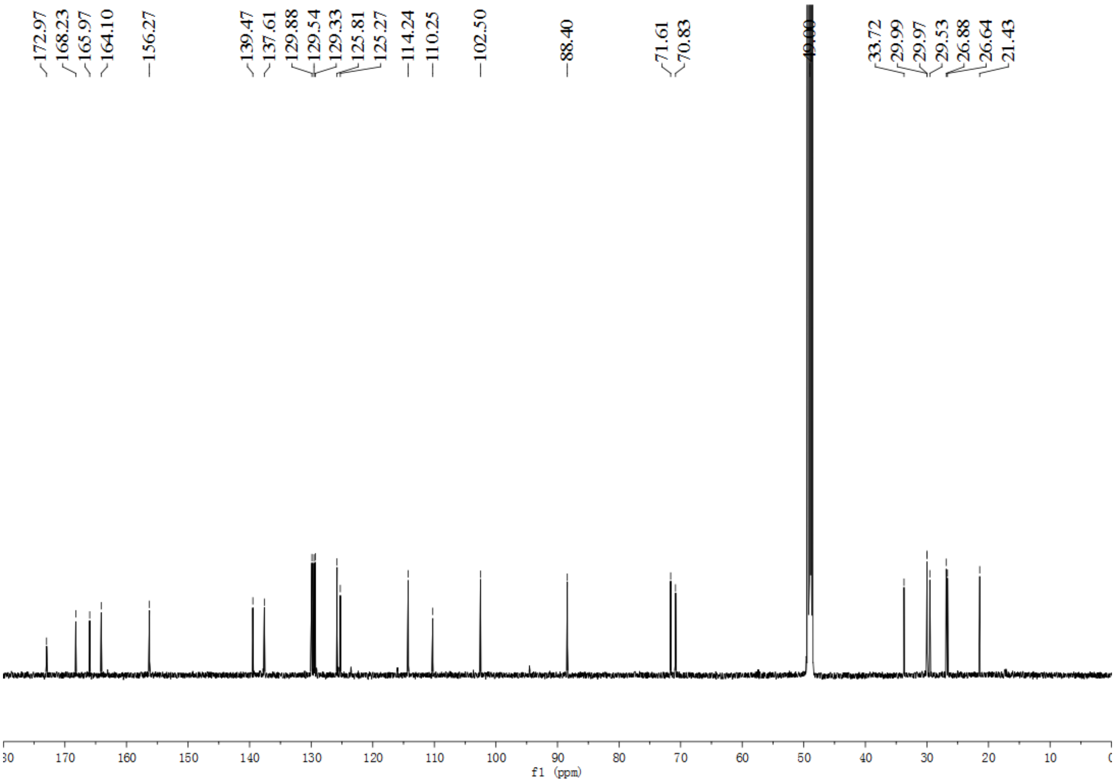

Fig.94. The ^{13}C NMR spectrum for **14q**

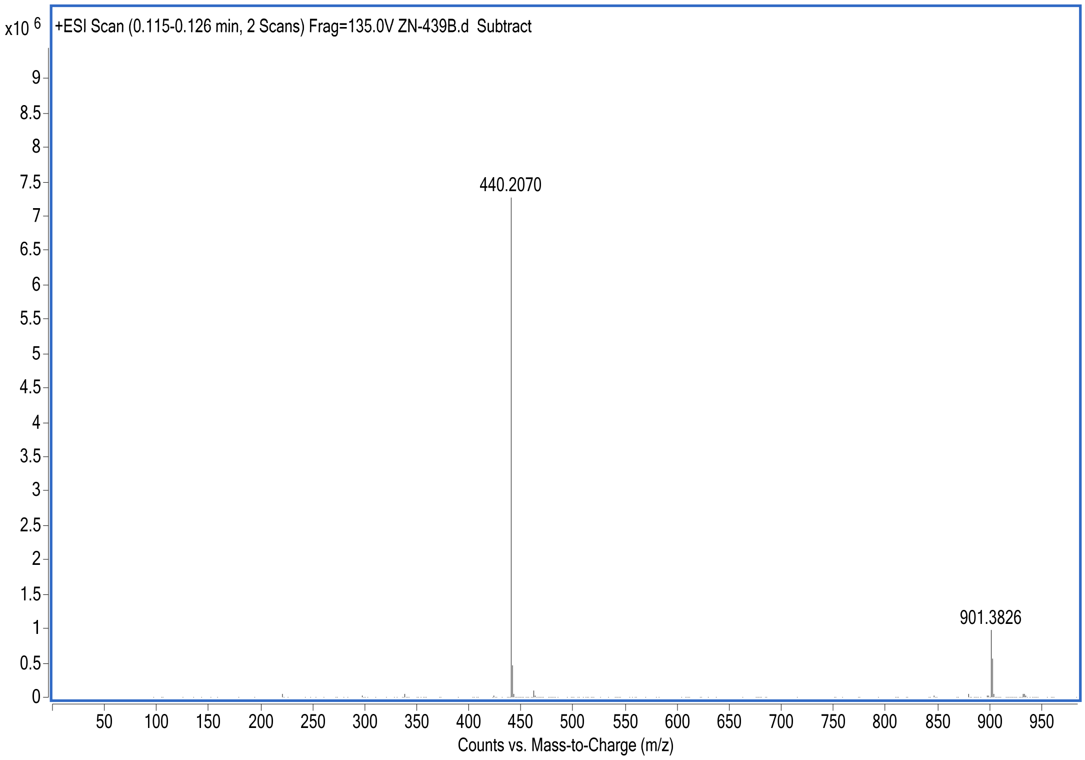

Fig.95. The HR MS spectrum for **14q**

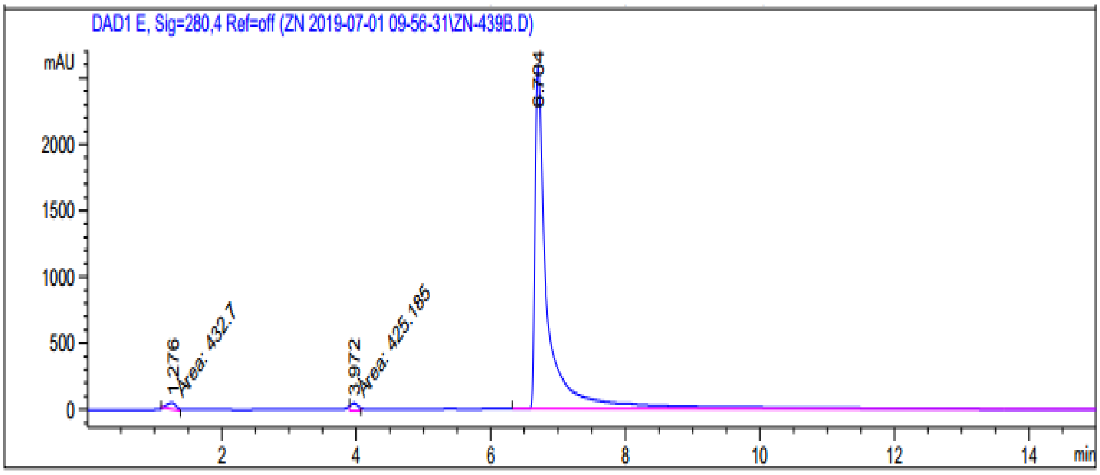

Peak #	RetTime [min]	Type	Width [min]	Area [mAU*s]	Height [mAU]	Area %
1	1.276	MM	0.1484	432.70010	48.60620	1.2108
2	3.972	MM	0.1202	425.18515	58.95599	1.1897
3	6.704	BV R	0.1765	3.48800e4	2579.91064	97.5995

Fig.96. The HPLC for **14q**

Compound 14r

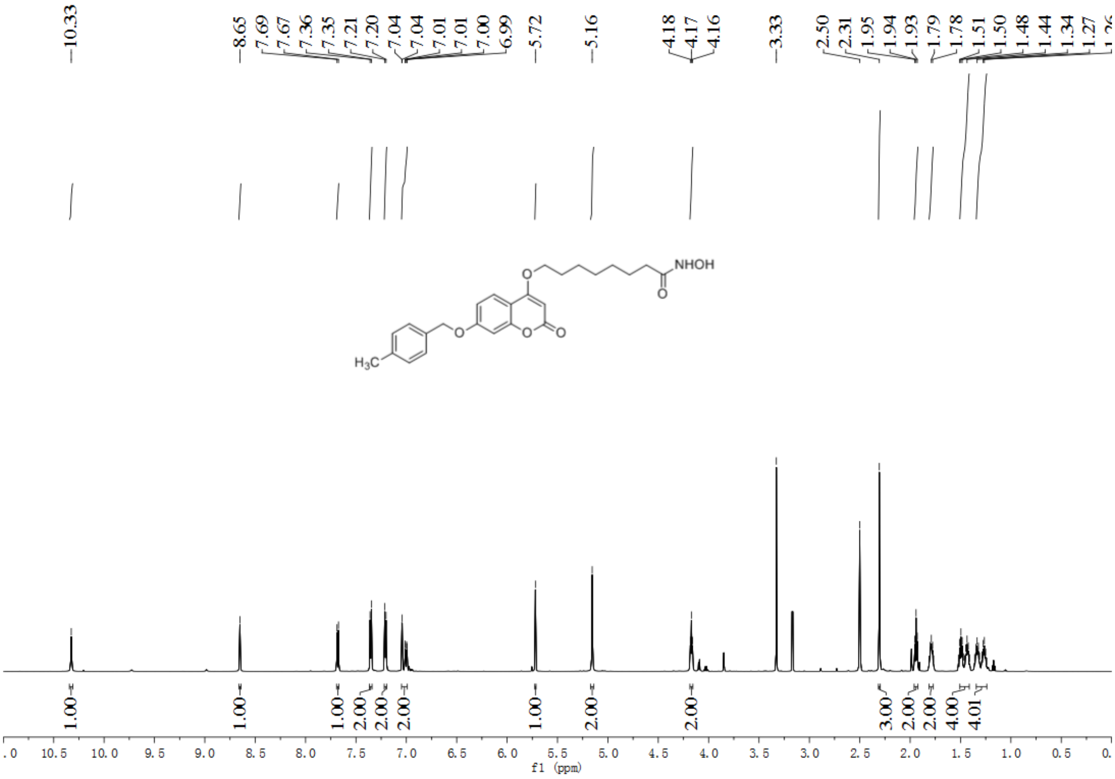

Fig.97. The ^{1}H NMR spectrum for **14r**

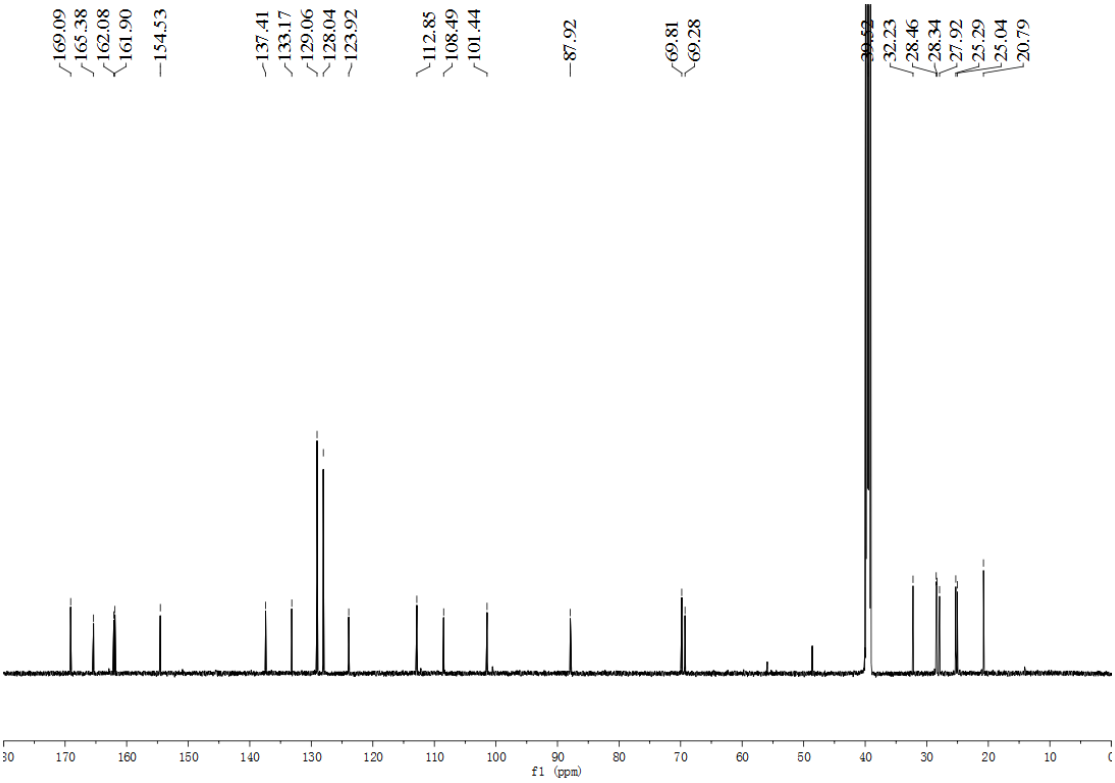

Fig.98. The ^{13}C NMR spectrum for **14r**

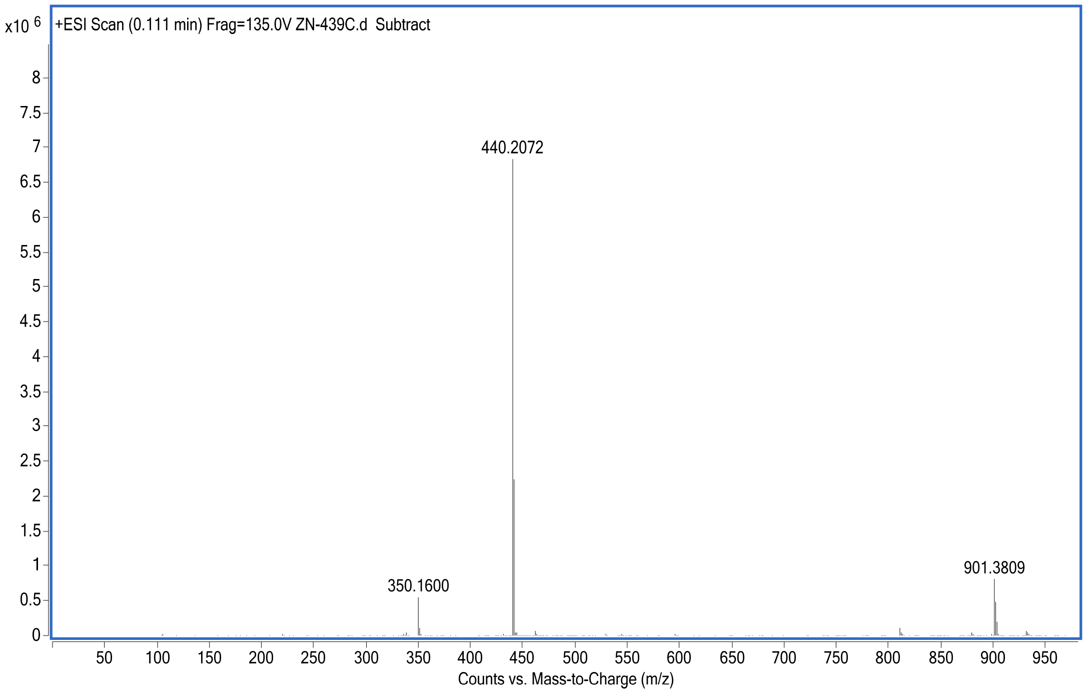

Fig.99. The HR MS spectrum for **14r**

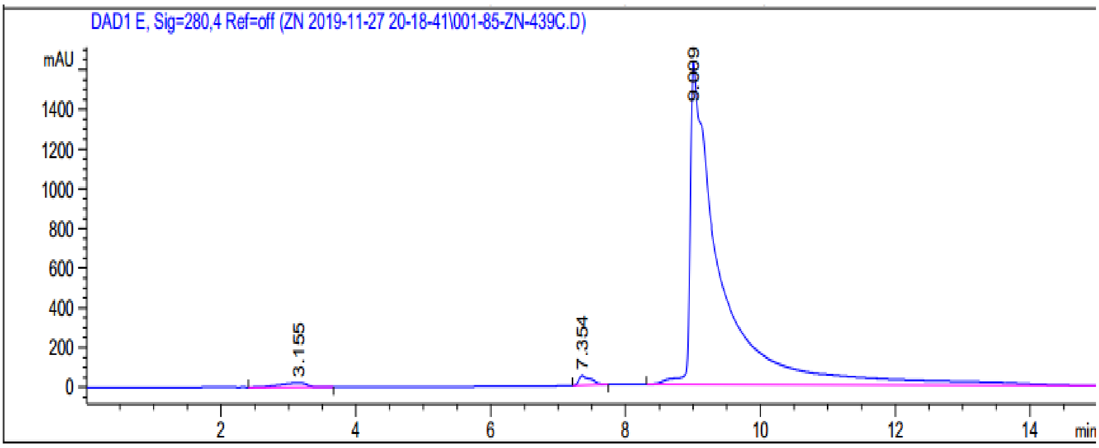

Peak #	RetTime [min]	Type	Width [min]	Area [mAU*s]	Height [mAU]	Area %
1	3.155	BB	0.4410	649.53705	23.31925	1.2362
2	7.354	BB	0.1664	602.76434	48.70610	1.1472
3	9.009	BBA	0.3956	5.12910e4	1619.24182	97.6166

Fig.100. The HPLC for **14r**

Compound 14s

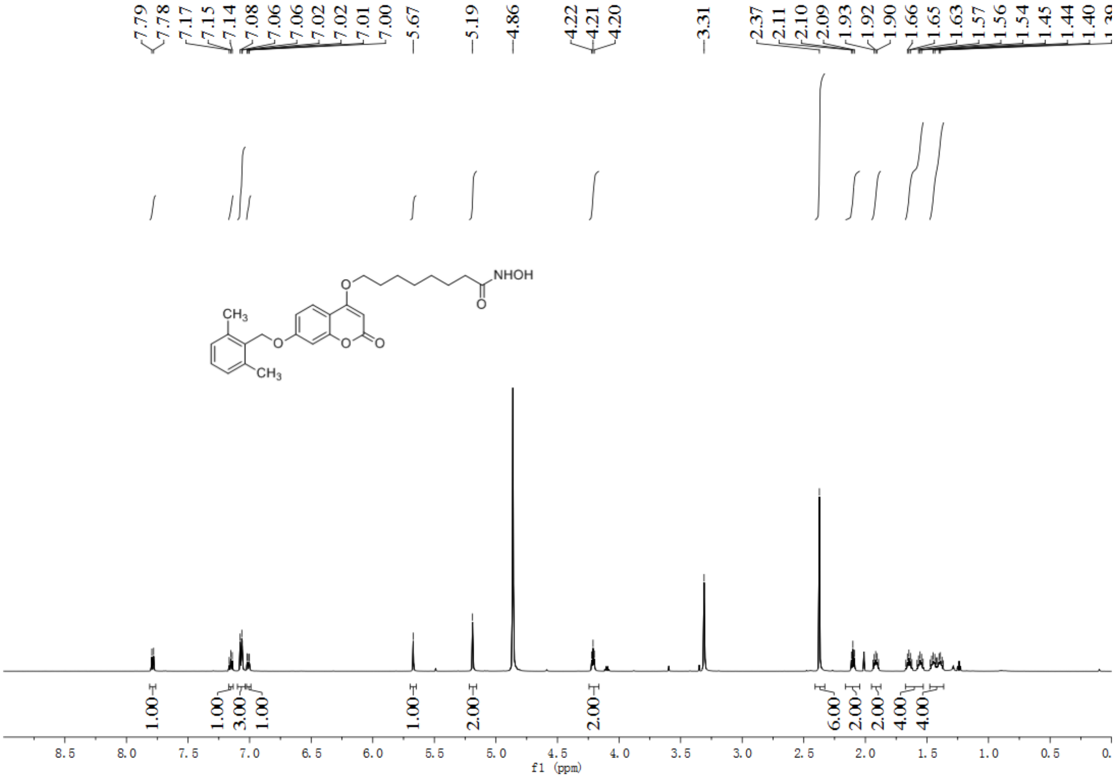

Fig.101. The ^{1}H NMR spectrum for **14s**

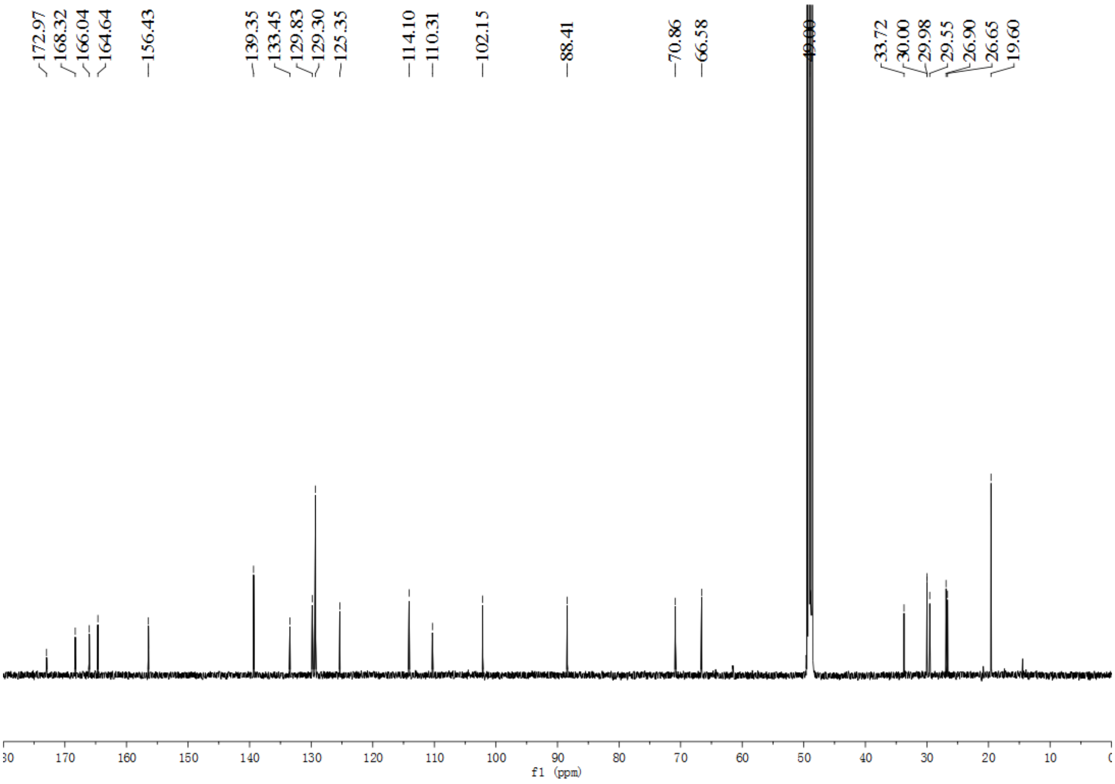

Fig.102. The ^{13}C NMR spectrum for **14s**

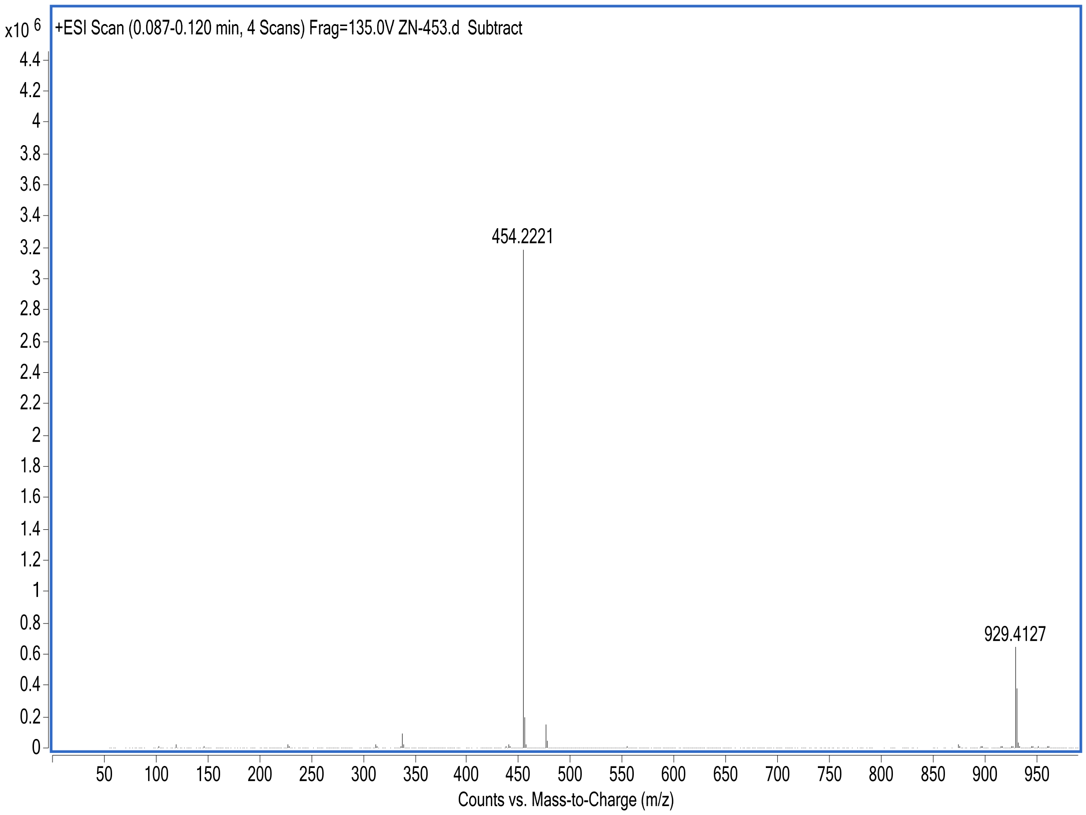

Fig.103. The HR MS spectrum for **14s**

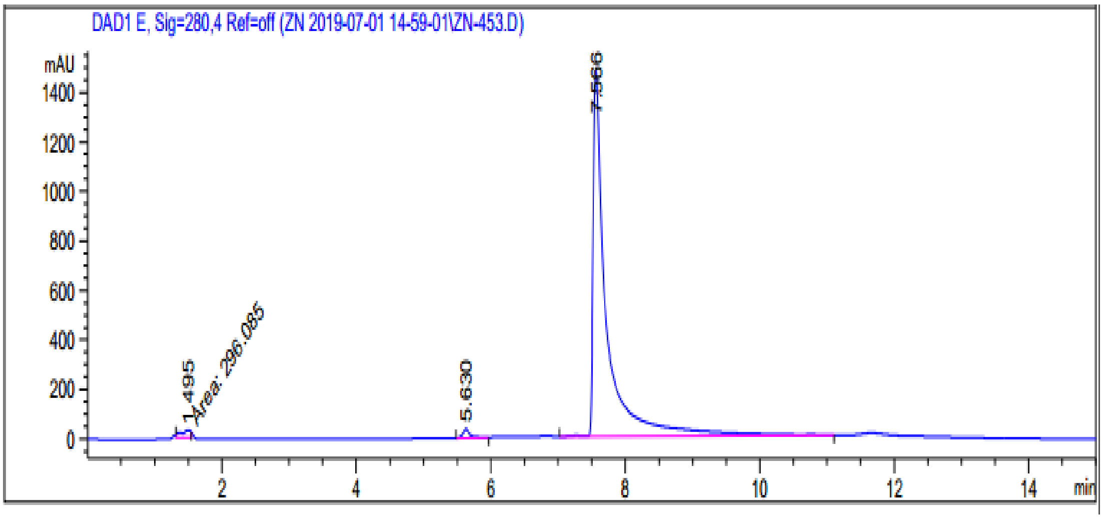

Peak #	RetTime [min]	Type	Width [min]	Area [mAU*s]	Height [mAU]	Area %
1	1.495	MM	0.1655	296.08493	29.81826	1.4137
2	5.630	BB	0.1024	242.80492	35.22078	1.1593
3	7.566	VV R	0.1862	2.04054e4	1482.34363	97.4270

Fig.104. The HPLC for **14s**
